# High-precision machine learning identifies a reproducible functional connectivity signature of autism spectrum diagnosis in a subset of individuals

**DOI:** 10.1093/gigascience/giaf091

**Published:** 2025-09-03

**Authors:** Natasha Clarke, Sebastian Urchs, Hien Duy Nguyen, Clara Moreau, Christian Dansereau, Angela Tam, Alan C Evans, Lune Bellec

**Affiliations:** Centre de Recherche de l'Institut Universitaire de Gériatrie de Montréal, QC H3W 1W5 Montréal, Canada; Département de Psychologie, Université de Montréal, QC H2V 2S9 Montréal, Canada; Centre de Recherche de l'Institut Universitaire de Gériatrie de Montréal, QC H3W 1W5 Montréal, Canada; Montreal Neurological Institute and Hospital, McGill University, QC H3A 2B4 Montreal, Canada; School of Computing, Engineering and Mathematical Sciences, La Trobe University, VIC 3086 Bundoora, Australia; Institute of Mathematics for Industry, Kyushu University, Nishi-ku Fukuoka 819-0395, Japan; Département de Psychiatrie et d'Addictologie, Université de Montréal, QC H3T 1J4 Montréal, Canada; Sainte Justine Research Center,Université de Montréal, QC H3T 1C5 Montréal, Canada; Centre de Recherche de l'Institut Universitaire de Gériatrie de Montréal, QC H3W 1W5 Montréal, Canada; Centre de Recherche de l'Institut Universitaire de Gériatrie de Montréal, QC H3W 1W5 Montréal, Canada; Montreal Neurological Institute and Hospital, McGill University, QC H3A 2B4 Montreal, Canada; Centre de Recherche de l'Institut Universitaire de Gériatrie de Montréal, QC H3W 1W5 Montréal, Canada; Département de Psychologie, Université de Montréal, QC H2V 2S9 Montréal, Canada

**Keywords:** resting-state functional connectivity, autism spectrum diagnosis, transductive conformal prediction

## Abstract

**Background:**

Discovery of predictive biomarkers is essential for understanding the neurobiological underpinnings of autism spectrum diagnosis (ASD) and improving identification. Resting-state functional connectivity analyses of individuals with ASD have established sensitivity of brain connectivity at the group level. However, the extensive heterogeneity in ASD limits the translation of these findings into reliable individual-level biomarkers. We analyzed the Autism Brain Imaging Data Exchange 1 and 2 datasets, calculating Pearson’s correlation-based functional connectivity across 18 brain networks. Using transductive conformal prediction, a machine learning approach that assigns confidence scores to predictions based on conformality to known classes, we classified individuals with ASD and neurotypical controls.

**Results:**

By combining predictors into an ensemble using hierarchical agglomerative clustering, we identified a signature that confers a more than 7-fold increase in individual risk of ASD, yet is still identified in an estimated 1 in 200 individuals in the general population. The individual risk conferred by the model is increased 4-fold over that of previously published imaging models and outperforms the current state of the art in precision for ASD classification. The high-risk signature was characterized by underconnectivity of transmodal brain networks, including the frontoparietal and basal ganglia network, and subcomponents of the limbic and default mode networks.

**Conclusions:**

A highly targeted prediction model can identify a subset of functional connectivity alterations that confer high risk for ASD at the individual level, which may be masked by traditional machine learning models due to ASD heterogeneity. Results could help disentangle the multitude of etiological pathways and behavioral symptoms that challenge our understanding of ASD by focusing on highly penetrant connectivity signatures.

## Introduction

Autism spectrum diagnosis (ASD) is a complex neurodevelopmental condition diagnosed in approximately 1% of the general population [1], characterized by impairments in social interaction and repetitive behavior [2]. ASD has been linked to changes in brain structure and function, as well as genetics, and is highly heritable, with an estimated heritability of 80% [1]. Despite the high heritability, there is wide heterogeneity in both symptoms and genetics [3] and extensive overlap with other neurodevelopmental disorders, such as attention-deficit/hyperactivity disorder and schizophrenia [4–6].

Discovery of predictive biomarkers is a fundamental aim in clinical neuroscience and may help decompose the marked heterogeneity in ASD. Biomarkers are critical for unraveling the neurobiological mechanisms underlying ASD, finding novel treatment targets, and identifying individuals who may benefit from these interventions [7]. An ideal biomarker with the potential to guide clinical decision-making at the individual level should combine 2 criteria: first, they should have high penetrance, conferring substantially increased ASD risk above the baseline for an individual with unknown ASD status. In machine learning, which offers valuable techniques for biomarker identification, this can be estimated using the positive predictive value (PPV). Second, biomarkers should have a high enough prevalence in the population to enable investigation in large cohort studies.

To date, most progress in biomarker detection for ASD has come from the field of genetics. “Genetics-first” studies have identified rare mutations such as copy number variants (CNVs) [8]—deletions or duplications of DNA segments that have large effects. However, applications of CNVs as a biomarker are limited by their low prevalence, typically occurring in fewer than 0.01% of individuals [9]. Conversely, common genetic variants such as single-nucleotide polymorphisms (SNPs) are found in more than 5% of the general population but have very low penetrance, conferring only a slight increase in ASD risk. The lack of a genetic mutation that demonstrates moderate prevalence and penetrance, despite the high heritability observed in ASD, has been termed the “missing heritability” gap [10, 11]. This suggests the need for alternative biomarkers.

Resting-state functional connectivity (FC), measured by functional magnetic resonance imaging (fMRI), is sensitive to brain organization in ASD [12, 13] and may offer another avenue to identify high-risk markers more common in the general population. MRI is noninvasive and widely available, and FC is task-free, making it suitable for clinical populations. Many studies have used machine learning to detect predictive FC signatures in ASD, using a variety of FC metrics. Pearson’s correlation coefficient between the time series of 2 regions of interest, determined using an atlas, seed-based, or data-driven approach such as independent component analysis [14], has been used as input to different machine learning models, revealing disruption to distributed networks in ASD. Common algorithms include support vector machines (SVMs), with classification accuracies reported of around 67% and 79% [15–17]. In a direct comparison of SVM, random forest, and a neural network on the same data, a neural network slightly outperformed both SVM and random forest, at 70% accuracy [18], and in general deep learning classification approaches for ASD likely outperform single-layer algorithms [19]. At a local scale, regional homogeneity (ReHo), which measures FC between a voxel or region and its nearest neighbors, has shown comparable results to Pearson correlation [20, 21]. Compared to these static FC techniques, dynamic FC uses sliding windows to analyze how FC fluctuates over time. These features have been found to outperform static FC in ASD classification, combined with a SVM, logistic regression [22, 23], or an ensemble classifier [24]. Here we focus on the common Pearson correlation FC, which captures both short- and long-range connectivities, and compared to dynamic FC is less computationally expensive and more easily interpretable.

Previous studies have faced significant challenges that impede the identification of reliable ASD biomarkers. Collection of MRI data is costly and time-consuming and particularly problematic for machine learning studies, which can overfit to noise in the training data. Indeed, accuracies increase as samples decrease, indicating bias [25]. Overfitting also increases with the ratio of features to samples, a problem for studies using high-dimensional fMRI data. The ability of a model to generalize, for example, to data collected at other sites is key for robust biomarkers, but many studies do not report generalization to an independent sample. Even for models that perform well in cross-validation, generalization to completely unseen data leads to a drop in performance [26]. Thus, although initial small, single-site studies showed good accuracy for ASD prediction, performance in large, multicenter cohorts has been lower, likely due to a combination of inflated performance estimates on the smaller samples [27] and clinical heterogeneity of ASD [28, 29].

Heterogeneity in ASD arises from multiple sources, including behavioral symptoms, cognitive skills, genetics, and brain alterations, giving rise to distinct subtypes. Research using CNVs has helped shed light on this heterogeneity since CNV-related brain alterations exhibit “mirror effects” on brain connectivity, with deletions and duplications affecting the same imaging measures in opposite directions [6]. This phenomenon may give rise to subgroup formation within idiopathic ASD cohorts, contributing to symptom heterogeneity. Clinically, distinction of subtypes has been challenging, instead leading to a focus on ASD as a spectrum [2]. Large-scale studies of brain alterations in ASD, supported by data-sharing initiatives such as the Autism Brain Imaging Data Exchange (ABIDE) [30], have been able to decompose this source of heterogeneity to reveal subtypes that map better to a continuous spectrum of discrete categories [31–33], suggesting promise for reliable FC biomarkers. FC patterns associated with such subtypes reveal idiosyncratic profiles that only exist in subsets of people with ASD [31, 34]. Crucially, machine learning studies that collapse results across heterogeneous samples likely obscure these more predictive signatures, hampering the penetrance potential of traditional imaging biomarkers.

Additionally, typical machine learning studies use a similar number of control and ASD participants to train and evaluate their models, which does not accurately reflect the risk of ASD in the general population, where only 1 in 90 people has ASD. Even with high prediction accuracy, this translates to low PPVs of around 2.4% to 2.2% [15, 18], not much higher than the baseline risk of 1–2% for ASD and comparable to common genetic mutations. A recent ensemble predictor from an ASD biomarker challenge reframed ASD classification to make a confirmatory diagnosis, by enforcing a low false-positive rate and thus high specificity [26]. As the prevalence of ASD in the general population is low, high model specificity is important to achieve a high PPV, and so this approach resulted in a PPV of 8.6% in an estimated general population sample. However, this impressive result was achieved through a complex public prediction challenge, in which the top 10 of 146 submissions were combined into an ensemble predictor, making it challenging to apply elsewhere.

In this study, we aim to identify a “brain-first” imaging signature that is more penetrant than existing imaging markers and common genetic variants, but with a relatively higher prevalence. To achieve this, we reframe the traditional prediction problem from optimizing the prediction accuracy across all individuals with ASD to instead optimizing the PPV, by focusing on individuals who we can predict with a high degree of confidence. To assess the degree of confidence in our predictions, we use a rigorous statistical framework designed for this purpose called transductive conformal prediction (TCP) [35, 36]. TCP explicitly computes the confidence in the clinical label predicted for each individual and uses these estimates to limit predictions to individuals for whom there is a very high level of confidence. Since it is transductive, it uses both training and test data to predict individual examples, rather than building a general model. This allows it to adapt to the distribution of the data, reducing overfitting and improving robustness to heterogeneity, particularly useful for disorders like ASD, where generalizability is a challenge. Conformal prediction approaches are relatively underused [37] but have been applied in clinical research to give a reliable measure of prediction uncertainty in drug discovery [37], tumor biopsy [38], and conversion to dementia [39], as well as with neuroimaging data in stroke risk [40] and clinical depression [41], but to our knowledge, they have not been applied in ASD. We use a large discovery sample to identify the potential high risk signature and validate it in a large replication sample, including estimating its prevalence and PPV in the general population. Finally, we report the connectivity and symptom profiles of individuals flagged by the signature. We hypothesize that by limiting predictions to the most confident cases, we will identify subsets of ASD individuals who share very predictive, high-risk FC signatures. We further hypothesize that the FC of different brain networks may give rise to distinct high-risk FC signatures.

## Results

### Individual networks do not predict ASD with high PPV

**Figure 1:**
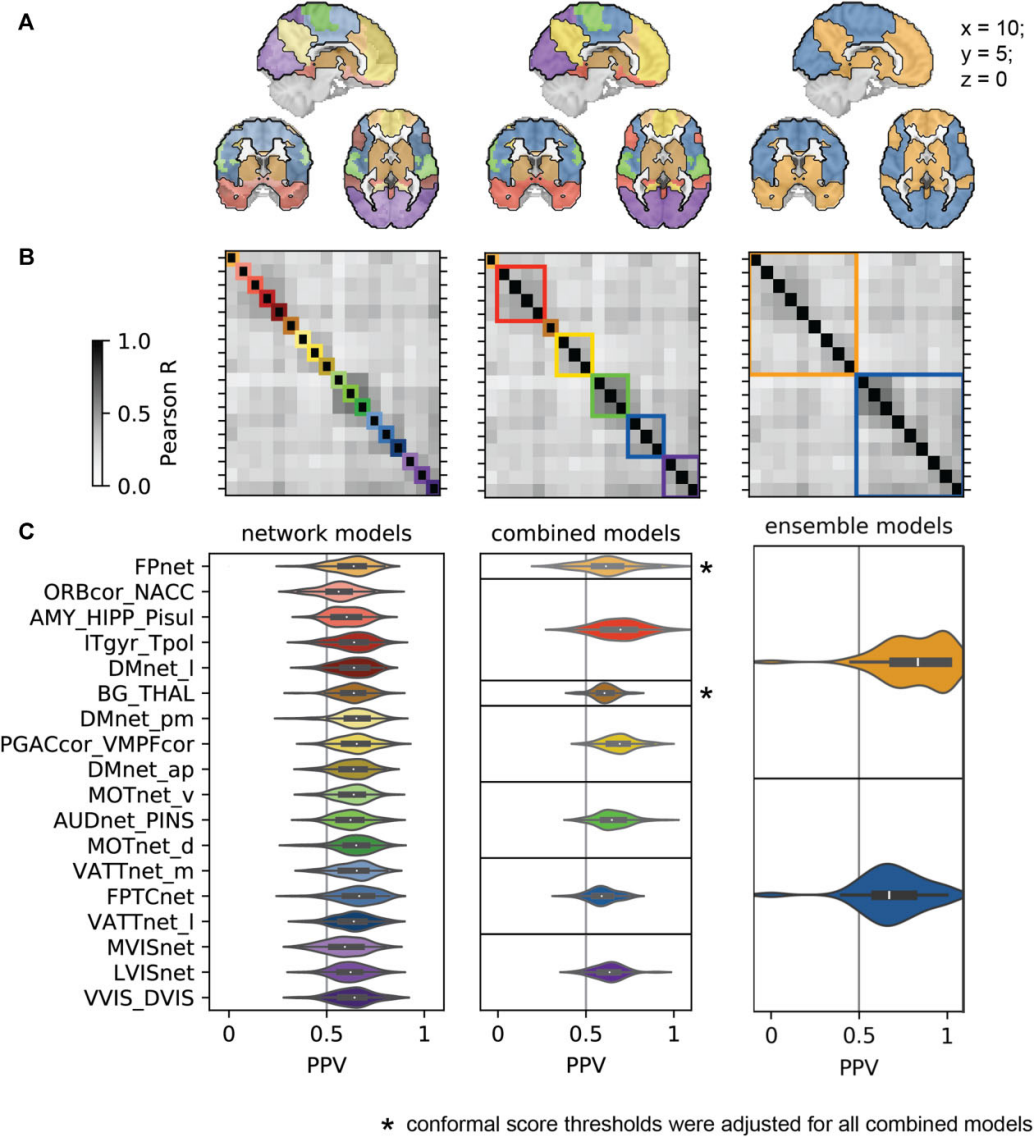
Combining network predictors with correlated conformal scores results in higher prediction performance. Figure shows the process of combining network predictors with correlated conformal scores to enhance the prediction performance for ASD. Left column = individual network models, middle column = combined models, right column = ensemble models. Individual networks (left column) were first clustered into combined predictors based on correlated conformal scores, using hierarchical agglomerative clustering of pairwise correlations of nonconformity scores (B, middle). Seven clusters were identified based on visual inspection of the correlation matrix, representing large-scale functional networks (A, middle). Networks with correlated conformal predictions were further clustered into 2 large ensemble predictors (B, right) that combined predominantly unimodal (blue) and transmodal (orange) brain networks respectively (A, right). The PPVs associated with conformal predictions for each model are shown in the bottom row (C). They are lowest for the individual networks and increase across combined and ensemble models. Predictions of the ensemble of more transmodal networks (orange) gave rise to a high-risk signature that predicted ASD with high positive predictive value (C, right).

We first evaluated the PPV of conformal ASD diagnosis predictions made with high confidence, based on the FC of each of the 18 brain networks (i.e., their FC was very atypical for neurotypical controls [NTCs], with a conformal score <5%, and not very atypical for ASD, with a conformal score >5%). To do so, we computed the median PPV of high-confidence conformal predictions for each brain network across 100 bootstrap samples (bootstrap PPV) of the discovery data. The bootstrap PPV of high-confidence conformal ASD diagnosis predictions ranged from 58% (orbitofrontal network) to 66% (default mode network) and was 62% on average across all networks. That is, among the individuals predicted with high confidence to have an ASD diagnosis, 62% on average did have an ASD diagnosis. As expected, the predictions were made with high specificity (91% on average across all networks) and low sensitivity (15% across all networks). That is, on average, 91% of NTC individuals were correctly not predicted to have an ASD diagnosis, and 15% of ASD individuals were correctly predicted to have an ASD diagnosis. Figure 1 shows an overview of the bootstrap PPV across networks. We thus showed that high-confidence predictions of ASD diagnosis made by individual brain networks did not lead to predictions with high PPV.

### Functionally similar brain networks predict correlated conformal scores

We investigated whether groups of brain networks gave rise to similar conformal predictions of ASD diagnosis and could be combined to achieve more accurate group predictions. We computed correlations between ASD conformal scores from individual brain network predictors and applied hierarchical agglomerative clustering, resulting in 7 groups: group 1 was a single network group of the frontoparietal network, group 2 combined limbic and temporal networks (orbitofrontal cortex, inferior temporal sulcus, lateral default mode network [DMN], and amygdala–hippocampal complex), group 3 was a single network group containing the basal ganglia network, group 4 combined subcomponents of the DMN (anterior- and posterior-medial DMN, perigenual anterior cingulate, and ventromedial prefrontal cortex), group 5 combined unimodal sensory networks (ventral and dorsal somatomotor network, auditory network), group 6 combined attention networks (medial ventral and lateral ventral attention network, frontoparietal task control network), and group 7 combined visual networks (medial, lateral, and downstream visual network). We thus show that functionally similar brain networks tended to give rise to correlated conformal predictions of ASD diagnosis.

We combined conformal scores from brain networks within each group to generate high-confidence ASD predictions and evaluated over 100 bootstrap samples (see Methods for details). The median across bootstrap samples was used, as the distribution of PPV values was not normally distributed (see Supplementary Fig. S1). The average bootstrap PPV across all groups was 64.4%, with high specificity (89%) and low sensitivity (18.4%). Group PPVs were similar to the average PPV of individual networks within them (group 2: 70% vs. 61.5%; group 4: 67.3% vs. 64.3%; group 5: 64.4% vs. 63.1%; group 6: 58.8% vs. 61.7%; group 7: 62% vs. 60.9%). Single network groups (groups 1 and 3) had adjusted PPVs (group 1: 65.1% vs. 64.1%; group 3: 61.4% vs. 61.1%) (see Table 1). Thus, groups of brain networks with correlated conformal scores predicted ASD with only marginally higher PPV than individual networks.

**Table 1:** PPV = positive predictive value. Group predictors showed no or only marginal improvements over the average PPV of their constituent network(s). Ensemble predictors achieved higher PPV than the average of their constituent networks, with ensemble 1 forming the high-risk ASD signature.

Combined predictors	PPV	Average PPV of constituent networks	Constituent network(s) of predictor
Group 1	65.1%	64.1% (adjusted PPV)	Single network: frontoparietal network
Group 2	70.0%	61.5%	Limbic and temporal networks
Group 3	61.4%	61.1% (adjusted PPV)	Single network: basal ganglia network
Group 4	67.3%	64.3%	Sub-components of the DMN
Group 5	64.4%	63.1%	Unimodal sensory networks
Group 6	58.8%	61.7%	Attention networks
Group 7	62.0%	60.9%	Visual networks
Ensemble 1 (HRS)	83.4%	62.7%	Nine more transmodal networks from groups 1-4
Ensemble 2	67.2%	63.0%	Nine more unimodal networks remaining from groups 5-7

### Ensemble of transmodal networks forms high-risk ASD signature

We further combined brain networks with correlated conformal scores into 2 large ensemble predictors. Ensemble 1 included 9 more transmodal networks from groups 1–4 (frontoparietal, limbic, basal ganglia, DMN), and ensemble 2 included 9 more unimodal networks remaining from groups 5–7 (sensorimotor, attention, visual). Predictions were evaluated across 100 bootstrap samples. Ensemble 1 had a PPV of 83.4%, higher than its group predictors’ average (62.7%). Ensemble 2 had a PPV of 67.2%, also higher than its group predictors’ average (63%) (see Table 1). Ensemble 1 showed higher specificity (99%) and lower sensitivity (5%) compared to ensemble 2 (specificity 97%, sensitivity 7.5%). Combining all networks into a whole-brain model did not improve PPV (76.6%). We thus demonstrated that combining correlated network predictions into ensemble predictors (specifically, ensemble 1) produced a robust high-risk signature (HRS) for ASD diagnosis and chose to further investigate ensemble 1’s high PPV signature in the independent replication dataset.

### High-risk ASD signature generalizes to independent data

We assessed the generalizability of the HRS in an independent replication sample by computing conformal scores for each individual relative to the discovery sample. The HRS identified 10 individuals from 6 imaging sites in the replication sample, of whom 9 had an ASD diagnosis. The PPV of the HRS was 90% in the replication sample, similar to the discovery sample’s bootstrap PPV of 83.4%. Specificity (99.5%) and sensitivity (4.2%) were also consistent with the discovery sample (99% and 5%, respectively). Ensemble 2 showed similar results, with a PPV of 62.5% (discovery: 67.2%), specificity of 95.8% (discovery: 97.0%), and sensitivity of 7.1% (discovery: 7.5%). Thus, the high-risk ASD signature demonstrated similar predictive performance in an independent validation dataset.

### High-risk ASD signature translates to 7-fold risk increase in general population

The discovery and replication samples were balanced with equal numbers of individuals with ASD and NTCs (50% prevalence) for model training and evaluation. However, in an unselected population, ASD prevalence is estimated to be 1.11% (1 in 90). The HRS identified 4.2% of individuals with ASD (sensitivity) and had a 0.5% false-positive rate (1 – specificity). To estimate HRS performance in an unselected population, we calculated expected accuracy for an ASD prevalence of 1.11%. The HRS correctly identified 0.046% of the population (4.2% sensitivity × 1.11% individuals with ASD) and incorrectly identified 0.49% (0.5% false-positive rate × 98.89% individuals without ASD or with NTC), resulting in a PPV of 9.2% (using unrounded values). Thus, an individual identified by the HRS had an 9.2% risk of ASD or a 7.8-fold increase over the baseline risk.

### High-risk signature characterized by underconnectivity

**Figure 2:**
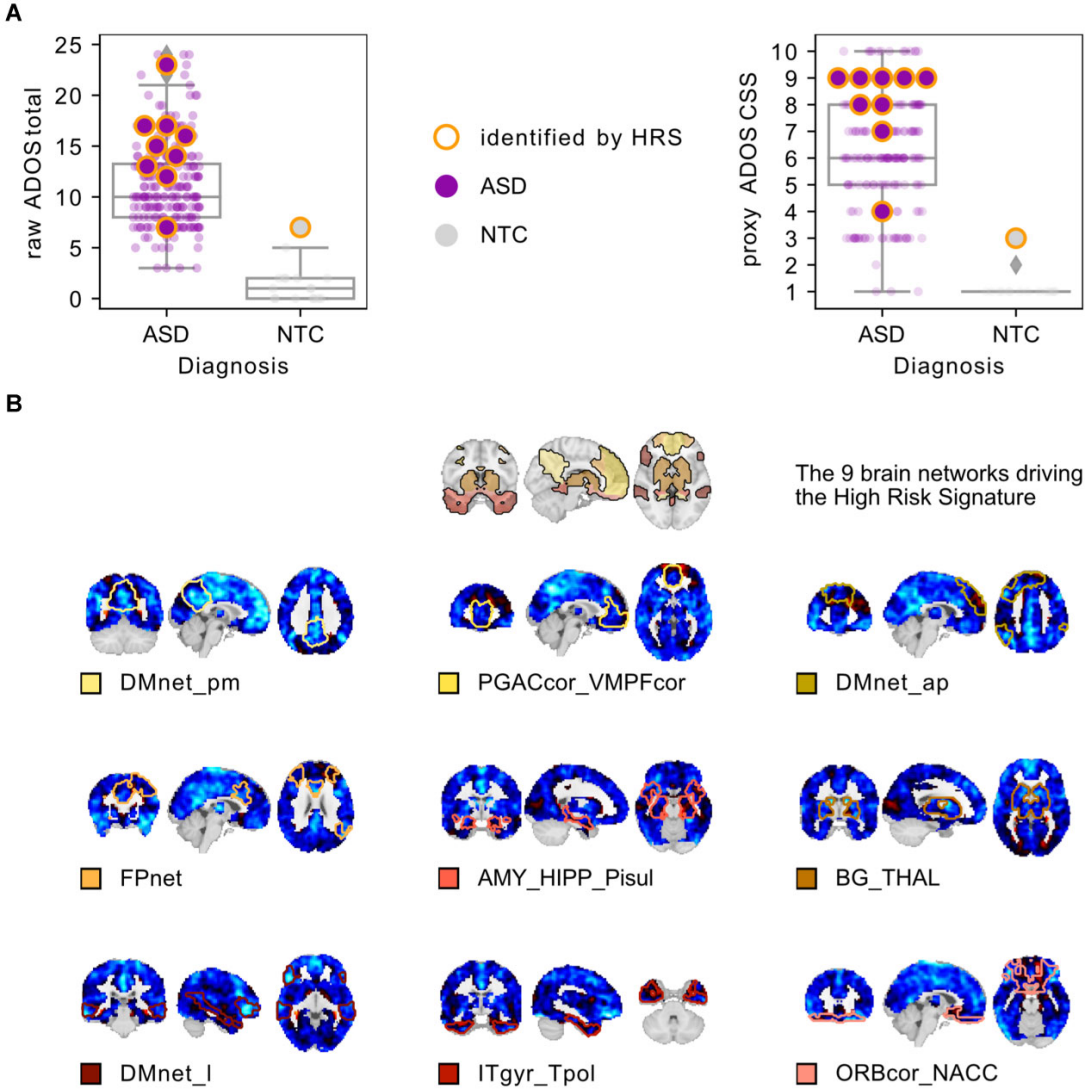
The high-risk signature tends to identify individuals with severe symptoms and pervasive underconnectivity. (A) Individuals identified by the high-risk signature (circles with orange outline) have high proxy calibrated ADOS severity scores (left plot) and high raw ADOS total scores (right plot) compared to the average of their respective diagnostic category. (B) The identified individuals share a pattern of distributed below-average functional connectivity of the 9 networks driving the high-risk signature (the networks are denoted by name and colored outline on their respective connectivity maps).

To identify the FC pattern of the individuals detected by the HRS model, we investigated the average residual connectivity maps of the identified individuals for the 9 brain networks contributing to the HRS. Figure 2B shows the average residual connectivity maps of the 9 networks, which are characterized by pervasive underconnectivity with respect to the rest of the discovery sample. We thus show that the FC signatures of individuals identified by the HRS model were characterized by widespread underconnectivity of the 9 involved brain networks with respect to the sample average.

### Conformal prediction not driven by nuisance covariates

**Figure 3:**
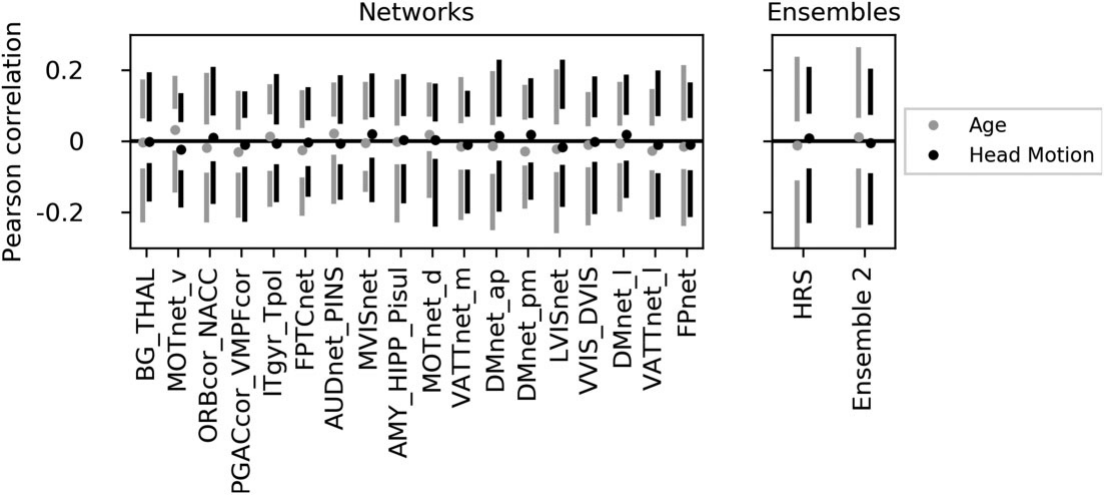
The conformal predictions are not driven by nuisance covariates. The distribution of correlations of ASD conformal scores predicted by individual networks (left) and the 2 ensemble models (right) with head motion (black) and age (gray) are shown across 100 bootstrap samples. Circles represent the median correlation score across bootstrap samples, and vertical lines span the 5th to 25th percentiles (lower bar) and 75th to 95th percentiles (upper bar) of correlation scores, respectively. All median correlation scores are close to zero and enclose zero within the 90% confidence interval.

For all network and ensemble predictors, ASD conformal scores showed no significant correlations with age or head motion, with confidence intervals including zero (Fig. 3). Thus, ASD conformal scores were not substantially influenced by nuisance variables. Medication use also did not differ between ASD individuals identified by the HRS model and those not identified (see supplementary materials, Results section).

### Conformal prediction performance exceeds baseline model

To determine if our FC-based predictive signature performed better than a simple baseline model, we repeated the conformal prediction procedure using an individual’s age and in scanner head motion as input features. Following the same procedure described above, we then used the transductive conformal prediction approach to predict an ASD diagnosis only for those individuals in whom the model had high confidence. Our results show that such a baseline model did not predict ASD diagnosis with high confidence, with a median sensitivity and PPV of zero (Supplementary Fig. S1). We thus show that the FC-based network predictors performed better than a simple baseline model.

### High-risk signature tends to identify individuals with severe symptoms

There was a weak positive correlation between symptom severity (Autism Diagnostic Observation Schedule [ADOS] proxy scores) and the ASD conformality score (Pearson’s *r* = 0.186, *P* = 0.005). Since only 10 individuals were identified by the model, further testing of symptom severity was limited. Exploratory analysis, detailed in the supplementary materials (Results section), indicated that the identified individuals tended to show particularly severe symptoms for their diagnostic class, but that, importantly, the model does not only identify those with severe symptoms.

## Discussion

This work aimed to identify an imaging biomarker of ASD that is both commonly found in the general population and confers a high risk of the disorder. Using a transductive conformal prediction approach, we identified individuals with high-confidence ASD predictions based on FC. Our results showed that combined predictions from 9 brain networks gave rise to a high-risk FC signature, identifying individuals with mostly severe symptoms, and pervasive underconnectivity in an independent dataset. Compared to genetic biomarkers, our brain-first signature demonstrated higher penetrance than common mutations and higher prevalence than rare CNVs.

### Model performance

**Figure 4:**
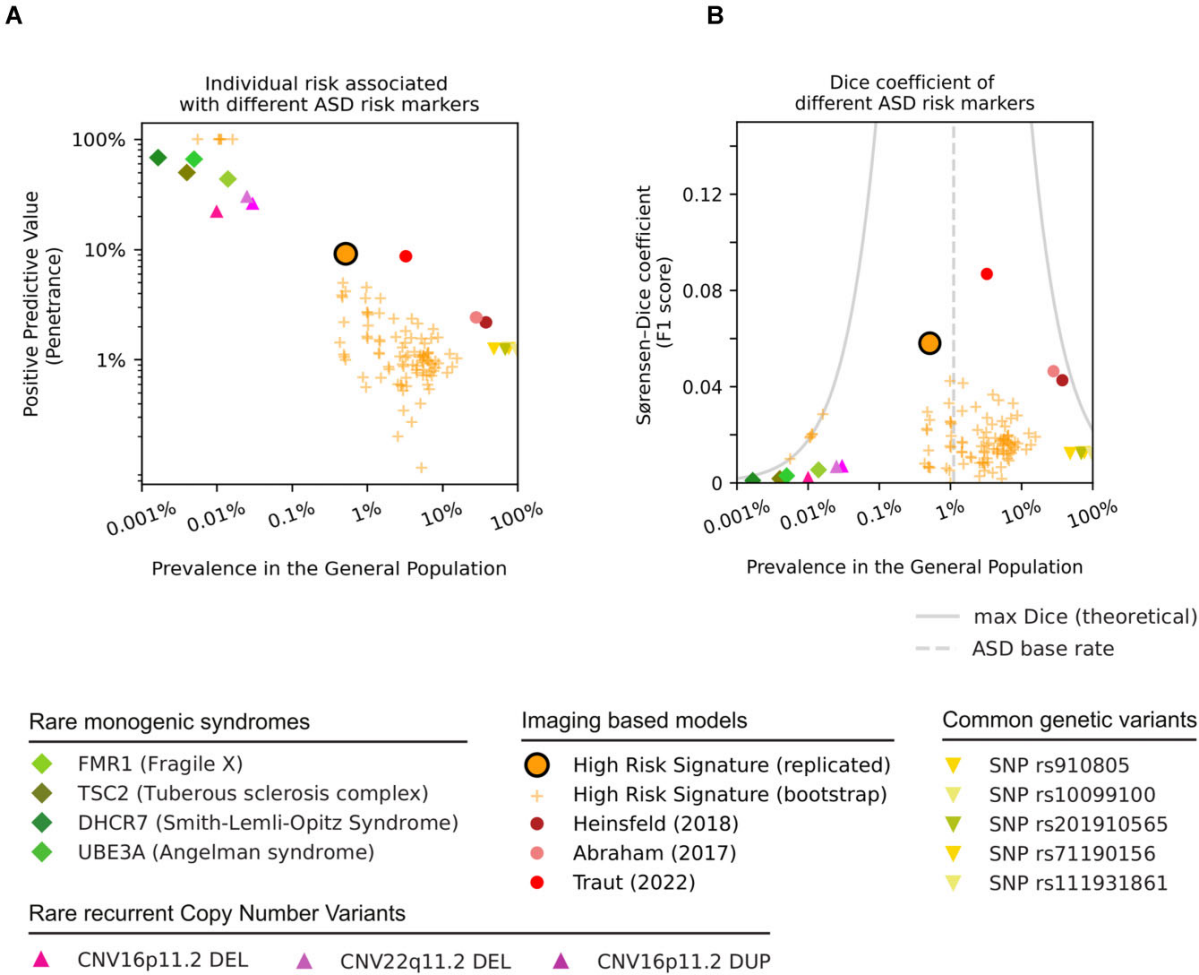
High-risk signature is more common than genetic risk markers, confers higher risk than traditional imaging models, and meets the current machine learning state-of-the-art. Monogenic syndromes (green rhombs) and recurrent copy number variants (pink triangles) confer high risk of ASD diagnosis (vertical axis) but are rare (horizontal axis). ASD-related SNPs (yellow triangles) are very common but confer negligible risk of ASD. Current imaging-based predictive models (2 pink circles) identify large portions of the general population with low risk of ASD. The high-risk ASD signature (orange, black outline) identifies a small portion of the general population with elevated risk of ASD diagnosis, concordant with the estimated performance in the discovery data (orange plus signs), meeting the positive predictive value of 10 machine learning models combined (red circle), using a simple model.

This multinetwork FC signature confers a PPV of 9.2% and a more than 7-fold increased risk of ASD diagnosis in the general population, where it is identified in an estimated 1 in 200 individuals, compared to a baseline ASD prevalence of 1 in 90 individuals. It is approximately 2 orders of magnitude less common than ASD-related SNPs [42], which confer negligible risk, and 2 orders of magnitude more common than rare monogenic syndromes [9], which confer very high risk (see Fig. 4). Studies using similar data and machine learning to classify ASD, but without the TCP approach, report accuracies that translate to PPVs of 2.4% to 2.2% [15, 18]. Our FC signature’s risk increase is therefore around 4 times higher than current neuroimaging models. We also outperform the current state-of-the-art in neuroimaging for achieving high ASD PPV (8.6, the result of a large ensemble biomarker challenge), but using a simple logistic regression-based approach that is easily scalable (Supplementary Fig. S2). To the best of our knowledge, no genetic risk signatures of autism offer comparable individual risk while being relatively common. Although similar polygenic risk signatures exist for other diseases [43], the few common ASD variants (e.g., only 5 ASD-specific SNPs [42] vs. 108 that have been identified for schizophrenia [22]) and the large sample sizes needed for robust polygenic risk estimation make these discoveries unlikely to happen soon.

Unlike previous imaging models that make predictions for all individuals in heterogeneous case-control populations, we limited predictions to a subset with very high-confidence ASD diagnoses. Although our model made relatively few predictions, they carried a higher risk of ASD, which, compared to traditional approaches [15, 18], resulted in higher specificity (99.5% vs. 72.3% and 63%, respectively) and lower sensitivity (4.2% vs. 61% and 74%, respectively). This trade-off is intentional and is a result of the TCP framework that prioritizes high-confidence cases.

In clinical prediction, the optimal trade-off between specificity and sensitivity depends on the goal. High specificity is prioritized if the cost of misclassification is high, such as the risk of inappropriate interventions, while high sensitivity is more valuable in contexts such as population screening. In the current work, by prioritizing specificity, the model identifies only the highest-confidence cases, which enables the discovery of a connectivity signature that confers high risk for ASD. This comes at the cost of lower sensitivity. We have not proposed a better machine learning model but rather addressed a different objective—the conformal prediction approach could yield similarly high specificity with previously published imaging models. Indeed, an ensemble model from an ASD prediction challenge [26] achieved a similar PPV (9.2% vs. 8.6%) but with higher sensitivity (25.4% vs. 4.2%). Our logistic regression predictor thus confers a higher individual risk than state-of-the-art models, with much less model complexity but at the cost of lower sensitivity. The conformal prediction approach can be applied to any predictor to target high-confidence predictions; it is agnostic to both feature type and algorithm. Given that SVM and deep learning approaches have been found to perform well for ASD classification [19], future studies could incorporate TCP with these approaches to improve the PPV. Similarly, TCP could be utilized in studies using other FC approaches, such as dynamic FC. Here we focus on a simple logistic regression model and static FC using Pearson correlation to improve scalability and interpretation, important for clinical applications. Overall, our results suggest that the emergence of more performant predictors opens the door to push the boundaries of high-risk signatures further in the future.

### The signature is driven by transmodal brain networks

Individually, the 18 brain networks did not predict ASD with high PPV. By clustering networks with correlated conformal scores and combining their predictions, we identified 2 sets of brain networks. The first gave rise to the high-risk ASD FC signature and included predominantly transmodal networks in the DMN and frontoparietal network, as well as subcortical areas [44]. Note that we distinguish between transmodal and unimodal networks in line with the processing hierarchy proposed by Mesulam [45], in which lower-order unimodal areas encode basic sensory features, while higher-order transmodal networks (e.g., DMN) integrate this information into domain-general cognitive representations. Our finding aligns with previous FC-based ASD prediction models, which identified similar transmodal areas such as the temporal parietal junction and frontoparietal control network [15, 22], cingulo-opercular network [46, 47], and regions within the supramarginal, middle temporal, and cingulate gyri [18]. FC alterations in transmodal networks, particularly in the DMN [48–50], have been consistently reported in ASD case-control studies [13, 51, 52].

The second ensemble, consisting mostly of unimodal networks in the visual, auditory, and somatosensory cortices involved in sensory processing and the ventral attention network, did not predict ASD with high PPV. Although FC alterations in unimodal areas are well documented in ASD [53, 54], they are generally less predictive of diagnosis than transmodal regions [18]. The distinction between unimodal and transmodal FC is well established [55–57], with opposing alterations in ASD. Transmodal regions are often overconnected, while unimodal regions are underconnected [13]. This reflects a cortical gradient of functional hierarchy [58] that is altered in ASD [31, 59], suggesting a dysfunctional separation between primary sensory networks and the DMN. Thus, both ensembles may capture distinct ASD risk signatures, but only 1 was reliably identified in our dataset.

### Individuals identified by the signature tend to have severe symptoms and underconnectivity

The high-risk FC signature identified 10 individuals from the independent validation dataset, 9 of whom had an ASD diagnosis. These individuals generally had high symptom severity. However, their ADOS scores overlapped with those not detected by the model, indicating that the signature does not only detect severe ASD (Fig. 2A). This was supported by only a weak correlation between symptom severity and ASD conformality scores. Notably, the 1 individual without an ASD diagnosis identified by the signature had unusually severe symptoms compared to other NTC individuals, possibly reflecting a broader autism phenotype that extends into the general population [60]. Thus, the signature may identify a subtype of ASD patients with particularly severe symptoms, which, since identification is based on strong dissimilarity with NTC, would be consistent with a view of neurodevelopmental disorders as a deviation from normal functioning [61, 62].

The identified individuals shared a profile of pervasive functional underconnectivity in transmodal networks that gave rise to the high-risk FC signature. While transmodal network dysconnectivity, especially in the DMN [52], is consistently reported in the ASD literature, its direction (over- or underconnectivity) varies [63, 64] and is related to increases in symptom severity [48, 65]. Notably, our finding of transmodal network underconnectivity contrasts with a case-control finding of reproducible, ASD-related prefrontal and parietal overconnectivity in a large, multicenter study [13]. These contrasting findings may highlight case-control studies’ limitations in identifying ASD-related FC subtypes. Indeed, recent studies also report transmodal underconnectivity in ASD subtypes [31, 32]. Our results align with other ASD prediction models that found underconnectivity between DMN subregions to be highly predictive [15, 18] (but see Yahata et al. [46]). It should be noted that while we limited our sample to males due to the strong sex imbalance and to ensure matching across sites, these studies included a small percentage of female participants. However, our results are also consistent with other research on males only [66–68].

### Limitations

These findings must be interpreted in light of their limitations. First, as mentioned, our analyses only included male individuals, a common problem in the field [59, 69] due to the higher frequency with which ASD is diagnosed among male individuals [70]. Efforts are underway to include more women in ASD cohorts [71, 72]. Second, behavioral and symptomatic characterization of those detected by the high-risk signature was limited by inconsistent availability of phenotypic information. Future studies with large-scale, complete phenotyping datasets are needed for a better understanding of the cognitive and symptom profiles of neurobiologically defined at-risk individuals. Third, our transductive conformal prediction model can only control for nuisance covariates available in both the reference sample and the predicted individual, so we were unable to account for site effects. However, the high-risk ASD signature identified individuals from different imaging sites with high PPV, suggesting robustness to site differences. Finally, we estimated the general population risk of our high-risk signature based on its performance in the independent dataset, identifying very few individuals (in line with our expectations). However, we were unable to explicitly test the signature on an unselected sample to empirically determine true performance. Validating risk signatures with such a low prevalence typically requires much larger datasets [43]. Recently available general population samples with imaging data [73] should be used to validate the high-risk signature and establish robust performance estimates.

### Future directions

The high-risk FC signature we have described offers interesting implications for future research. It identifies a cohort of individuals with similar FC alterations at high risk of an ASD diagnosis, a population in which to explore the link between neurobiological aberrations, behavioral symptoms, and genetic mechanisms in ASD. This could help disentangle the heterogeneous relationships across these levels in ASD [3, 6]. Future studies should investigate the stability of this FC signature over time [74] and determine at what developmental stage it can be differentiated [75]. This requires large-scale longitudinal data, such as the Child Mind Institute Healthy Brain Network, aiming to recruit ~10,000 participants [76]. Detecting the signature in infants, especially high-risk neonates such as siblings of those diagnosed with ASD, could have implications for early detection and intervention [77]. Finally, investigating this high-risk ASD signature in comorbid [78] neurodevelopmental disorders may clarify the symptomatic [4], neurobiological [79, 80], and genetic [42, 81] overlap between these disorders and the autism spectrum.

### Conclusion

We report a functional connectivity signature associated with high risk of ASD that can be detected with high positive predictive value in independent data. Application of a targeted, high-confidence prediction model was able to identify functional connectivity alterations with high penetrance, evident in a small subset of individuals. This highlights the heterogeneity of the autism spectrum, decomposing some of the contribution from functional connectivity, which traditional neuroimaging machine learning studies fail to do by optimizing average accuracy. Decomposing the autism spectrum bit by bit in this manner may eventually help us understand the multitude of etiological pathways and their extension to the general population, offering avenues for further research on specific, high-risk ASD signatures.

## Materials and Methods

### Ethics, consent, and permissions

All imaging data used in this study were sampled from publicly available datasets. The inclusion of data in these samples was conditional on the approval of the respective local institutional review board and shared in a deidentified form according to the requirements identified by the Health Insurance Portability and Accountability Act. Written informed consent/assent was obtained for all participants. The use of these data for the analyses presented in this study was approved by the “Comité Mixte d'éthique en recherche regroupement neuroimagerie du Québec” (CMER RNQ), approval number 14–15-002.

### Sample

All data were sampled from the ABIDE 1 [30] and ABIDE 2 [72] dataset releases that contain imaging data for ASD patients and NTCs. We used the ABIDE 1 release as a discovery dataset and retained the ABIDE 2 release as an independent validation dataset.

**Figure 5:**
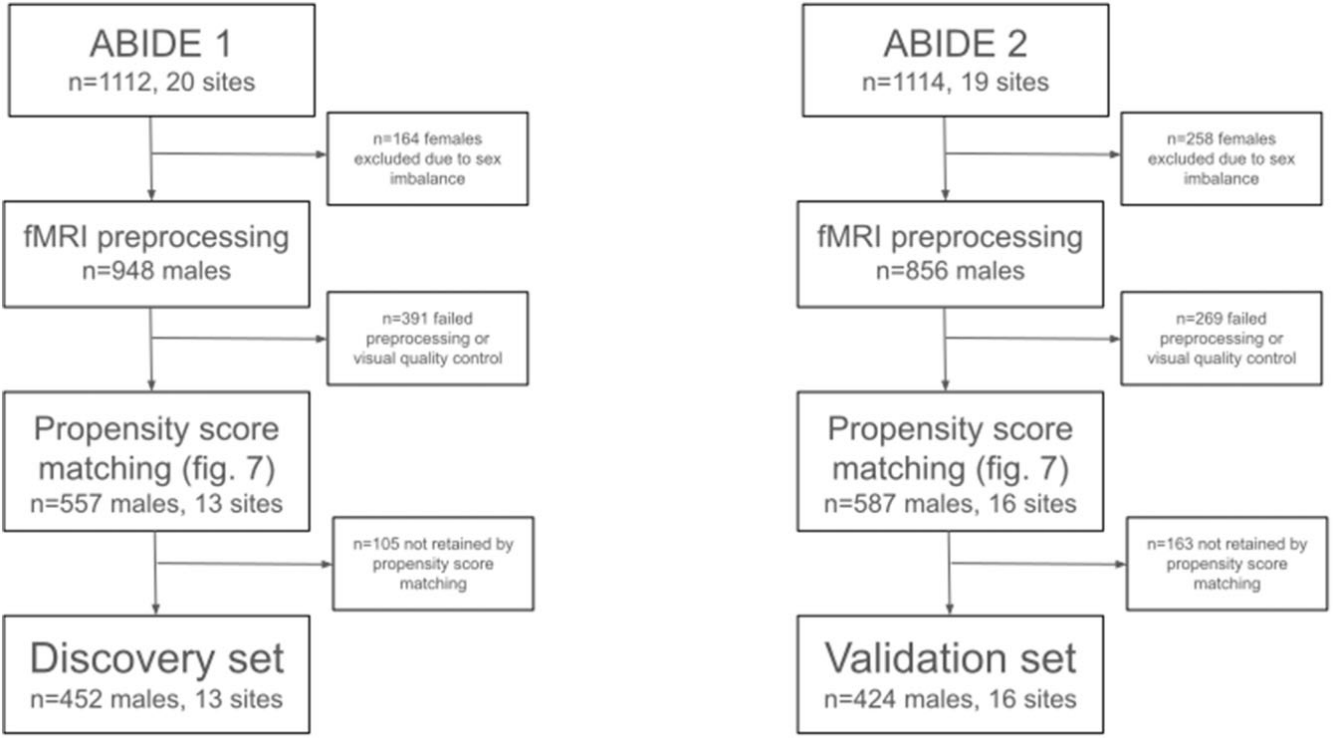
Flowchart showing how individuals were selected from the ABIDE 1 and 2 datasets.

**Figure 6:**
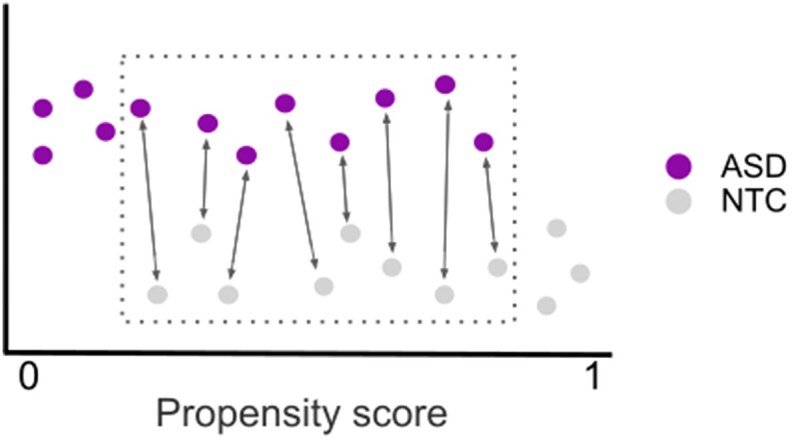
Propensity score matching schematic. First, propensity scores are estimated for each individual using selected covariates (age and head motion). We then used nearest-neighbor matching, whereby individuals are matched with the closest individual from the other group that falls within an acceptable range on the propensity score axis. Data points within the dotted area represent successful matches, while those outside are excluded from further analysis. For the current study, we used matching without replacement, which results in equal-sized groups. This procedure was applied separately for each data collection site.

The final discovery dataset consisted of 452 male individuals (age 16.42, 6.91 SD, 226 ASD) from 10 recording sites. From the complete ABIDE1 dataset of 1,112 individuals (age 17.04, 8.04 SD, 539 ASD) from 20 imaging sites, we excluded 164 female individuals due to strong sex imbalance. Of the remaining sample, 557 individuals from 10 imaging sites were successfully preprocessed and passed visual quality control (age 16.65, 6.75 SD, 272 ASD). See Fig. 5 for a flowchart of participant selection. In order to control for the effects of nuisance covariates in the data without removing variance due to the ASD diagnosis, we then matched ASD and NTC individuals on age and head motion within each imaging site by propensity score matching without replacement (Fig. 6) [82].

The validation dataset consisted of 424 male individuals (age 13.66, 5.25 SD, 212 ASD) from 16 imaging sites. From the complete ABIDE2 dataset of 1,114 individuals (age 14.86, 9.16 SD, 521 ASD) from 19 imaging sites, we excluded 258 female individuals due to the strong sex imbalance and to match the sample characteristics of the discovery sample. Of the remaining sample, 587 (age 13.94, 5.9 SD, 273 ASD) from 16 imaging sites were successfully preprocessed and passed visual quality control. In line with the sample selection of the discovery sample, we then matched ASD and NTC individuals on age and head motion within each imaging site using propensity score matching without replacement.

### Clinical diagnosis and severity estimates

The individuals from the ABIDE1 and ABIDE2 samples included in this study were diagnosed with ASD by expert clinicians based on either the ADOS [83–85] or the Autism Diagnostic Interview–Revised [86]. Using a published conversion table [87], we converted these to proxy ADOS calibrated severity scores (ADOS-CSS), which are less influenced by an individual’s age and other demographic confounds. Proxy ADOS-CSS scores could be computed for 221 individuals (190 ASD) in the discovery sample and 223 (207 ASD) in the validation sample, and they were strongly correlated with true ADOS-CSS scores in both (Pearson’s *r* = 0.90 and 0.94, respectively, both with *P* = 0.000).

### Imaging data preprocessing

Imaging data from individuals in both the discovery and independent validation sample underwent identical preprocessing through the NeuroImaging Analysis Kit (NIAK) [88] (version 1.1.3), the MINC toolkit [89] (version 1.9.15), Octave [90] (version 4.2.1), and Ubuntu [91] (version 16.04.2LTS), running inside a Singularity containerized environment [92] (version 2.6.1). Preprocessing of MRI data was executed in parallel on the Cedar supercomputer [93], using the Pipeline System for Octave and Matlab (PSOM) [94] (version 2.3.1). In short, functional time series were corrected for in-scanner head motion and registered to the MNI152 stereotaxic space [95]. Slow time drift signals were modeled on the continuous time series by a discrete cosine transformation and removed after censoring of time frames with excessive (>0.4 mm) head motion [96], together with nuisance covariates of the average white matter, cerebrospinal fluid signals, and the first principal components (accounting for 95% of variance) of the 6 degrees of freedom head motion estimates and their squares [97]. The preprocessed imaging data were visually quality controlled to ensure the quality of the data. Quality control (QC) was performed by a trained rater according to our in-lab standardized QC protocol [98] using a guided QC environment [99].

### Functional connectivity estimation

Seed-to-voxel FC was estimated for functional brain networks defined in the MIST_20 atlas [100]. The MIST_20 atlas represents 20 large, spatially distributed subcomponents of canonical FC networks. A large number of individuals were found to have incomplete coverage of the cerebellum, and so we excluded 2 networks that were part of the cerebellum. For each of the remaining 18 brain networks, the average within-network time series was correlated with the time series of all noncerebellar voxels using Pearson’s correlation.

### High-confidence prediction

In order to achieve a high specificity of ASD predictions, we limit predictions to cases where our model has a high level of confidence that an individual is not a neurotypical participant (NTC). We compute the confidence of the prediction by applying the TCP approach [36, 41]. TCP computes how “usual” (or conformal) the features of an unclassified individual (UCI) would be if we assumed an ASD or NTC label, compared to already classified individuals with these labels. That is, given an individual that we want to classify as either ASD or neurotypical, the conformal predictor asks, “How unusual would this individual be, if they were an individual with ASD?” and “How unusual would they be, if they were a neurotypical individual?” The predictor then answers each of these questions by comparing the individual to known individuals with ASD and neurotypical individuals, respectively. In this way, 2 conformality scores for each individual are computed, one for each of the 2 possible label classes. The predicted conformality score for each label then allows us to only make predictions when we have a high level of confidence in rejecting 1 label, that is, if an individual would be very “unusual” as an NTC participant (see Fig. 3). More technical introductory accounts of the conformal prediction logic can be found in Gammerman and Vovk [101] and Shafer and Vovk [102].

**Figure 7:**
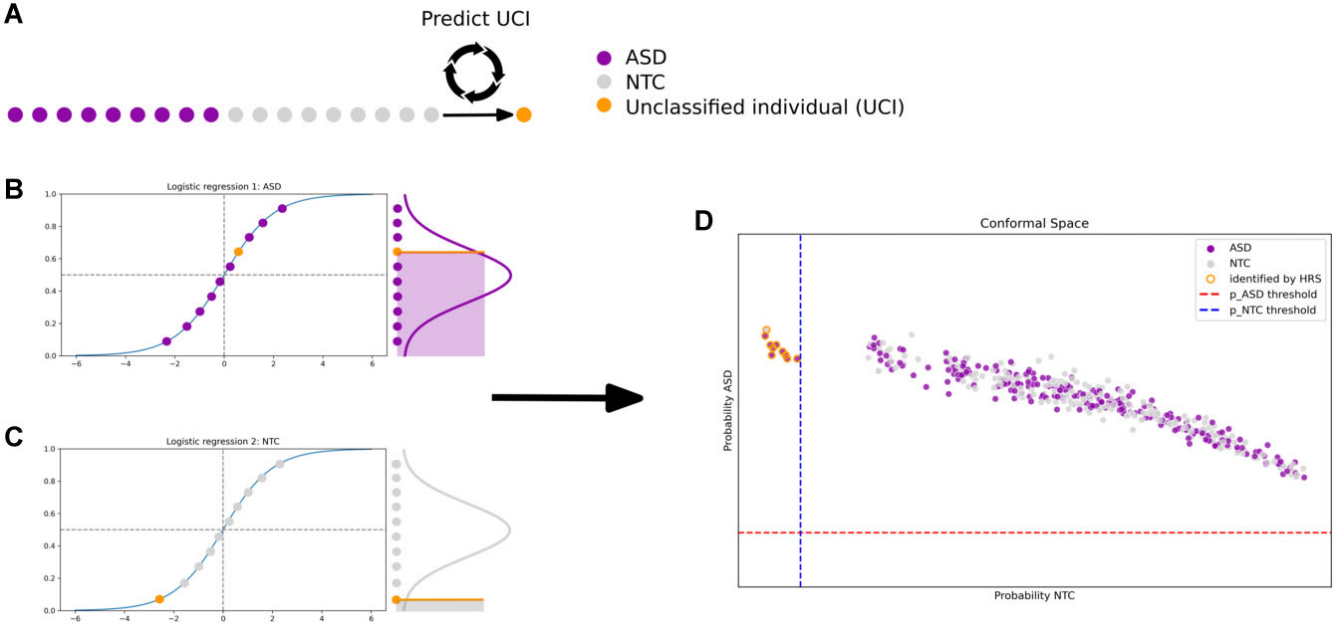
Schematic of transductive conformal prediction. (A) Circles represent individuals in the dataset, either ASD (purple) or NTC (gray). One individual from the sample at a time is designated the UCI (orange). Group-level nuisance regression and dimensionality reduction is conducted on the entire sample, including the UCI. The black circle represents that each individual in the dataset is designated the UCI in turn. (B) A first logistic regression is fitted to predict an ASD label. A scaling factor is used to increase specificity by minimizing false positives. (C) A second logistic regression is fitted to predict a label of NTC. The conformal scores are determined based on how unusual the UCI is compared to each group, calculated as the percentage of individuals known to have the assumed label and have an equal or lower predicted value than the UCI. The shaded areas in plots B and C visually indicate these individuals known to have the assumed label who also have a lower or equal predicted score than the UCI. (D) To limit ASD predictions to the most confident cases, predictions are only made if the ASD conformal score is >5% and NTC conformal score is <5%. This process is repeated for each UCI independently.

In contrast to an inductive classification approach, where a statistical model is first learned based on the properties of the reference set and then applied to new data, in a transductive classification, no model is learned and each new individual is classified directly and separately by comparing it to the properties of each class (ASD and NTC) in the reference set and choosing the class it most conforms to [103]. Each UCI therefore has to be treated in the exact same way to ensure the independence of each classification. See Fig. 7 for a schematic.

#### Regression of nuisance covariates

To account for potential confounding effects, we combine the UCI and the reference sample and use ordinary least squares regression to remove the group-level average connectivity and the linear effect of age and head motion from the network FC maps, retaining the residuals for further analysis.

#### Dimensionality reduction

Previous works have shown the capacity of FC subtypes to capture disease-related FC variability (e.g., Easson et al. [104]) and the utility of hierarchical ensemble methods for identifying neuroimaging-based subtypes [105]. We therefore identify the 5 subtypes of FC variability across both the UCI and the reference sample by hierarchical agglomerative clustering of spatially correlated, individual FC maps. For each individual, we then compute the spatial similarity with the average FC map of each of the 5 FC subtypes.

#### Estimation of conformality and classification

The individual conformality estimate for either clinical label (i.e., ASD or NTC) was then computed similarly to the previous work of Nouretdinov et al. [41]. In short, we first assumed an ASD label for each UCI and then fit a logistic regression to predict ASD for both the UCI and the reference sample, using the previously estimated similarity with FC subtypes as features. To reflect the fact that we wanted the model to make as few false-positive errors as possible, we weighed the predicted values of ASD individuals by a large scaling factor (w(ASD) = 10^16^). This forced the prediction model to only be concerned with the identification of ASD cases, with high specificity, at the expense of possible identification of NTC individuals. We computed the ASD conformal score for each UCI as the percentage of ASD individuals in the reference sample with a predicted value equal to or smaller than the one that was predicted for that UCI. In other words: if most ASD individuals had larger predicted values than the UCI, then the UCI did not conform to the ASD cohort and was an unusual ASD case, and thus the ASD conformal score would have been small due to the individual not “conforming” to the reference cohort of ASD individuals. An analogous process was then repeated to compute the NTC conformal score of the UCI.

We rejected a label (i.e., ASD or NTC) if the corresponding estimated conformal score was below a critical threshold of 5%. We predicted ASD with high confidence for only those individuals who had NTC conformal scores below the critical threshold and ASD conformal scores equal to or greater than the critical threshold.

### Performance assessment

**Figure 8:**
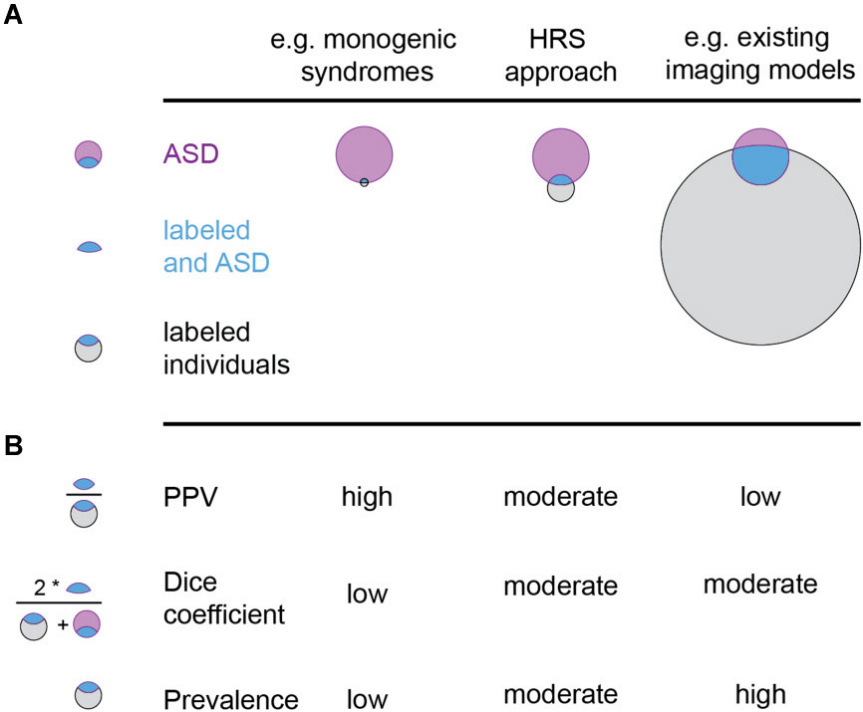
Schematic representation of properties of different ASD risk markers. (A) A set of individuals in the population is found to express the risk marker (gray) and is thus labeled. Among the set of individuals with ASD in the population (purple), some are also labeled by the risk marker (blue). Risk markers differ in the amount of labeled individuals from very few (left column) to very many (right column). (B) Different metrics exist to evaluate the performance of the risk marker. The ratio of ASD individuals among the labeled individuals (PPV) can be very high if only a very few individuals are labeled by the risk marker (e.g., in monogenic syndromes with high risk for ASD, left column). However, the degree of congruence of ASD and labeled individuals (Dice coefficient) would be very low, because of the large number of unlabeled ASD individuals. Conversely, a risk marker that labels many individuals may capture more ASD individuals and have a moderately higher Dice coefficient but would have a very low ratio of ASD to labeled individuals (PPV) and thus confer very low individual risk (e.g., existing imaging-based models, right column). The HRS approach presented here labels fewer individuals than current imaging models, but those individuals are more likely to have ASD, resulting in higher PPV and comparable Dice coefficients.

To assess the quality of the classification, we computed sensitivity, specificity, PPV, risk ratio (RR), odds ratio (OR), and the Sørensen–Dice coefficient. Detailed equations and explanations are provided in the supplementary materials. Briefly, PPV depends on the prevalence of ASD in the sample and estimates the individual probability of a true ASD diagnosis. If the model indicates any risk, the risk of ASD is higher for someone identified by the model than for someone not identified, measured by the RR. The OR is similar but does not depend on prevalence. The Sørensen–Dice coefficient evaluates the overlap between true ASD cases and model predictions, ranging from 0 (no overlap) to 1 (complete overlap). See Fig. 8 for a schematic of PPV and the Sørensen–Dice coefficient in relation to different ASD risk markers.

### Bootstrap estimation

We estimated the model performance of each brain network predictor through bootstrap subsampling of the discovery dataset. We drew 2 random bootstrap samples from the discovery dataset and assigned one to be the reference dataset and the other to be the prediction dataset. The ASD diagnosis of each individual in the prediction dataset was then separately predicted based on the individuals in the reference dataset, following preprocessing, feature extraction, and training as described above. We repeated this process 100 times for each brain network and computed the average performance metrics of each predictor across bootstraps. See, for example, Efron [106] regarding bootstrap predictor evaluation methods.

### Combination of correlated conformal predictions

To identify similarities of conformal predictions between the 18 functional brain networks, we computed the pairwise correlation of ASD nonconformity. We then used hierarchical agglomerative clustering to identify groups of networks with correlated ASD conformal score estimates. We selected a 7- and 2-cluster solution based on a visual inspection of the network-by-network correlation matrix.

Within each cluster of networks, conformal score estimates (i.e., probability estimates of nonconformity with each class label) were combined using the *P* value averaging methods of Vovk and Wang [107]. Specifically, we averaged over the *P* values that are associated within each network using the squared-mean merging function, which produces a valid aggregate *P* value from the combination of any finite number of potentially correlated individual *P* values. This requirement of validity is important in order to maintain the conformity properties when using these cluster-aggregated *P* values as inputs in a conformal predictor.

The aggregation of *P* values was observed to average over the information inherent in each of the contributing *P* values. As such, less informative network elements tended to decrease the explanatory power of the more informative elements. The overall effect was that the cluster nonconformity threshold tended to be conservative in identifying interesting observations, when compared to the same threshold value, applied to individual networks. In order to mitigate against this conservative effect, we used a more liberal threshold for cluster-aggregated *P* values than those used for individual networks. That is, we adjusted the critical nonconformal threshold to 0.2 from 0.05.

### Validation on the independent dataset

The HRS identified on the discovery sample was then validated on the independent validation sample. To do so, the ASD and NTC nonconformity estimate of each individual in the validation sample was computed by using the individuals of the discovery sample as the reference cohort. Each individual in the validation sample was predicted independently after group-level nuisance regression and dimensionality reduction with respect to the reference sample.

### Estimation of model performance in the general population

The discovery and validation sample had equal rates of ASD patients and NTC individuals (i.e., 1 ASD for each 1 NTC). The prevalence of ASD in the general population is, however, much lower (1 ASD for each 89 NTCs). Based on the estimated specificity and sensitivity of our model in the independent validation sample, we estimated the positive predictive value (*PPV_ASD_*) of the HRS in the general population.

## Supplementary Material

giaf091_Supplemental_File

giaf091_Authors_Response_To_Reviewer_Comments_Original_Submission

giaf091_Authors_Response_To_Reviewer_Comments_Revision_1

giaf091_Authors_Response_To_Reviewer_Comments_Revision_2

giaf091_GIGA-D-24-00438_original_submission

giaf091_GIGA-D-24-00438_Revision_1

giaf091_GIGA-D-24-00438_Revision_2

giaf091_GIGA-D-24-00438_Revision_3

giaf091_Reviewer_1_Report_Original_Submission Jing Sui -- 11/15/2024

giaf091_Reviewer_1_Report_Revision_1 Jing Sui -- 5/6/2025

giaf091_Reviewer_2_Report_Original_Submission Yuan Feng -- 12/14/2024

giaf091_Reviewer_2_Report_Revision_1 Yuan Feng -- 5/2/2025

## Data Availability

All data used in this article are available for download on Zenodo [108]. These data can be used to fully reproduce the analyses. Alternatively, figures can be reproduced using the precomputed results also available at the same link. The raw imaging data are publicly available from ABIDE 1 [30] and ABIDE 2 [72]. An archival snapshot of the code is available via Software Heritage [109]. The project was also registered on WorkflowHub [110]. DOME-ML (Data, Optimization, Model and Evaluation in Machine Learning) annotations are available in the DOME registry via accession v5y6w623hd [111].

## Additional Files

**Supplementary Fig. 1**. Predictions by the high-risk signature exceed the positive predictive value (PPV) of those by a simple baseline model. The distribution of PPV estimates across 100 bootstrap samples is denoted by violin plots for each model.

**Supplementary Fig. 2**. The impact of different levels of ASD prevalence in the data is shown for different metrics that are commonly used to evaluate prediction models. In balanced samples (black vertical line) that are commonly used to train models, traditional models (2 pink lines) that balance sensitivity and specificity achieve high accuracy. However, predictions by traditional models confer lower individual risk (PPV), particularly for low ASD prevalence, close to the baseline rate in the general population (gray lines). The current state-of-the-art PPV, obtained with 10 machine learning models combined, is shown in red. In the PPV figure, the HRS model and Traut et al. [26] lines are indistinguishable.

## Abbreviations

ABIDE: Autism Brain Imaging Data Exchange; ADOS: Autism Diagnostic Observation Schedule; ASD: autism spectrum diagnosis; CNV: copy number variant; CSS: calibrated severity score; DMN: default mode network; FC: functional connectivity; fMRI: functional magnetic resonance imaging; HRS: high-risk signature; NTC: neurotypical control; OR: odds ratio; PPV: positive predictive value; QC: quality control; ReHo: regional homogeneity; RR: risk ratio; SNP: single-nucleotide polymorphism; SVM: support vector machine; TCP: transductive conformal prediction; UCI: unclassified individual.

## Availability of Source Code and Requirements

Project name: High-precision machine learning identifies a reproducible functional connectivity signature of autism spectrum diagnosis in a subset of individuals

Project homepage: https://github.com/SIMEXP/autism-signature

Operating system(s): Platform independent

Programming language: Python, R

Other requirements: Jupyter notebook (used for supplementary analyses and figures)

License: MIT
