# Supplementary material for: High-precision machine learning identifies a reproducible functional connectivity signature of autism spectrum diagnosis in a subset of individuals
Source: Gigascience. 2025 Sep 3;14:giaf091. doi: 10.1093/gigascience/giaf091 (PMC12406215; doi:10.1093/gigascience/giaf091)
Supplement: giaf091_Supplemental_File [file giaf091_supplemental_file.pdf]

High-precision machine learning identifies a reproducible functional connectivity signature of autism spectrum diagnosis in a subset of individuals - supplementary materials

MATERIALS AND METHODS

Performance assessment

To assess the quality of the classification, we computed the sensitivity and specificity across the predicted individuals. The sensitivity of the classification:

$$sensitivity_{ASD} = \frac{TP}{ASD_{TRUE}}$$

reflects the ability of our model to correctly predict autism spectrum diagnosis (ASD) (*TP*) among those individuals who truly have ASD (ASD_{TRUE}). Incorrectly predicting an ASD diagnosis for an individual without the diagnosis is known as a false positive (*FP*) error. Our approach tried to minimize the false positive error. The specificity of the classification:

$$specificity_{ASD} = \frac{TN}{NTC_{TRUE}}$$

likewise reflects its ability to correctly not predict ASD (*TN*) among those individuals who truly do not have an ASD diagnosis (neurotypical controls, NTC) (NTC_{TRUE}). Incorrectly predicting an ASD individual as “not ASD” is known as a false negative (*FN*) error. The positive predictive value (*PPV*):

$$PPV_{ASD} = \frac{TP}{TP+FP}$$

is the ratio of correct ASD predictions (*TP*) among all ASD predictions made by our model. It thus reflects the risk of an individual classified as ASD by our model to truly have an ASD

diagnosis. Our approach aimed to maximize the positive predictive value. The *PPV* depends on the ratio of ASD_{TRUE} among all individuals in our sample. This ratio is known as the *prevalence* of ASD in the sample.

For an individual who was identified by the model as suspected ASD, the PPV_{ASD} provides an estimate of the individual probability of a true ASD diagnosis. If the model confers any risk, then the risk of ASD is larger for someone identified by the model than for someone not identified by the model. This measure is called the risk ratio (RR_{ASD}):

$$RR_{ASD} = \frac{TP/(TP+FP)}{TN/(TN+FN)}.$$

A similar metric that is independent of the prevalence of the disorder is the odds ratio (OR). The odds of a true ASD diagnosis for a selected individual is the ratio of the probability

$$p(ASD|selected) = \frac{TP}{TP+FP} = PPV_{ASD}$$

over the probability

$$p(not\ ASD|selected) = \frac{FP}{TP+FP}.$$

Both can be simplified to

$$Odds_{ASD} = \frac{TP}{FP}.$$

Analogous to the risk ratio, the odds ratio:

$$OR_{ASD} = \frac{TP/FP}{TN/FN}$$

then reflects the ratio of odds of an ASD diagnosis for selected individuals over the odds of ASD for unselected individuals.

For a model that conveys no information on the ASD diagnosis, the odds of a true ASD diagnosis are the same for individuals who are identified by the model and for those who are not identified (i.e., the OR is 1).

An ideal model would correctly classify all individuals with ASD. That is, the set of selected individuals and individuals with ASD would be exactly overlapping. In practice, models with high PPV (e.g., monogenic risk markers) tend to select only a very small subset of individuals (low sensitivity) and models with high sensitivity tend to incorrectly select many individuals without ASD (low specificity, see figure 5 of the main paper). We can thus use the overlap between individuals with ASD and selected individuals to determine how close the model is to an optimal tradeoff between sensitivity and specificity. The Sørensen–Dice coefficient:

$$Dice = \frac{2*TP}{ASD_{TRUE} + (TP + FP)}$$

measures the ratio of correctly selected individuals over the sum of individuals with ASD (ASDTrue) and all selected individuals. It thus ranges between 0 (if the two sets are not overlapping) and 1 (if the two sets are completely overlapping).

RESULTS

High risk signature tends to identify individuals with severe symptoms

We next investigated the symptom characteristics of the individuals who were identified by the high risk signature (HRS) model. To that end, we reported their ADOS severity measures and compared them to those of unselected individuals from the same clinical category. Because only 10 individuals were identified by the HRS model, these results are exploratory and we limited

ourselves to reporting only descriptive measures. Calibrated ADOS severity scores (ADOS-CSS) would have been the preferred measure to interpret symptom severity because of their standardised range (from 1: least severe symptoms to 10: most severe symptoms), and because of their comparability across ADOS modules and across different ages. However, ADOS-CSS were only available for 3 identified individuals. Using a previously published technique we therefore computed proxy ADOS-CSS based on the available data. We reported these closely approximated ($r = 0.94$) proxy ADOS-CSS together with the ADOS raw total scores.

The median of proxy ADOS-CSS was higher among the nine identified individuals with ASD (median = 9, interquartile range = 4 - 9) than among the remaining individuals with ASD who were not identified by the HRS model (median = 6, interquartile range = 5 - 8). The single NTC individual identified by the model had a higher proxy ADOS-CSS of 3 than the remaining NTC individuals who were not identified by the HRS model (median = 1, interquartile range = 1 - 1). The same comparison using raw ADOS total scores revealed an analogous finding: the median of raw ADOS total scores was higher among the nine identified ASD individuals (median = 15, interquartile range = 13 - 17) than among the remaining unidentified ASD individuals (median = 10, interquartile range = 8 - 13.25). Accordingly, the single identified NTC individual had a higher raw ADOS total score of 7 than the remaining unidentified NTC individuals (median = 1, interquartile range = 0 - 2). Figure 3 (main paper) shows both the proxy ADOS-CSS and the raw ADOS total scores of the identified individuals compared to those of unidentified individuals with the same diagnostic class. It can be seen that there is an overlap in the distribution of scores for individuals with ASD who were and were not selected by the model. Our exploratory findings thus indicate that the identified individuals tended to show particularly severe symptoms

for their diagnostic class but, importantly, the model does not only identify those with severe symptoms.

Medication usage

Of the 10 ASD individuals identified by the model, 4 were taking medication (40%). Of the 203 ASD not identified, 53 were taking medication (26.11%). Given the small *N* we used Fisher's exact test to test for significance. The results of the Fisher's exact test (p = .254) do not indicate a significant association between medication usage and group membership, although this post-hoc test clearly has very small statistical power given the small size of the group. Code for this analysis can be found at https://github.com/surchs/ASD_high_risk_endophenotype_code_supplement.

Supplementary figures

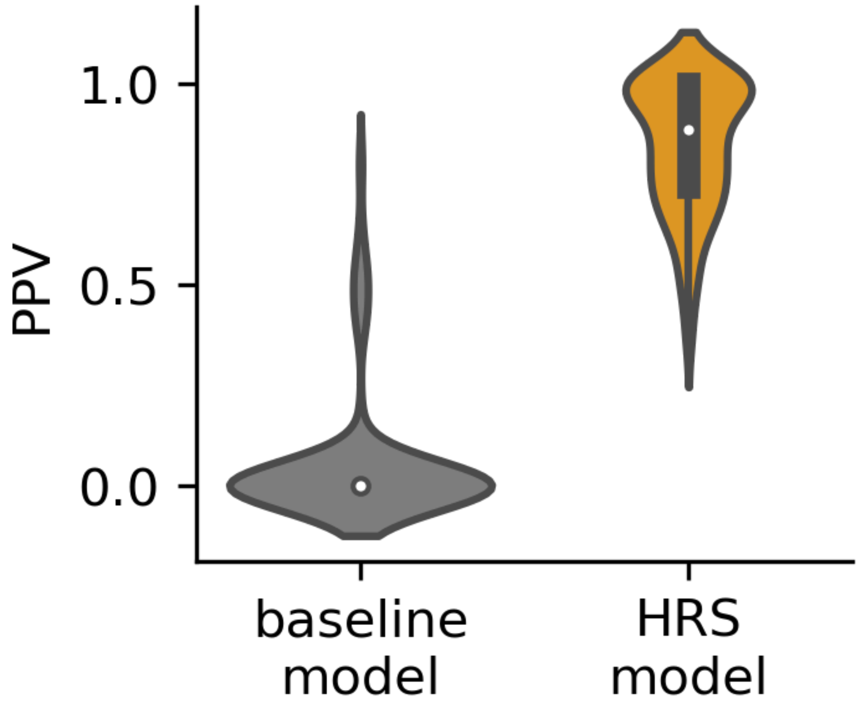

Figure supplement 1. Predictions by the high risk signature exceed the positive predictive value (PPV) of those by a simple baseline model. The distribution of PPV estimates across 100 bootstrap samples is denoted by violin plots for each model.

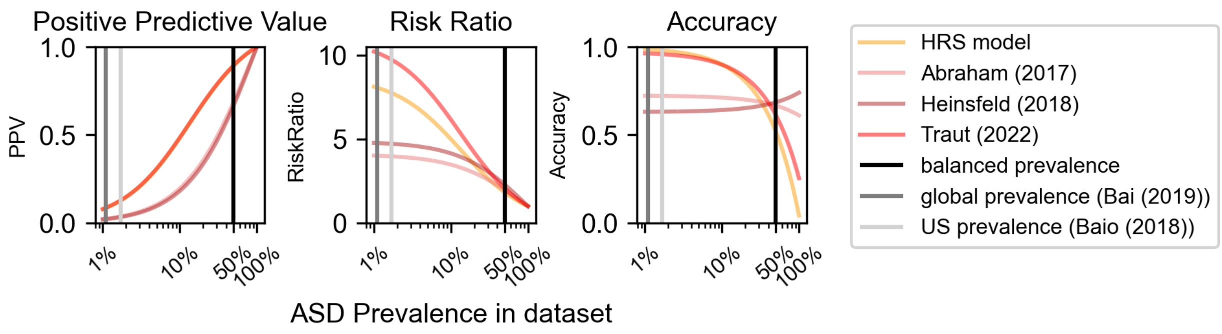

Figure supplement 2. The impact of different levels of ASD prevalence in the data are shown for different metrics that are commonly used to evaluate prediction models. In balanced samples (black vertical line) that are commonly used to train models, traditional models (two pink lines) that balance sensitivity and specificity achieve high accuracy. However, predictions by traditional models confer lower individual risk (PPV), particularly for low ASD prevalence, close to the baseline rate in the general population (grey lines). The current state-of-the-art PPV, obtained with 10 machine learning models combined, is shown in red. In the PPV figure, the HRS model and Traut (2022) lines are indistinguishable.
