# Supplementary material for: High-precision machine learning identifies a reproducible functional connectivity signature of autism spectrum diagnosis in a subset of individuals
Source: Gigascience. 2025 Sep 3;14:giaf091. doi: 10.1093/gigascience/giaf091 (PMC12406215; doi:10.1093/gigascience/giaf091)
Supplement: giaf091_GIGA-D-24-00438_Revision_2 [file giaf091_giga-d-24-00438_revision_2.pdf]

GigaScience

High-precision machine learning identifies a reproducible functional connectivity signature of autism spectrum diagnosis in a subset of individuals

--Manuscript Draft--

**Manuscript Number:**	GIGA-D-24-00438R2
**Full Title:**	High-precision machine learning identifies a reproducible functional connectivity signature of autism spectrum diagnosis in a subset of individuals
**Article Type:**	Research

Funding Information:

Azrieli Foundation (3388)	Dr Sebastian Urchs
Australian Research Council (DE170101134 and DP180101192)	Dr Hien Duy Nguyen
Brain Canada Multi Investigator Research Initiative	Dr Sebastian Urchs<br>Dr Clara Moreau
Consortium canadien en neurodégénérescence associée au vieillissement	Dr Clara Moreau
Canadian Open Neuroscience Platform	Dr Sebastian Urchs
Centre de recherche de l'Institut universitaire de geriatrie de Montreal	Dr Angela Tam
Courtois Foundation	Dr Sebastian Urchs<br>Dr Clara Moreau<br>Dr Angela Tam
Institut de Valorisation des Données	Dr Natasha Clarke
Healthy Brains, Healthy Lives	Dr Clara Moreau
Fonds de Recherche du Québec - Santé	Professor Lune Bellec

Abstract:

Background
Discovery of predictive biomarkers is essential for understanding the neurobiological underpinnings of autism spectrum diagnosis (ASD) and improving identification. Resting-state functional connectivity analyses of individuals with ASD have established sensitivity of brain connectivity at the group level. However, the extensive heterogeneity in ASD limits the translation of these findings into reliable individual-level biomarkers. We analysed the Autism Brain Imaging Data Exchange (ABIDE) 1 and 2 datasets, calculating Pearson's correlation-based functional connectivity across 18 brain networks. Using transductive conformal prediction, a machine learning approach that assigns confidence scores to predictions based on conformality to known classes, we classified individuals with ASD and neurotypical controls.
Results
By combining predictors into an ensemble using hierarchical agglomerative clustering, we identified a signature that confers a more than 7-fold increase in individual risk of ASD diagnosis, yet is still identified in an estimated 1 in 200 individuals in the general population. The individual risk conferred by the model is increased 4-fold over that of previously published imaging models, and outperforms the current state of the art in precision for ASD classification. The high risk signature was characterised by underconnectivity of transmodal brain networks, including the frontoparietal and basal ganglia network, and subcomponents of the limbic and default mode networks.
Conclusions
A highly targeted prediction model can identify a subset of functional connectivity alterations that confer high-risk for ASD at the individual level, which may be masked by traditional machine learning models due to ASD heterogeneity. Results could help disentangle the multitude of etiological pathways and behavioural symptoms that challenge our understanding of ASD by focusing on highly penetrant connectivity signatures.

Corresponding Author: Natasha Clarke
Institut Universitaire de Geriatrie de Montreal

Powered by Editorial Manager® and ProduXion Manager® from Aries Systems Corporation

	Montreal, CANADA
**Corresponding Author Secondary Information:**	
**Corresponding Author's Institution:**	Institut Universitaire de Geriatrie de Montreal
**Corresponding Author's Secondary Institution:**	
**First Author:**	Natasha Clarke
**First Author Secondary Information:**	
**Order of Authors:**	Natasha Clarke
	Sebastian Urchs
	Hien Duy Nguyen
	Clara Moreau
	Christian Dansereau
	Angela Tam
	Alan C. Evans
	Lune Bellec
**Order of Authors Secondary Information:**	
**Response to Reviewers:**	Dear Dr Zauner,<br><br>We are grateful for you and the reviewers for reviewing our manuscript and accepting it for<br>publication (in principle), thank you for your time. We have addressed the few outstanding<br>comments from the reviewers in the paper, and below. Please note we have also updated the title to better describe the paper, and remove the mention of "risk".<br><br>Best,<br>Dr Natasha Clarke<br>On behalf of all authors<br><br>Reviewer 1<br>R1.1. The authors have addressed most of the concerns quite well, but the existing literature review need to be improved, some of the following relavant articles on ASD subtypes classification with ensemble learning, and FC prediction were missed, which should be included in the manuscript.<br>1. S Qi, R Morris, et al. 2020. Common and unique multimodal covarying patterns in autism spectrum disorder subtypes. Molecular autism 11, 1-15<br>2. N Luo, J Sui, et al. 2020. Age-related structural and functional variations in 5,967 individuals across the adult lifespan. Human brain mapping 41 (7), 1725-1737<br>3. D Yao, VD Calhoun et al. 2018. An ensemble learning system for a 4-way classification of Alzheimer's disease and mild cognitive impairment. Journal of neuroscience methods 302, 75-81<br><br>We thank the reviewer for providing these references which we missed, and have added<br>them to the relevant sections of the manuscript.<br><br>Reviewer 2<br>R2.6. I see that the term "different modalities" wasn't used in the manuscript. But my original point was about how "transmodal" is used. In neuroscience, "transmodal"<br>refers to high-level brain regions that integrate information across functions—not to data<br>types or network relationships. Since the paper discusses between-network connectivity

	and calls them “transmodal networks,” it might confuse readers into thinking this is about<br>multimodal data. A short explanation would help avoid that.<br><br>We appreciate the clarification and have added an explanation in the discussion, line 341-345.<br><br>R2.8 Just to clarify—I didn’t mean you didn’t do nuisance regression, or that it wasn’t described in the Methods. My point was that this step is standard preprocessing, and too much emphasis on it in the Results can distract from your main findings. A brief mention is fine, but long explanations in the Results aren’t really necessary.<br><br>We thank the reviewer for the clarification. We have amended the section “Conformal prediction not driven by nuisance covariates” so that it is shorter.
**Additional Information:**	
**Question**	**Response**
Are you submitting this manuscript to a special series or article collection?	No
**Experimental design and statistics**<br><br>Full details of the experimental design and statistical methods used should be given in the Methods section, as detailed in our Minimum Standards Reporting Checklist. Information essential to interpreting the data presented should be made available in the figure legends.<br><br>Have you included all the information requested in your manuscript?	Yes
**Resources**<br><br>A description of all resources used, including antibodies, cell lines, animals and software tools, with enough information to allow them to be uniquely identified, should be included in the Methods section. Authors are strongly encouraged to cite Research Resource Identifiers (RRIDs) for antibodies, model organisms and tools, where possible.<br><br>Have you included the information requested as detailed in our Minimum Standards Reporting Checklist?	Yes
**Availability of data and materials**	Yes

All datasets and code on which the conclusions of the paper rely must be either included in your submission or deposited in publicly available repositories (where available and ethically appropriate), referencing such data using a unique identifier in the references and in the "Availability of Data and Materials" section of your manuscript.<br><br>Have you have met the above requirement as detailed in our Minimum Standards Reporting Checklist?	

Powered by Editorial Manager® and ProduXion Manager® from Aries Systems Corporation

Manuscript

Click here to access/download;Manuscript;highlighted_gigascience_High_Ris

High-precision machine learning identifies a reproducible functional connectivity signature of autism spectrum diagnosis in a subset of individuals

Authors: Natasha Clarke[1,2], Sebastian Urchs[1,3], Hien Duy Nguyen[4,5], Clara Moreau[1,6], Christian Dansereau[1], Angela Tam[1], Alan C. Evans[3], Lune Bellec[1,2]

Corresponding author: Natasha Clarke (natasha.clarke@criugm.qc.ca)

Other author email addresses: Sebastian Urchs (sebastian.urchs@gmail.com), Hien Duy Nguyen (h.nguyen7@uq.edu.au), Clara Moreau (claramoreau9@gmail.com), Christian Dansereau (christiandansereau@gmail.com), Angela Tam (angela.tam08@gmail.com), Alan C. Evans (alan.evans@mcgill.ca), Lune Bellec (lune.bellec@umontreal.ca)

Author affiliations:

1 Centre de Recherche de l'Institut Universitaire de Gériatrie de Montréal; QC H3W 1W5, Montréal, Canada.

2 Département de Psychologie, Université de Montréal; QC H2V 2S9, Montréal, Canada.

3 Montreal Neurological Institute and Hospital, McGill University; QC H3A 2B4, Montreal, Canada.

4 School of Computing, Engineering and Mathematical Sciences, La Trobe University; VIC 3086, Bundoora, Australia.

5 Institute of Mathematics for Industry, Kyushu University; Nishi-ku Fukuoka 819-0395, Japan.

6 Sainte Justine Research Center,Université de Montréal; QC H3T 1C5, Montréal, Canada.

Keywords: resting-state functional connectivity; autism spectrum diagnosis; transductive conformal prediction

Abstract

Background

Discovery of predictive biomarkers is essential for understanding the neurobiological underpinnings of autism spectrum diagnosis (ASD) and improving identification. Resting-state functional connectivity analyses of individuals with ASD have established sensitivity of brain connectivity at the group level. However, the extensive heterogeneity in ASD limits the translation of these findings into reliable individual-level biomarkers. We analysed the Autism Brain Imaging Data Exchange (ABIDE) 1 and 2 datasets, calculating Pearson's correlation-based functional connectivity across 18 brain networks. Using transductive conformal prediction, a machine learning approach that assigns confidence scores to predictions based on conformality to known classes, we classified individuals with ASD and neurotypical controls.

Results

By combining predictors into an ensemble using hierarchical agglomerative clustering, we identified a signature that confers a more than 7-fold increase in individual risk of ASD diagnosis, yet is still identified in an estimated 1 in 200 individuals in the general population. The individual risk conferred by the model is increased 4-fold over that of previously published imaging models, and outperforms the current state of the art in precision for ASD classification. The high risk signature was characterised by underconnectivity of transmodal brain networks, including the frontoparietal and basal ganglia network, and subcomponents of the limbic and default mode networks.

Conclusions

A highly targeted prediction model can identify a subset of functional connectivity alterations that confer high-risk for ASD at the individual level, which may be masked by traditional machine learning models due to ASD heterogeneity. Results could help disentangle the multitude of etiological pathways and behavioural symptoms that challenge our understanding of ASD by focusing on highly penetrant connectivity signatures.

INTRODUCTION

Autism spectrum diagnosis (ASD) is a complex neurodevelopmental condition diagnosed in approximately 1% of the general population [1], characterised by impairments in social interaction and repetitive behaviour [2]. ASD has been linked to changes in brain structure and function, and genetics, and is highly heritable, with an estimated heritability of 80% [1]. Despite the high heritability there is wide heterogeneity in both symptoms and genetics [3] and extensive overlap with other neurodevelopmental disorders such as attention deficit hyperactivity disorder and schizophrenia [4–6].

Discovery of predictive biomarkers is a fundamental aim in clinical neuroscience, and may help decompose the marked heterogeneity in ASD. Biomarkers are critical for unravelling the neurobiological mechanisms underlying ASD, finding novel treatment targets, and identifying individuals who may benefit from these interventions [7]. An ideal biomarker with the potential to guide clinical decision making at the individual level should combine two criteria: firstly, they should have high penetrance, conferring substantially increased ASD risk above the baseline for an individual with unknown ASD status. In machine learning, which offers valuable techniques for biomarker identification, this can be estimated using the positive predictive value (PPV). Secondly, biomarkers should have a high enough prevalence in the population to enable investigation in large cohort studies.

To date, most progress in biomarker detection for ASD has come from the field of genetics. “Genetics-first” studies have identified rare mutations such as copy number variants (CNVs) [8] - deletions or duplications of DNA segments that have large effects. However, applications of CNVs as a biomarker are limited by their low prevalence, typically occurring in fewer than 0.01% of individuals [9]. Conversely, common genetic variants such as single-nucleotide polymorphisms (SNPs) are found in more than 5% of the general population but have very low penetrance, conferring only a slight increase in ASD risk. The lack of a genetic mutation that demonstrates moderate prevalence and penetrance, despite the high heritability observed in ASD, has been termed the "missing heritability" gap [10,11]. This suggests the need for alternative biomarkers.

Resting-state functional connectivity (FC), measured by functional magnetic resonance imaging (fMRI), is sensitive to brain organisation in ASD [12,13] and may offer another avenue to identify high-risk markers more common in the general population. MRI is non-invasive, widely available, and FC is task-free, making it suitable for clinical populations. Many studies have used machine learning to detect predictive FC signatures in ASD, using a variety of FC metrics. Pearson's correlation coefficient between the timeseries of two regions of interest, determined using an atlas, seed-based, or data-driven approach such as independent component analysis [14], has been used as input to different machine learning models, revealing disruption to distributed networks in ASD. Common algorithms include support vector machines (SVM), with classification accuracies reported of around 67% and 79% [15–17]. In a direct comparison of SVM, random forest and a neural network on the same data, a neural network slightly out-performed both SVM and random forest, at 70% accuracy [18], and in general deep learning classification approaches for ASD likely outperform single layer algorithms [19]. At a local scale, regional homogeneity (ReHo), which measures FC between a voxel or region and its nearest neighbors, has shown comparable results to Pearson correlation [20,21]. Compared to these

static FC techniques, dynamic FC uses sliding windows to analyse how FC fluctuates over time. These features have been found to out-perform static FC in ASD classification, combined with a SVM, logistic regression [22,23] or ensemble classifier [24]. Here we focus on the common Pearson correlation FC, which captures both short and long range connectivities, and compared to dynamic FC is less computationally expensive and more easily interpretable.

Previous studies have faced significant challenges that impede the identification of reliable ASD biomarkers. Collection of MRI data is costly and time consuming, particularly problematic for machine learning studies which can overfit to noise in the training data. Indeed, accuracies increase as samples decrease, indicating bias [25]. Overfitting also increases with the ratio of features to samples, a problem for studies using high-dimensional fMRI data. The ability of a model to generalise, for example to data collected at other sites, is key for robust biomarkers, but many studies do not report generalisation to an independent sample. Even for models that perform well in cross validation, generalisation to completely unseen data leads to a drop in performance [26]. Thus although initial small, single-site studies showed good accuracy for ASD prediction, performance in large, multicenter cohorts has been lower, likely due to a combination of inflated performance estimates on the smaller samples [27] and clinical heterogeneity of ASD [28,29].

Heterogeneity in ASD arises from multiple sources, including behavioural symptoms, cognitive skills, genetics and brain alterations, giving rise to distinct subtypes. Research using CNVs has helped shed light on this heterogeneity since CNV-related brain alterations exhibit “mirror effects” on brain connectivity, with deletions and duplications affecting the same imaging measures in opposite directions [6]. This phenomenon may give rise to subgroup formation within idiopathic ASD cohorts, contributing to symptom heterogeneity. Clinically, distinction of subtypes has been challenging, instead leading to a focus on ASD as a spectrum [2]. Large scale studies of brain alterations in ASD, supported

by data-sharing initiatives such as the Autism Brain Imaging Data Exchange (ABIDE) [30], have been able to decompose this source of heterogeneity to reveal subtypes that map better to a continuous spectrum that discrete categories [31–33], suggesting promise for reliable FC biomarkers. FC patterns associated with such subtypes reveal idiosyncratic profiles that only exist in subsets of people with ASD [31,34]. Crucially, machine learning studies that collapse results across heterogeneous samples likely obscure these more predictive signatures, hampering the penetrance potential of traditional imaging biomarkers.

Additionally, typical machine learning studies use a similar number of control and ASD participants to train and evaluate their models, which does not accurately reflect the risk of ASD in the general population, where only 1 in 90 people has ASD. Even with high prediction accuracy, this translates to low PPVs of around 2.4% to 2.2% [15,18], not much higher than the baseline risk of 1-2% for ASD and comparable to common genetic mutations. A recent ensemble predictor from an ASD biomarker challenge reframed ASD classification to make a confirmatory diagnosis, by enforcing a low false positive rate and thus high specificity [26]. As the prevalence of ASD in the general population is low, high model specificity is important to achieve a high PPV, and so this approach resulted in a PPV of 8.6% in an estimated general population sample. However, this impressive result was achieved through a complex public prediction challenge, in which the top 10 of 146 submissions were combined into an ensemble predictor, making it challenging to apply elsewhere.

In this study we aim to identify a "brain-first" imaging signature that is more penetrant than existing imaging markers and common genetic variants, but with a relatively higher prevalence. To achieve this we reframe the traditional prediction problem from optimising the prediction accuracy across all individuals with ASD, to instead optimising the PPV, by focusing on individuals who we can predict with a high degree of confidence. To assess the degree of confidence in our predictions, we use

a rigorous statistical framework designed for this purpose called transductive conformal prediction (TCP) [35,36]. TCP explicitly computes the confidence in the clinical label predicted for each individual, and uses these estimates to limit predictions to individuals for whom there is a very high level of confidence. Since it is transductive, it uses both training and test data to predict individual examples, rather than building a general model. This allows it to adapt to the distribution of the data, reducing overfitting and improving robustness to heterogeneity, particularly useful for disorders like ASD, where generalisability is a challenge. Conformal prediction approaches are relatively underused [37], but have been applied in clinical research to give a reliable measure of prediction uncertainty in drug discovery [37], tumour biopsy [38] and conversion to dementia [39], and with neuroimaging data in stroke risk [40] and clinical depression [41], but to our knowledge have not been applied in ASD. We use a large discovery sample to identify the potential high risk signature, and validate it in a large replication sample, including estimating its prevalence and PPV in the general population. Finally, we report the connectivity and symptom profiles of individuals flagged by the signature. We hypothesise that by limiting predictions to the most confident cases, we will identify subsets of ASD individuals who share very predictive, high risk FC signatures. We further hypothesise that the FC of different brain networks may give rise to distinct high risk FC signatures.

RESULTS

Individual networks do not predict ASD with high PPV

We first evaluated the PPV of conformal ASD diagnosis predictions made with high confidence, based on the FC of each of the 18 brain networks (i.e., their FC was very atypical for NTC, with a conformal score < 5%, and not very atypical for ASD, with a conformal score > 5%) . To do so, we computed the median PPV of high confidence conformal predictions for each brain network across 100 bootstrap

samples (bootstrap PPV) of the discovery data. The bootstrap PPV of high confidence conformal ASD diagnosis predictions ranged from 58% (orbitofrontal network) to 66% (default mode network) and was 62% on average across all networks. That is, among the individuals predicted with high confidence to have an ASD diagnosis, 62% on average did have an ASD diagnosis. As expected, the predictions were made with high specificity (91% on average across all networks) and low sensitivity (15% across all networks). That is, on average, 91% of NTC individuals were correctly not predicted to have an ASD diagnosis, and 15% of ASD individuals were correctly predicted to have an ASD diagnosis. Figure 1 shows an overview of the bootstrap PPV across networks. We thus showed that high confidence predictions of ASD diagnosis made by individual brain networks did not lead to predictions with high PPV.

Figure 1. **Combining network predictors with correlated conformal scores results in higher prediction performance.** Figure shows the process of combining network predictors with correlated conformal scores to enhance the prediction performance for ASD. Left column = individual network models, middle column = combined models, right column = ensemble models. Individual networks (left column) were first clustered into combined predictors based on correlated conformal scores, using hierarchical agglomerative clustering of pairwise correlations of non-conformity scores (**B**, middle). Seven clusters were identified based on visual inspection of the correlation matrix, representing large scale functional networks (**A**, middle). Networks with correlated conformal predictions were further clustered into two large ensemble predictors (**B**, right), that combined predominantly unimodal (blue) and transmodal (orange) brain networks respectively (**A**, right). The PPVs associated with conformal predictions for each model are shown in the bottom row (**C**). They are lowest for the individual networks, and increase across combined and ensemble models. Predictions of the ensemble of more

transmodal networks (orange) gave rise to a high risk signature that predicted ASD with high positive predictive value (**C**, right).

Functionally similar brain networks predict correlated conformal scores

We investigated whether groups of brain networks existed that give rise to similar conformal predictions of ASD diagnosis and could be combined to achieve more accurate group predictions. We computed correlations between ASD conformal scores from individual brain network predictors and applied hierarchical agglomerative clustering, resulting in seven groups: group 1 was a single network group of the frontoparietal network; group 2 combined limbic and temporal networks (orbitofrontal cortex, inferior temporal sulcus, lateral default mode network (DMN), and amygdala-hippocampal complex); group 3 was a single network group containing the basal ganglia network; group 4 combined sub-components of the DMN (anterior-, and posterior-medial DMN, and perigenual anterior cingulate and ventromedial prefrontal cortex); group 5 combined unimodal sensory networks (ventral, and dorsal somatomotor network, and auditory network); group 6 combined attention networks (medial ventral, and lateral ventral attention network, and frontoparietal task control network); group 7 combined visual networks (medial-, lateral-, and downstream visual network). We thus show that functionally similar brain networks tended to give rise to correlated conformal predictions of ASD diagnosis.

We combined conformal scores from brain networks within each group to generate high confidence ASD predictions, evaluated over 100 bootstrap samples (see Methods for details). The median across bootstrap samples was used, as the distribution of PPV values was not normally distributed (see figure S1). The average bootstrap PPV across all groups was 64.4%, with high specificity (89%) and low sensitivity (18.4%). Group PPVs were similar to the average PPV of individual networks within them (group 2: 70% vs 61.5%; group 4: 67.3% vs 64.3%; group 5: 64.4%

vs 63.1%; group 6: 58.8% vs 61.7%; group 7: 62% vs 60.9%). Single network groups (group 1 and 3) had adjusted PPVs (group 1: 65.1% vs 64.1%; group 3: 61.4% vs 61.1%) (see table 1). Thus, groups of brain networks with correlated conformal scores predicted ASD with only marginally higher PPV than individual networks.

Ensemble of transmodal networks forms high risk ASD signature

We further combined brain networks with correlated conformal scores into two large ensemble predictors. Ensemble 1 included nine more transmodal networks from groups 1-4 (frontoparietal, limbic, basal ganglia, DMN), and ensemble 2 included nine more unimodal networks remaining from groups 5-7 (sensorimotor, attention, visual). Predictions were evaluated across 100 bootstrap samples. Ensemble 1 had a PPV of 83.4%, higher than its group predictors' average (62.7%). Ensemble 2 had a PPV of 67.2%, also higher than its group predictors' average (63%) (see table 1). Ensemble 1 showed higher specificity (99%) and lower sensitivity (5%) compared to Ensemble 2 (specificity 97%, sensitivity 7.5%). Combining all networks into a whole-brain model did not improve PPV (76.6%). We thus demonstrated that combining correlated network predictions into ensemble predictors (specifically, ensemble 1) produced a robust high risk signature (HRS) for ASD diagnosis, and chose to further investigate ensemble 1's high PPV signature in the independent replication dataset.

High risk ASD signature generalises to independent data

We assessed the generalizability of the HRS in an independent replication sample by computing conformal scores for each individual relative to the discovery sample. The HRS identified 10 individuals from 6 imaging sites in the replication sample, of whom 9 had an ASD diagnosis. The PPV of the HRS was 90% in the replication sample, similar to the discovery sample's bootstrap PPV of 83.4%. Specificity (99.5%) and sensitivity (4.2%) were also consistent with the discovery sample (99%

and 5%, respectively). Ensemble 2 showed similar results, with a PPV of 62.5% (discovery: 67.2%), specificity of 95.8% (discovery: 97.0%), and sensitivity of 7.1% (discovery: 7.5%). Thus, the high-risk ASD signature demonstrated similar predictive performance in an independent validation dataset.

High risk ASD signature translates to 7-fold risk increase in general population

The discovery and replication samples were balanced with equal numbers of individuals with ASD and NTC (50% prevalence) for model training and evaluation. However, in an unselected population, ASD prevalence is estimated to be 1.11% (1 in 90). The HRS identified 4.2% of individuals with ASD (sensitivity) and had a 0.5% false positive rate (1 - specificity). To estimate HRS performance in an unselected population, we calculated expected accuracy for an ASD prevalence of 1.11%. The HRS correctly identified 0.046% of the population (4.2% sensitivity × 1.11% individuals with ASD) and incorrectly identified 0.49% (0.5% false positive rate × 98.89% individuals without ASD or with NTC), resulting in a PPV of 9.2% (using unrounded values). Thus, an individual identified by the HRS had an 9.2% risk of ASD or a 7.8-fold increase over the baseline risk.

High risk signature characterised by underconnectivity

To identify the FC pattern of the individuals detected by the HRS model, we investigated the average residual connectivity maps of the identified individuals for the nine brain networks contributing to the HRS. Figure 2b shows the average residual connectivity maps of the nine networks, which are characterised by pervasive underconnectivity with respect to the rest of the discovery sample. We thus show that the FC signatures of individuals identified by the HRS model were characterised by wide-spread underconnectivity of the nine involved brain networks with respect to the sample average.

Figure 2. **The high risk signature tends to identify individuals with severe symptoms, and pervasive underconnectivity. A)** Individuals identified by the high risk signature (circles with orange outline) have high proxy calibrated ADOS severity scores (left plot) and high raw ADOS total scores (right plot) compared to the average of their respective diagnostic category. **B)** The identified individuals share a pattern of distributed below average functional connectivity of the nine networks driving the high risk signature (the networks are denoted by name and coloured outline on their respective connectivity maps).

Conformal prediction not driven by nuisance covariates

For all network and ensemble predictors, ASD conformal scores showed no significant correlations with age or head motion, with confidence intervals including zero (figure 3). Thus, ASD conformal scores were not substantially influenced by nuisance variables. Medication use also did not differ between ASD individuals identified by the HRS model and those not identified (see supplementary materials, Results section).

Figure 3. **The conformal predictions are not driven by nuisance covariates.** The distribution of correlations of ASD conformal scores predicted by individual networks (left) and the two ensemble models (right) with head motion (black) and age (grey) are shown across 100 bootstrap samples. Circles represent the median correlation score across bootstrap samples, vertical lines span the 5th to 25th percentile (lower bar) and 75th to 95th percentile (upper bar) of correlation scores respectively. All median correlation scores are close to zero and enclose zero within the 90% confidence interval.

Conformal prediction performance exceeds baseline model

To determine if our FC based predictive signature performed better than a simple baseline model, we repeated the conformal prediction procedure using an individual's age and in scanner head motion as input features. Following the same procedure described above, we then use the transductive conformal prediction approach to predict an ASD diagnosis only for those individuals in whom the model had high confidence. Our results show that such a baseline model did not predict ASD diagnosis with high confidence, with a median sensitivity and PPV of zero (figure S1). We thus show that the FC based network predictors performed better than a simple baseline model.

High risk signature tends to identify individuals with severe symptoms

There was a weak positive correlation between symptom severity (ADOS proxy scores) and the ASD conformality score (Pearson's $r = 0.186$, $p = 0.005$). Since only 10 individuals were identified by the model, further testing of symptom severity was limited. Exploratory analysis, detailed in the supplementary materials (Results section), indicated that the identified individuals tended to show particularly severe symptoms for their diagnostic class, but that, importantly, the model does not only identify those with severe symptoms.

DISCUSSION

This work aimed to identify an imaging biomarker of ASD that is both commonly found in the general population and confers a high risk of the disorder. Using a transductive conformal prediction approach, we identified individuals with high-confidence ASD predictions based on functional connectivity (FC). Our results showed that combined predictions from nine brain networks gave rise to a high risk FC-signature, identifying individuals with mostly severe symptoms, and pervasive underconnectivity in

an independent dataset. Compared to genetic biomarkers, our brain-first signature demonstrated higher penetrance than common mutations and higher prevalence than rare CNVs.

Model performance

This multi-network FC signature confers a PPV of 9.2%, and a more than 7-fold increased risk of ASD diagnosis in the general population where it is identified in an estimated 1 in 200 individuals, compared to a baseline ASD prevalence of 1 in 90 individuals. It is approximately two orders of magnitude less common than ASD-related SNPs [42], which confer negligible risk, and two orders of magnitude more common than rare monogenic syndromes [9], which confer very high risk (see figure 4). Studies using similar data and machine learning to classify ASD, but without the TCP approach, report accuracies that translate to PPVs of 2.4% to 2.2% [15,18]. Our FC signature's risk increase is therefore around 4 times higher than current neuroimaging models. We also out-perform the current state-of-the-art in neuroimaging for achieving high ASD PPV (8.6, the result of a large ensemble biomarker challenge), but using a simple logistic regression-based approach that is easily scalable (figure S2). To the best of our knowledge, no genetic risk signatures of autism offer comparable individual risk while being relatively common. Although similar polygenic risk signatures exist for other diseases [43], the few common ASD variants (e.g. only 5 ASD specific SNPs [42] versus 108 that have been identified for schizophrenia [22]) and the large sample sizes needed for robust polygenic risk estimation make these discoveries unlikely to happen soon.

Figure 4. **High risk signature is more common than genetic risk markers, confers higher risk than traditional imaging models, and meets the current machine learning state-of-the-art.** Monogenic syndromes (green rhombs) and recurrent Copy Number Variants (pink triangles) confer

high risk of ASD diagnosis (vertical axis), but are rare (horizontal axis). ASD related single nucleotide polymorphisms (yellow triangles) are very common, but confer negligible risk of ASD. Current imaging based predictive models (two pink circles) identify large portions of the general population with low risk of ASD. The high risk ASD signature (orange, black outline) identifies a small portion of the general population with elevated risk of ASD diagnosis, concordant with the estimated performance in the discovery data (orange plus signs), meeting the positive predictive value of 10 machine learning models combined (red circle), using a simple model.

Unlike previous imaging models which make predictions for all individuals in heterogeneous case-control populations, we limited predictions to a subset with very high-confidence ASD diagnoses. Although our model made relatively few predictions, they carried a higher risk of ASD, which compared to traditional approaches [15,18] resulted in higher specificity (99.5% vs. 72.3% and 63%, respectively) and lower sensitivity (4.2% vs. 61% and 74%, respectively). This trade-off is intentional and is a result of the TCP framework that prioritises high confidence cases.

In clinical prediction, the optimal trade-off between specificity and sensitivity depends on the goal. High specificity is prioritised if the cost of misclassification is high, such as the risk of inappropriate interventions, while high sensitivity is more valuable in contexts such as population screening. In the current work, by prioritising specificity the model identifies only the highest-confidence cases, which enables the discovery of a connectivity signature that confers high risk for ASD. This comes at the cost of lower sensitivity. We have not proposed a better machine learning model but rather addressed a different objective - the conformal prediction approach could yield similarly high specificity with previously published imaging models. Indeed, an ensemble model from an ASD prediction challenge [26] achieved a similar PPV (9.2% vs. 8.6%) but with higher sensitivity (25.4% vs. 4.2%). Our logistic regression predictor thus confers a higher individual risk than

state-of-the-art models, with much less model complexity but at the cost of lower sensitivity. The conformal prediction approach can be applied to any predictor to target high confidence predictions; it is agnostic to both feature type and algorithm. Given that SVM and deep learning approaches have been found to perform well for ASD classification [19], future studies could incorporate TCP with these approaches to improve the PPV. Similarly, TCP could be utilised in studies using other FC approaches, such as dynamic FC. Here we focus on a simple logistic regression model and static FC using Pearson correlation to improve scalability and interpretation, important for clinical applications. Overall, our results suggest that the emergence of more performant predictors opens the door to push the boundaries of high risk signatures further in the future.

The signature is driven by transmodal brain networks

Individually, the 18 brain networks did not predict ASD with high PPV. By clustering networks with correlated conformal scores and combining their predictions, we identified two sets of brain networks. The first gave rise to the high risk ASD FC signature, and included predominantly transmodal networks in the DMN and frontoparietal network, as well as subcortical areas [44]. Note that we distinguish between transmodal and unimodal networks in-line with the processing hierarchy proposed by Mesulam [45], in which lower-order unimodal areas encode basic sensory features, while higher-order transmodal networks (e.g. DMN) integrate this information into domain-general cognitive representations. Our finding aligns with previous FC-based ASD prediction models, which identified similar transmodal areas such as the temporal parietal junction and frontoparietal control network [15,22], cingulo-opercular network [46,47], and regions within the supramarginal, middle temporal, and cingulate gyri [18]. FC alterations in transmodal networks, particularly in the DMN [48–50], have been consistently reported in ASD case-control studies [13,51,52].

The second ensemble, consisting mostly of unimodal networks in the visual, auditory, and somatosensory cortices involved in sensory processing, and the ventral attention network, did not predict ASD with high PPV. Although FC alterations in unimodal areas are well-documented in ASD [53,54], they are generally less predictive of diagnosis than transmodal regions [18]. The distinction between unimodal and transmodal FC is well-established [55–57], with opposing alterations in ASD. Transmodal regions are often over-connected, while unimodal regions are under-connected [13]. This reflects a cortical gradient of functional hierarchy [58] that is altered in ASD [31,59], suggesting a dysfunctional separation between primary sensory networks and the DMN. Thus, both ensembles may capture distinct ASD risk signatures, but only one was reliably identified in our dataset.

Individuals identified by the signature tend to have severe symptoms, and underconnectivity

The high risk FC signature identified ten individuals from the independent validation dataset, nine of whom had an ASD diagnosis. These individuals generally had high symptom severity. However, their ADOS scores overlapped with those not detected by the model, indicating that the signature does not only detect severe ASD (figure 2a). This was supported by only a weak correlation between symptom severity and ASD conformality scores. Notably, the one individual without an ASD diagnosis identified by the signature had unusually severe symptoms compared to other NTC individuals, possibly reflecting a broader autism phenotype that extends into the general population [60]. Thus the signature may identify a subtype of ASD patients with particularly severe symptoms, which, since identification is based on strong dissimilarity with NTC, would be consistent with a view of neurodevelopmental disorders as a deviation from normal functioning [61,62].

The identified individuals shared a profile of pervasive functional underconnectivity in transmodal networks that gave rise to the high risk FC signature. While transmodal network

dysconnectivity, especially in the DMN [52], is consistently reported in ASD literature, its direction (over- or under-connectivity) varies [63,64], and is related to increases in symptom severity [48,65]. Notably, our finding of transmodal network underconnectivity contrasts with a case-control finding of reproducible, ASD-related prefrontal and parietal overconnectivity in a large, multi-center study [13]. These contrasting findings may highlight case-control studies' limitations in identifying ASD-related FC subtypes. Indeed, recent studies also report transmodal underconnectivity in ASD subtypes [31,32]. Our results align with other ASD prediction models that found underconnectivity between DMN subregions to be highly predictive [15,18] (but see Yahata et al.) *[46]*. It should be noted that while we limited our sample to males due to the strong sex imbalance and to ensure matching across sites, these studies included a small percentage of female participants. However, our results are also consistent with other research on males only [66–68].

Limitations

These findings must be interpreted in light of their limitations. Firstly, as mentioned, our analyses only included male individuals, a common problem in the field [59,69] due to the higher frequency with which ASD is diagnosed among male individuals [70]. Efforts are underway to include more women in ASD cohorts [71,72]. Secondly, behavioural and symptomatic characterization of those detected by the high risk signature was limited by inconsistent availability of phenotypic information. Future studies with large-scale, complete phenotyping datasets are needed for a better understanding of the cognitive and symptom profiles of neurobiologically defined at-risk individuals. Thirdly, our transductive conformal prediction model can only control for nuisance covariates available in both the reference sample and the predicted individual, so we were unable to account for site effects. However, the high-risk ASD signature identified individuals from different imaging sites with high PPV, suggesting

robustness to site differences. Finally, we estimated the general population risk of our high risk signature based on its performance in the independent dataset, identifying very few individuals (in-line with our expectations). However, we were unable to explicitly test the signature on an unselected sample to empirically determine true performance. Validating risk signatures with such a low prevalence typically requires much larger datasets [43]. Recently available general population samples with imaging data [73] should be used to validate the high risk signature and establish robust performance estimates.

Future directions

The high risk FC signature we have described offers interesting implications for future research. It identifies a cohort of individuals with similar FC alterations at high risk of an ASD diagnosis, a population in which to explore the link between neurobiological aberrations, behavioural symptoms, and genetic mechanisms in ASD. This could help disentangle the heterogeneous relationships across these levels in ASD [3,6]. Future studies should investigate the stability of this FC signature over time[74] and determine at what developmental stage it can be differentiated [75]. This requires large-scale longitudinal data, such as the Child Mind Institute Healthy Brain Network, aiming to recruit ~10,000 participants [76]. Detecting the signature in infants, especially high risk neonates such as siblings of those diagnosed with ASD, could have implications for early detection and intervention [77] . Finally, investigating this high risk ASD signature in comorbid [78] neurodevelopmental disorders may clarify the symptomatic [4], neurobiological [79,80], and genetic [42,81] overlap between these disorders and the autism spectrum.

Conclusion

We report a functional connectivity signature associated with high risk of ASD that can be detected with high positive predictive value in independent data. Application of a targeted, high-confidence prediction model was able to identify functional connectivity alterations with high penetrance, evident in a small subset of individuals. This highlights the heterogeneity of the autism spectrum, decomposing some of the contribution from functional connectivity, which traditional neuroimaging machine learning studies fail to do by optimising average accuracy. Decomposing the autism spectrum bit by bit in this manner may eventually help us understand the multitude of etiological pathways and their extension to the general population, offering avenues for further research on specific, high-risk ASD signatures.

MATERIALS AND METHODS

Ethics, consent and permissions

All imaging data used in this study were sampled from publicly available datasets. The inclusion of data in these samples was conditional on the approval of the respective local Institutional Review Board (IRB) and were shared in a de-identified form according to the requirements identified by the Health Insurance Portability and Accountability Act (HIPAA). Written informed consent/assent was obtained for all participants. The use of these data for the analyses presented in this study were approved by the “Comité Mixte d'éthique en recherche regroupement neuroimagerie du Québec” (CMER RNQ) approval number 14-15-002.

Sample

All data were sampled from the ABIDE 1 [30] and ABIDE 2 [72] dataset releases that contain imaging data for ASD patients and neurotypical controls (NTC). We used the ABIDE 1 release as a discovery dataset and retained the ABIDE 2 release as an independent validation dataset.

The final discovery dataset consisted of 452 male individuals (age 16.42, 6.91 SD, 226 ASD) from 10 recording sites. From the complete ABIDE1 dataset of 1112 individuals (age 17.04, 8.04 SD, 539 ASD) from 20 imaging sites we excluded 164 female individuals due to strong sex imbalance. Of the remaining sample, 557 individuals from 10 imaging sites were successfully preprocessed and passed visual quality control (age 16.65, 6.75 SD, 272 ASD). See figure 5 for a flowchart of participant selection. In order to control for the effects of nuisance covariates in the data without removing variance due to the ASD diagnosis, we then matched ASD and NTC individuals on age and head motion within each imaging site by propensity score matching without replacement (figure 6) [82].

Figure 5. Flowchart showing how individuals were selected from the ABIDE 1 and 2 data sets.

Figure 6. Propensity score matching schematic. First, propensity scores are estimated for each individual using selected covariates (age and head motion). We then used nearest neighbour matching, whereby individuals are matched with the closest individual from the other group that falls within an acceptable range on the propensity score axis. Data points within the dotted area represent successful matches, while those outside are excluded from further analysis. For the current study we used matching without replacement, which results in equal-sized groups. This procedure was applied separately for each data collection site.

The validation dataset consisted of 424 male individuals (age 13.66, 5,25 SD, 212 ASD) from 16 imaging sites. From the complete ABIDE2 dataset of 1114 individuals (age 14.86, 9.16 SD, 521 ASD) from 19 imaging sites, we excluded 258 female individuals due to the strong sex imbalance and to match the sample characteristics of the discovery sample. Of the remaining sample, 587 (age 13.94, 5.9, SD, 273 ASD) from 16 imaging sites were successfully preprocessed and passed visual quality control. In line with the sample selection of the discovery sample, we then matched ASD and NTC individuals on age and head motion within each imaging site using propensity score matching without replacement.

Clinical diagnosis and severity estimates

The individuals from the ABIDE1 and ABIDE2 samples included in this study were diagnosed with ASD by expert clinicians based on either the ADOS [83–85] or the Autism Diagnostic Interview - Revised [86]. Using a published conversion table [87] we converted these to proxy ADOS calibrated severity scores (ADOS-CSS), which are less influenced by an individuals' age and other demographic confounds. Proxy ADOS-CSS scores could be computed for 221 individuals (190 ASD) in the discovery and 223 (207 ASD) in the validation sample, and were strongly correlated with true ADOS-CSS scores in both (Pearson's $r = 0.90$ and 0.94 respectively, both with $p = 0.000$).

Imaging data preprocessing

Imaging data from individuals in both the discovery and independent validation sample underwent identical preprocessing through the NeuroImaging Analysis Kit (NIAK) [88] (version 1.1.3), the MINC toolkit [89] (version 1.9.15), with Octave [90] (version 4.2.1), and Ubuntu [91] (version 16.04.2LTS), running inside a Singularity containerized environment [92] (version 2.6.1). Preprocessing of MRI data

was executed in parallel on the Cedar supercomputer [93], using the Pipeline System for Octave and Matlab (PSOM) [94] (version 2.3.1). In short, functional time series were corrected for in-scanner head motion and registered to the MNI152 stereotaxic space [95]. Slow time drift signals were modelled on the continuous time series by a discrete cosine transformation and removed after censoring of time frames with excessive (> 0.4mm) head motion [96], together with nuisance covariates of the average white matter, and cerebrospinal fluid signals, and the first principal components (accounting for 95% of variance) of the six degrees of freedom head motion estimates and their squares [97]. The preprocessed imaging data were visually quality controlled to ensure the quality of the data. The QC was performed by a trained rater according to our in-lab standardised QC protocol [98] using a guided QC environment [99].

Functional connectivity estimation

Seed to voxel FC was estimated for functional brain networks defined in the MIST_20 atlas [100]. The MIST_20 atlas represents 20 large, spatially distributed subcomponents of canonical FC networks. A large number of individuals were found to have incomplete coverage of the cerebellum, and so we excluded 2 networks that were part of the cerebellum. For each of the remaining 18 brain networks, the average within-network time series was correlated with the time series of all non-cerebellar voxels using Pearson's correlation.

High confidence prediction

In order to achieve a high specificity of ASD predictions, we limit predictions to cases where our model has a high level of confidence that an individual is not a neurotypical participant (NTC). We compute the confidence of the prediction by applying the transductive conformal prediction (TCP) approach [36,41]. TCP computes how "usual" (or conformal) the features of an unclassified individual

(UCI) would be if we assumed either an ASD or NTC label, compared to already classified individuals with these labels. That is, given an individual that we want to classify as either ASD or neurotypical, the conformal predictor asks: "how unusual would this individual be, if they were an individual with ASD?" and "how unusual would they be, if they were a neurotypical individual?". The predictor then answers each of these questions by comparing the individual to known individuals with ASD, and neurotypical individuals, respectively. In this way, two conformality scores for each individual are computed, one for each of the two possible label classes. The predicted conformality score for each label then allows us to only make predictions when we have a high level of confidence in rejecting one label, i.e. if an individual would be very "unusual" as a NTC participant (see figure 3). More technical introductory accounts of the conformal prediction logic can be found in Gammerman et al. [101] and Shafer et al. [102].

In contrast to an inductive classification approach, where a statistical model is first learned based on the properties of the reference set and then applied to new data, in a transductive classification, no model is learned and each new individual is classified directly and separately by comparing it to the properties of each class (ASD and NTC) in the reference set, and choosing the class it most conforms to [103]. Each unclassified individual (UCI) therefore has to be treated in the exact same way to ensure the independence of each classification. See figure 7 for a schematic.

Figure 7. Schematic of transductive conformal prediction. A) Circles represent individuals in the dataset, either autism spectrum diagnosis (ASD) (purple) or neurotypical control (NTC) (grey). One individual from the sample at a time is designated the "unclassified individual" (UCI) (orange). Group level nuisance regression and dimensionality reduction is conducted on the entire sample, including the UCI. The black circle represents that each individual in the dataset is designated the UCI in turn. **B)** A

first logistic regression is fitted to predict an ASD label. A scaling factor is used to increase specificity by minimising false positives. **C)** A second logistic regression is fitted to predict a label of neurotypical controls (NTC). The conformal scores are determined based on how unusual the UCI is compared to each group, calculated as the percentage of individuals that are known to have the assumed label and have an equal or lower predicted value than the UCI. The shaded areas in plots **B** and **C** visually indicate these individuals known to have the assumed label who also have a lower or equal predicted score than the UCI. **D)** To limit ASD predictions to the most confident cases, predictions are only made if the ASD conformal score is > 5% and NTC conformal score is < 5%. This process is repeated for each UCI independently.

Regression of nuisance covariates

To account for potential confounding effects, we combine the UCI and the reference sample and use ordinary least squares regression to remove the group level average connectivity and the linear effect of age and head motion from the network FC maps, retaining the residuals for further analysis.

Dimensionality reduction

Previous works have shown the capacity of FC subtypes to capture disease-related FC variability, e.g Easson et al. [104], and the utility of hierarchical ensemble methods for identifying neuroimaging-based subtypes [105]. We therefore identify the five subtypes of FC variability across both the UCI and the reference sample by hierarchical agglomerative clustering of spatially correlated, individual FC maps. For each individual we then compute the spatial similarity with the average FC map of each of the five FC subtypes.

Estimation of conformality and classification

The individual conformality estimate for either clinical label (i.e., ASD or NTC) was then computed similarly to the previous work of Nouretdinov et al. [41]. In short, we first assumed an ASD label for each UCI and then fit a logistic regression to predict ASD for both the UCI and the reference sample, using the previously estimated similarity with FC subtypes as features. To reflect the fact that we wanted the model to make as few false positive errors as possible, we weighed the predicted values of ASD individuals by a large scaling factor (w(ASD) = 10^16). This forced the prediction model to only be concerned with the identification of ASD cases, with high specificity, at the expense of possible identification of NTC individuals. We computed the ASD conformal score for each UCI as the percentage of ASD individuals in the reference sample with a predicted value equal to or smaller than the one that was predicted for that UCI. In other words: if most ASD individuals had larger predicted values than the UCI, then the UCI did not conform to the ASD cohort and was an unusual ASD case, and thus the ASD conformal score would have been small due to the individual not "conforming" to the reference cohort of ASD individuals. An analogous process was then repeated to compute the NTC conformal score of the UCI.

We rejected a label (i.e., ASD or NTC) if the corresponding estimated conformal score was below a critical threshold of 5%. We predicted ASD with high confidence for only those individuals who had NTC conformal scores below the critical threshold and ASD conformal scores equal or greater than the critical threshold.

Performance assessment

To assess the quality of the classification we computed sensitivity, specificity, positive predictive value (PPV), risk ratio (RR), odds ratio (OR), and the Sørensen–Dice coefficient. Detailed equations and

explanations are provided in the supplementary materials. Briefly, PPV depends on the prevalence of ASD in the sample, and estimates the individual probability of a true ASD diagnosis. If the model indicates any risk, the risk of ASD is higher for someone identified by the model than for someone not identified, measured by the RR. The OR is similar but does not depend on prevalence. The Sørensen–Dice coefficient evaluates the overlap between true ASD cases and model predictions, ranging from 0 (no overlap) to 1 (complete overlap). See figure 8 for a schematic of PPV and the Sørensen–Dice coefficient in relation to different ASD risk markers.

Figure 8. Schematic representation of properties of different ASD risk markers. A) A set of individuals in the population is found to express the risk marker (grey) and is thus labelled. Among the set of individuals with ASD in the population (purple), some are also labelled by the risk marker (blue). Risk markers differ in the amount of labelled individuals from very few (left column) to very many (right column). **B)** Different metrics exist to evaluate the performance of the risk marker. The ratio of ASD individuals among the labelled individuals (PPV) can be very high if only a very few individuals are labelled by the risk marker (e.g. in monogenic syndromes with high risk for ASD, left column). However, the degree of congruence of ASD and labelled individuals (dice coefficient) would be very low, because of the large number of unlabeled ASD individuals. Conversely, a risk marker that labels very many individuals may capture more ASD individuals and have a moderately higher dice coefficient, but would have a very low ratio of ASD to labelled individuals (PPV) and thus confer very low individual risk (e.g. existing imaging based models, right column). The HRS approach presented here labels fewer individuals than current imaging models but those individuals are more likely to have ASD, resulting in higher PPV and comparable dice coefficients.

Bootstrap estimation

We estimated the model performance of each brain network predictor through bootstrap subsampling of the discovery data set. We drew two random bootstrap samples from the discovery data set and assigned one to be the reference data set and the other to be the prediction data set. The ASD diagnosis of each individual in the prediction data set was then separately predicted based on the individuals in the reference data set, following preprocessing, feature extraction and training as described above. We repeated this process 100 times for each brain network and computed the average performance metrics of each predictor across bootstraps. See e.g., Efron et al. [106] regarding bootstrap predictor evaluation methods.

Combination of correlated conformal predictions

To identify similarities of conformal predictions between the 18 functional brain networks, we computed the pairwise correlation of ASD non-conformity. We then used hierarchical agglomerative clustering to identify groups of networks with correlated ASD conformal score estimates. We selected a seven and two cluster solution based on a visual inspection of the network by network correlation matrix.

Within each cluster of networks, conformal score estimates (i.e., probability estimates of non-conformity with each class label) were combined using the p-value averaging methods of Vovk & Wang [107]. Specifically, we averaged over the p-values that are associated within each network using the squared-mean merging function, which produces a valid aggregate p-value from the combination of any finite number of potentially correlated individual p-values. This requirement of validity is important in order to maintain the conformity properties when using these cluster-aggregated p-values as inputs in a conformal predictor.

The aggregation of *p*-values was observed to average over the information that are inherent in each of the contributing *p*-values. As such, less informative network elements tended to decrease the explanatory power of the more informative elements. The overall effect was that the cluster non-conformity threshold tended to be conservative in identifying interesting observations, when compared to the same threshold value, applied to individual networks. In order to mitigate against this conservative effect, we used a more liberal threshold for cluster-aggregated *p*-values, than those used for individual networks. That is, we adjusted the critical non-conformal threshold to 0.2 from 0.05.

Validation on the independent dataset

The HRS identified on the discovery sample was then validated on the independent validation sample. To do so, the ASD and NTC non-conformity estimate of each individual in the validation sample was computed by using the individuals of the discovery sample as the reference cohort. Each individual in the validation sample was predicted independently after group level nuisance regression and dimensionality reduction with respect to the reference sample.

Estimation of model performance in the general population

The discovery and validation sample had equal rates of ASD patients and NTC individuals (i.e., 1 ASD for each 1 NTC). The prevalence of ASD in the general population is however much lower (1 ASD for each 89 NTC). Based on the estimated specificity and sensitivity of our model in the independent validation sample, we estimated the positive predictive value (PPV_{ASD}) of the HRS in the general population.

Acknowledgments

This research was supported by computation resources of Calcul Quebec and Compute Canada. We thank Yu Zhang and Gleb Bezgin for helpful discussions. For their feedback on the writing of this manuscript we want to thank Julie Boyle and Jonas Nitschke. We thank the ABIDE consortium for making publicly available the large datasets that this study was based on.

Funding

Azrieli Foundation (3388)

Australian Research Council (DE170101134 and DP180101192)

Brain Canada Multi Investigator Research Initiative (MIRI)

Canadian Consortium on Neurodegeneration in Aging (Graduate Student Funding)

Canadian Open Neuroscience Platform (Student Scholar Award)

Centre de recherche de l'Institut universitaire de geriatrie de Montreal (Graduate Student Funding)

Courtouis Neuromod Foundation (Graduate Student Funding)

Fonds de Recherche du Québec - Santé

Healthy Brains, Healthy Lives (Graduate Student Funding)

Institut de valorisation des données (IVADO) Postdoctoral Fellowship

Competing interests

Authors declare that they have no competing interests.

Data availability

All data used in this manuscript are available for download at https://zenodo.org/records/15517157. These data can be used to fully reproduce the analyses. Alternatively, figures can be reproduced using the precomputed results also available at the same link.

Availability of supporting source code and requirements

All source code and software requirements are available at https://github.com/SIMEXP/autism-signature, and are also registered on WorkflowHub: https://doi.org/10.48546/workflowhub.workflow.1336.1.

Project name: High-precision machine learning identifies a reproducible functional connectivity signature of autism spectrum diagnosis in a subset of individuals

Project home page: https://github.com/SIMEXP/autism-signature

Operating system(s): Platform independent

Programming language: Python, R

Other requirements: Jupyter notebook (used for supplementary analyses and figures)

License: MIT

RRID: N/A

Bio.tools ID: N/A

References

1. Bai D, Yip BHK, Windham GC, Sourander A, Francis R, Yoffe R, et al.. Association of Genetic and Environmental Factors With Autism in a 5-Country Cohort. *JAMA Psychiatry*. jamanetwork.com; 2019; doi: 10.1001/jamapsychiatry.2019.1411.

2. American Psychiatric Association D: Diagnostic and statistical manual of mental disorders: DSM-5. academia.edu; https://www.academia.edu/download/38718268/csl6820_21.pdf (2013). Accessed 2024 Jan 22.

3. Lombardo MV, Lai M-C, Baron-Cohen S. Big data approaches to decomposing heterogeneity across the autism spectrum. *Mol Psychiatry*. 2019; doi: 10.1038/s41380-018-0321-0.

4. Grzadzinski R, Di Martino A, Brady E, Mairena MA, O'Neale M, Petkova E, et al.. Examining autistic traits in children with ADHD: does the autism spectrum extend to ADHD? *J Autism Dev Disord*. 2011; doi: 10.1007/s10803-010-1135-3.

5. Park MTM, Raznahan A, Shaw P, Gogtay N, Lerch JP, Chakravarty MM. Neuroanatomical phenotypes in mental illness: identifying convergent and divergent cortical phenotypes across autism, ADHD and schizophrenia. *J Psychiatry Neurosci*. 2018; doi: 10.1503/jpn.170094.

6. Moreau CA, Urchs SGW, Kuldeep K, Orban P, Schramm C, Dumas G, et al.. Mutations associated with neuropsychiatric conditions delineate functional brain connectivity dimensions contributing to autism and schizophrenia. *Nat Commun*. Nature Publishing Group; 2020; doi: 10.1038/s41467-020-18997-2.

7. Cuthbert BN, Insel TR. Toward the future of psychiatric diagnosis: the seven pillars of RDoC. *BMC Med*. 2013; doi: 10.1186/1741-7015-11-126.

8. Sanders SJ, Sahin M, Hostyk J, Thurm A, Jacquemont S, Avillach P, et al.. A framework for the investigation of rare genetic disorders in neuropsychiatry. *Nat Med*. 2019; doi: 10.1038/s41591-019-0581-5.

9. de la Torre-Ubieta L, Won H, Stein JL, Geschwind DH. Advancing the understanding of autism disease mechanisms through genetics. *Nat Med*. 2016; doi: 10.1038/nm.4071.

10. Maher B: Personal genomes: The case of the missing heritability. Nature Publishing Group UK. http://dx.doi.org/10.1038/456018a (2008). Accessed 2024 Jan 22.

11. Manolio TA, Collins FS, Cox NJ, Goldstein DB, Hindorff LA, Hunter DJ, et al.. Finding the missing heritability of complex diseases. *Nature*. 2009; doi: 10.1038/nature08494.

12. Castellanos FX, Di Martino A, Craddock RC, Mehta AD, Milham MP. Clinical applications of the functional connectome. *Neuroimage*. Elsevier; 2013; doi: 10.1016/j.neuroimage.2013.04.083.

13. Holiga Š, Hipp JF, Chatham CH, Garces P, Spooren W, D'Ardhuy XL, et al.. Patients with autism spectrum disorders display reproducible functional connectivity alterations. *Sci Transl Med*. 2019; doi: 10.1126/scitranslmed.aat9223.

14. Luo N, Sui J, Abrol A, Lin D, Chen J, Vergara VM, et al.. Age-related structural and functional variations in 5,967 individuals across the adult lifespan. *Hum Brain Mapp*. Wiley; 2020; doi: 10.1002/hbm.24905.

15. Abraham A, Milham MP, Di Martino A, Craddock RC, Samaras D, Thirion B, et al.. Deriving reproducible

biomarkers from multi-site resting-state data: An Autism-based example. *Neuroimage*. Neuroimage; 2017; doi: 10.1016/j.neuroimage.2016.10.045.

16. Chen H, Duan X, Liu F, Lu F, Ma X, Zhang Y, et al.. Multivariate classification of autism spectrum disorder using frequency-specific resting-state functional connectivity—A multi-center study. *Progress in Neuro-Psychopharmacology and Biological Psychiatry*. Elsevier BV; 2016; doi: 10.1016/j.pnpbp.2015.06.014.

17. Yang X, Zhang N, Schrader P. A study of brain networks for autism spectrum disorder classification using resting-state functional connectivity. *Mach Learn Appl*. Elsevier BV; 2022; doi: 10.1016/j.mlwa.2022.100290.

18. Heinsfeld AS, Franco AR, Craddock RC, Buchweitz A, Meneguzzi F. Identification of autism spectrum disorder using deep learning and the ABIDE dataset. *Neuroimage Clin*. 2018; doi: 10.1016/j.nicl.2017.08.017.

19. Liu M, Li B, Hu D. Autism spectrum disorder studies using fMRI data and machine learning: A review. *Front Neurosci*. Frontiers Media SA; 2021; doi: 10.3389/fnins.2021.697870.

20. Liang L, Dong G, Li C, Wen D, Zhao Y, Li J. Improving autism spectrum disorder prediction by fusion of multiple measures of resting-state functional MRI data. *Annu Int Conf IEEE Eng Med Biol Soc*. IEEE; 2022; doi: 10.1109/EMBC48229.2022.9871167.

21. Thomas RM, Gallo S, Cerliani L, Zhutovsky P, El-Gazzar A, van Wingen G. Classifying autism spectrum disorder using the temporal statistics of resting-state functional MRI data with 3D convolutional neural networks. *Front Psychiatry*. Frontiers Media SA; 2020; doi: 10.3389/fpsyt.2020.00440.

22. ElNakieb Y, Ali MT, Elnakib A, Shalaby A, Mahmoud A, Soliman A, et al.. Understanding the Role of Connectivity Dynamics of Resting-State Functional MRI in the Diagnosis of Autism Spectrum Disorder: A Comprehensive Study. *Bioengineering (Basel)*. 2023; doi: 10.3390/bioengineering10010056.

23. Price T, Wee C-Y, Gao W, Shen D. Multiple-network classification of childhood autism using functional connectivity dynamics. *Med Image Comput Comput Assist Interv*. Springer International Publishing; 2014; doi: 10.1007/978-3-319-10443-0_23.

24. Zhao F, Chen Z, Rekik I, Lee S-W, Shen D. Diagnosis of autism spectrum disorder using central-moment features from low- and high-order dynamic resting-state functional connectivity networks. *Front Neurosci*. Frontiers Media SA; 2020; doi: 10.3389/fnins.2020.00258.

25. Vabalas A, Gowen E, Poliakoff E, Casson AJ. Machine learning algorithm validation with a limited sample size. *PLoS One*. Public Library of Science (PLoS); 2019; doi: 10.1371/journal.pone.0224365.

26. Traut N, Heuer K, Lemaître G, Beggiato A, Germanaud D, Elmaleh M, et al.. Insights from an autism imaging biomarker challenge: Promises and threats to biomarker discovery. *Neuroimage*. 2022; doi: 10.1016/j.neuroimage.2022.119171.

27. Varoquaux G. Cross-validation failure: Small sample sizes lead to large error bars. *Neuroimage*. 2018; doi: 10.1016/j.neuroimage.2017.06.061.

28. Wolfers T, Buitelaar JK, Beckmann CF, Franke B, Marquand AF. From estimating activation locality to predicting disorder: A review of pattern recognition for neuroimaging-based psychiatric diagnostics. *Neurosci Biobehav Rev*. 2015; doi: 10.1016/j.neubiorev.2015.08.001.

29. Xu M, Calhoun V, Jiang R, Yan W, Sui J. Brain imaging-based machine learning in autism spectrum disorder: methods and applications. *J Neurosci Methods*. 2021; doi: 10.1016/j.jneumeth.2021.109271.

30. Di Martino A, Yan C-G, Li Q, Denio E, Castellanos FX, Alaerts K, et al.. The autism brain imaging data exchange: towards a large-scale evaluation of the intrinsic brain architecture in autism. *Mol Psychiatry*. 2014; doi: 10.1038/mp.2013.78.

31. Urchs SGW, Tam A, Orban P, Moreau C, Benhajali Y, Nguyen HD, et al.. Functional connectivity subtypes associate robustly with ASD diagnosis. *Elife*. 2022; doi: 10.7554/eLife.56257.

32. Tang S, Sun N, Floris DL, Zhang X, Di Martino A, Yeo BTT. Reconciling Dimensional and Categorical Models of Autism Heterogeneity: A Brain Connectomics and Behavioral Study. *Biol Psychiatry*. 2020; doi: 10.1016/j.biopsych.2019.11.009.

33. Qi S, Morris R, Turner JA, Fu Z, Jiang R, Deramus TP, et al.. Common and unique multimodal covarying patterns in autism spectrum disorder subtypes. *Mol Autism*. Springer Science and Business Media LLC; 2020; doi: 10.1186/s13229-020-00397-4.

34. Hahamy A, Behrmann M, Malach R. The idiosyncratic brain: distortion of spontaneous connectivity patterns in autism spectrum disorder. *Nat Neurosci*. 2015; doi: 10.1038/nn.3919.

35. Vapnik VN. Statistical learning theory J Wiley New York. 1998;

36. Vovk V, Gammerman A, Shafer G. Algorithmic Learning in a Random World. Springer International Publishing;

37. Alvarsson J, Arvidsson McShane S, Norinder U, Spjuth O. Predicting with confidence: Using conformal prediction in drug discovery. *J Pharm Sci*. Elsevier BV; 2021; doi: 10.1016/j.xphs.2020.09.055.

38. Olsson H, Kartasalo K, Mulliqi N, Capuccini M, Ruusuvuori P, Samaratunga H, et al.. Estimating diagnostic uncertainty in artificial intelligence assisted pathology using conformal prediction. *Nat Commun*. Springer Science and Business Media LLC; 2022; doi: 10.1038/s41467-022-34945-8.

39. Pereira T, Mendon\ca A, Ferreira F, Madeira S, Guerreiro M. Towards a reliable prediction of conversion from Mild Cognitive Impairment to Alzheimer's Disease: stepwise learning using time windows. *Medical Informatics and Healthcare*. PMLR; p. 19–26.

40. Lambrou A, Papadopoulos H, Kyriacou E, Pattichis CS, Pattichis MS, Gammerman A, et al.. Assessment of stroke risk based on morphological ultrasound image analysis with conformal prediction. *IFIP Advances in Information and Communication Technology*. Berlin, Heidelberg: Springer Berlin Heidelberg;

41. Nouretdinov I, Costafreda SG, Gammerman A, Chervonenkis A, Vovk V, Vapnik V, et al.. Machine learning classification with confidence: application of transductive conformal predictors to MRI-based diagnostic and prognostic markers in depression. *Neuroimage*. Elsevier; 2011; doi: 10.1016/j.neuroimage.2010.05.023.

42. Grove J, Ripke S, Als TD, Mattheisen M, Walters RK, Won H, et al.. Identification of common genetic risk variants for autism spectrum disorder. *Nat Genet*. 2019; doi: 10.1038/s41588-019-0344-8.

43. Khera AV, Chaffin M, Aragam KG, Haas ME, Roselli C, Choi SH, et al.. Genome-wide polygenic scores for common diseases identify individuals with risk equivalent to monogenic mutations. *Nat Genet*. 2018; doi: 10.1038/s41588-018-0183-z.

44. Alves PN, Foulon C, Karolis V, Bzdok D, Margulies DS, Volle E, et al.. An improved neuroanatomical model of the default-mode network reconciles previous neuroimaging and neuropathological findings. *Commun Biol*. 2019; doi: 10.1038/s42003-019-0611-3.

45. Mesulam M. Neurocognitive networks and selectively distributed processing. *Rev Neurol (Paris)*. Rev Neurol (Paris); 150:564–91994;

46. Yahata N, Morimoto J, Hashimoto R, Lisi G, Shibata K, Kawakubo Y, et al.. A small number of abnormal brain connections predicts adult autism spectrum disorder. *Nat Commun*. nature.com; 2016; doi: 10.1038/ncomms11254.

47. Reiter MA, Jahedi A, Jac Fredo AR, Fishman I, Bailey B, Müller R-A. Performance of machine learning classification models of autism using resting-state fMRI is contingent on sample heterogeneity. *Neural Comput Appl*. 2021; doi: 10.1007/s00521-020-05193-y.

48. Assaf M, Jagannathan K, Calhoun VD, Miller L, Stevens MC, Sahl R, et al.. Abnormal functional connectivity of default mode sub-networks in autism spectrum disorder patients. *Neuroimage*. 2010; doi: 10.1016/j.neuroimage.2010.05.067.

49. Washington SD, Gordon EM, Brar J, Warburton S, Sawyer AT, Wolfe A, et al.. Dysmaturation of the default mode network in autism. *Hum Brain Mapp*. 2014; doi: 10.1002/hbm.22252.

50. Yang B, Wang M, Zhou W, Wang X, Chen S, Potenza MN, et al.. Disrupted network integration and segregation involving the default mode network in autism spectrum disorder. *J Affect Disord*. 2023; doi: 10.1016/j.jad.2022.11.083.

51. Just MA, Cherkassky VL, Keller TA, Kana RK, Minshew NJ. Functional and anatomical cortical underconnectivity in autism: evidence from an FMRI study of an executive function task and corpus callosum morphometry. *Cereb Cortex*. 2007; doi: 10.1093/cercor/bhl006.

52. Monk CS, Peltier SJ, Wiggins JL, Weng S-J, Carrasco M, Risi S, et al.. Abnormalities of intrinsic functional connectivity in autism spectrum disorders. *Neuroimage*. 2009; doi: 10.1016/j.neuroimage.2009.04.069.

53. Isakoglou C, Haak KV, Wolfers T, Floris DL, Llera A, Oldehinkel M, et al.. Fine-grained topographic organization within somatosensory cortex during resting-state and emotional face-matching task and its association with ASD traits. bioRxiv.

54. Oldehinkel M, Mennes M, Marquand A, Charman T, Tillmann J, Ecker C, et al.. Altered Connectivity Between Cerebellum, Visual, and Sensory-Motor Networks in Autism Spectrum Disorder: Results from the EU-AIMS Longitudinal European Autism Project. *Biol Psychiatry Cogn Neurosci Neuroimaging*. 2019; doi: 10.1016/j.bpsc.2018.11.010.

55. Buckner RL, DiNicola LM. The brain's default network: updated anatomy, physiology and evolving insights. *Nat Rev Neurosci*. 2019; doi: 10.1038/s41583-019-0212-7.

56. Fox MD, Snyder AZ, Vincent JL, Corbetta M, Van Essen DC, Raichle ME. The human brain is intrinsically organized into dynamic, anticorrelated functional networks. *Proc Natl Acad Sci U S A*. 2005; doi: 10.1073/pnas.0504136102.

57. Raichle ME, MacLeod AM, Snyder AZ, Powers WJ, Gusnard DA, Shulman GL. A default mode of brain function. *Proc Natl Acad Sci U S A*. 2001; doi: 10.1073/pnas.98.2.676.

58. Margulies DS, Ghosh SS, Goulas A, Falkiewicz M, Huntenburg JM, Langs G, et al.. Situating the default-mode network along a principal gradient of macroscale cortical organization. *Proc Natl Acad Sci U S A*. 2016; doi: 10.1073/pnas.1608282113.

59. Hong S-J, Vos de Wael R, Bethlehem RAI, Lariviere S, Paquola C, Valk SL, et al.. Atypical functional connectome hierarchy in autism. *Nat Commun*. 2019; doi: 10.1038/s41467-019-08944-1.

60. Baron-Cohen S, Wheelwright S, Skinner R, Martin J, Clubley E. The autism-spectrum quotient (AQ): evidence from Asperger syndrome/high-functioning autism, males and females, scientists and mathematicians. *J Autism Dev Disord*. 2001; doi: 10.1023/a:1005653411471.

61. Marquand AF, Kia SM, Zabihi M, Wolfers T, Buitelaar JK, Beckmann CF. Conceptualizing mental disorders as deviations from normative functioning. *Mol Psychiatry*. 2019; doi: 10.1038/s41380-019-0441-1.

62. Shan X, Uddin LQ, Xiao J, He C, Ling Z, Li L, et al.. Mapping the Heterogeneous Brain Structural Phenotype of Autism Spectrum Disorder Using the Normative Model. *Biol Psychiatry*. 2022; doi: 10.1016/j.biopsych.2022.01.011.

63. Hull JV, Dokovna LB, Jacokes ZJ, Torgerson CM, Irimia A, Van Horn JD. Resting-State Functional Connectivity in Autism Spectrum Disorders: A Review. *Front Psychiatry*. 2016; doi: 10.3389/fpsyt.2016.00205.

64. Padmanabhan A, Lynch CJ, Schaer M, Menon V. The Default Mode Network in Autism. *Biol Psychiatry Cogn Neurosci Neuroimaging*. 2017; doi: 10.1016/j.bpsc.2017.04.004.

65. Supekar K, Uddin LQ, Khouzam A, Phillips J, Gaillard WD, Kenworthy LE, et al.. Brain hyperconnectivity in children with autism and its links to social deficits. *Cell Rep*. 2013; doi: 10.1016/j.celrep.2013.10.001.

66. Jones TB, Bandettini PA, Kenworthy L, Case LK, Milleville SC, Martin A, et al.. Sources of group differences in functional connectivity: an investigation applied to autism spectrum disorder. *Neuroimage*. Academic Press; 2010; doi: 10.1016/j.neuroimage.2009.07.051.

67. Joshi G, Arnold Anteraper S, Patil KR, Semwal M, Goldin RL, Furtak SL, et al.. Integration and segregation of default mode network resting-state functional connectivity in transition-age males with high-functioning autism spectrum disorder: A proof-of-concept study. *Brain Connect*. Mary Ann Liebert, Inc. 140 Huguenot Street, 3rd Floor New Rochelle, NY 10801 USA; 2017; doi: 10.1089/brain.2016.0483.

68. Jung M, Kosaka H, Saito DN, Ishitobi M, Morita T, Inohara K, et al.. Default mode network in young male adults with autism spectrum disorder: relationship with autism spectrum traits. *Mol Autism*. Springer Science and Business Media LLC; 2014; doi: 10.1186/2040-2392-5-35.

69. Khundrakpam BS, Lewis JD, Kostopoulos P, Carbonell F, Evans AC. Cortical Thickness Abnormalities in Autism Spectrum Disorders Through Late Childhood, Adolescence, and Adulthood: A Large-Scale MRI Study. *Cereb Cortex*. 2017; doi: 10.1093/cercor/bhx038.

70. Lai M-C, Lombardo MV, Baron-Cohen S. Autism. *Lancet*. 2014; doi: 10.1016/S0140-6736(13)61539-1.

71. Bedford SA, Park MTM, Devenyi GA, Tullo S, Germann J, Patel R, et al.. Large-scale analyses of the relationship between sex, age and intelligence quotient heterogeneity and cortical morphometry in autism spectrum disorder. *Mol Psychiatry*. 2020; doi: 10.1038/s41380-019-0420-6.

72. Di Martino A, O'Connor D, Chen B, Alaerts K, Anderson JS, Assaf M, et al.. Enhancing studies of the connectome in autism using the autism brain imaging data exchange II. *Sci Data*. nature.com; 2017; doi: 10.1038/sdata.2017.10.

73. Bycroft C, Freeman C, Petkova D, Band G, Elliott LT, Sharp K, et al.. The UK Biobank resource with deep phenotyping and genomic data. *Nature*. 2018; doi: 10.1038/s41586-018-0579-z.

74. Jacob S, Wolff JJ, Steinbach MS, Doyle CB, Kumar V, Elison JT. Neurodevelopmental heterogeneity and computational approaches for understanding autism. *Transl Psychiatry*. 2019; doi: 10.1038/s41398-019-0390-0.

75. Emerson RW, Adams C, Nishino T, Hazlett HC, Wolff JJ, Zwaigenbaum L, et al.. Functional neuroimaging of high-risk 6-month-old infants predicts a diagnosis of autism at 24 months of age. *Sci Transl Med*. 2017; doi: 10.1126/scitranslmed.aag2882.

76. Alexander LM, Escalera J, Ai L, Andreotti C, Febre K, Mangone A, et al.. An open resource for transdiagnostic research in pediatric mental health and learning disorders. *Sci Data*. 2017; doi: 10.1038/sdata.2017.181.

77. Ciarrusta J, Dimitrova R, Batalle D, O'Muircheartaigh J, Cordero-Grande L, Price A, et al.. Emerging functional connectivity differences in newborn infants vulnerable to autism spectrum disorders. *Transl Psychiatry*. 2020; doi: 10.1038/s41398-020-0805-y.

78. Simonoff E, Pickles A, Charman T, Chandler S, Loucas T, Baird G. Psychiatric disorders in children with autism spectrum disorders: prevalence, comorbidity, and associated factors in a population-derived sample. *J Am Acad Child Adolesc Psychiatry*. 2008; doi: 10.1097/CHI.0b013e318179964f.

79. de Lange SC, Scholtens LH, Alzheimer's Disease Neuroimaging Initiative, van den Berg LH, Boks MP, Bozzali M, et al.. Shared vulnerability for connectome alterations across psychiatric and neurological brain disorders. *Nat Hum Behav*. 2019; doi: 10.1038/s41562-019-0659-6.

80. van den Heuvel MP, Sporns O. A cross-disorder connectome landscape of brain dysconnectivity. *Nat Rev Neurosci*. 2019; doi: 10.1038/s41583-019-0177-6.

81. Cross-Disorder Group of the Psychiatric Genomics Consortium, Lee SH, Ripke S, Neale BM, Faraone SV, Purcell SM, et al.. Genetic relationship between five psychiatric disorders estimated from genome-wide SNPs. *Nat Genet*. 2013; doi: 10.1038/ng.2711.

82. Rosenbaum PR, Rubin DB. Constructing a Control Group Using Multivariate Matched Sampling Methods That Incorporate the Propensity Score. *Am Stat*. 1985; doi: 10.1080/00031305.1985.10479383.

83. Gotham K, Risi S, Pickles A, Lord C. The Autism Diagnostic Observation Schedule: revised algorithms for improved diagnostic validity. *J Autism Dev Disord*. 2007; doi: 10.1007/s10803-006-0280-1.

84. Lord C, Rutter M, DiLavore P, Risi S, Gotham K. Autism diagnostic observation schedule–2nd edition (ADOS-2). *Los Angeles, CA: Western*. 2012;

85. Lord C, Risi S, Lambrecht L, Cook EH, Leventhal BL, DiLavore PC, et al.. The Autism Diagnostic Observation Schedule—Generic: A Standard Measure of Social and Communication Deficits Associated with the Spectrum of Autism. *J Autism Dev Disord*. 2000; doi: 10.1023/A:1005592401947.

86. Lord C, Rutter M, Le Couteur A. Autism Diagnostic Interview-Revised: a revised version of a diagnostic interview for caregivers of individuals with possible pervasive developmental disorders. *J Autism Dev Disord*. 1994; doi: 10.1007/BF02172145.

87. Moradi E, Khundrakpam B, Lewis JD, Evans AC, Tohka J. Predicting symptom severity in autism spectrum disorder based on cortical thickness measures in agglomerative data. *Neuroimage*. 2017; doi: 10.1016/j.neuroimage.2016.09.049.

88. Bellec P, Carbonell FM, Perlbarg V, Lepage C, Lyttelton O, Fonov V, et al.. A neuroimaging analysis kit for

Matlab and Octave. *Proceedings of the 17th International Conference on Functional Mapping of the Human Brain*.

89. : BIC-MNI Software repository. https://bic-mni.github.io/ Accessed 2024 Oct 7.

90. : GNU Octave. https://octave.org/index Accessed 2024 Oct 7.

91. : Enterprise Open Source and Linux. Ubuntu. https://ubuntu.com/ Accessed 2024 Oct 7.

92. Kurtzer GM, Sochat V, Bauer MW. Singularity: Scientific containers for mobility of compute. *PLoS One*. 2017; doi: 10.1371/journal.pone.0177459.

93. : Cedar. https://docs.alliancecan.ca/wiki/Cedar Accessed 2024 Oct 7.

94. Bellec P, Lavoie-Courchesne S, Dickinson P, Lerch JP, Zijdenbos AP, Evans AC. The pipeline system for Octave and Matlab (PSOM): a lightweight scripting framework and execution engine for scientific workflows. *Front Neuroinform*. Frontiers Media SA; 2012; doi: 10.3389/fninf.2012.00007.

95. Evans AC, Kamber M, Collins DL, MacDonald D. An MRI-Based Probabilistic Atlas of Neuroanatomy. *Magnetic Resonance Scanning and Epilepsy*. Springer, Boston, MA;

96. Power JD, Barnes KA, Snyder AZ, Schlaggar BL, Petersen SE. Spurious but systematic correlations in functional connectivity MRI networks arise from subject motion. *Neuroimage*. 2012; doi: 10.1016/j.neuroimage.2011.10.018.

97. Giove F, Gili T, Iacovella V, Macaluso E, Maraviglia B. Images-based suppression of unwanted global signals in resting-state functional connectivity studies. *Magn Reson Imaging*. 2009; doi: 10.1016/j.mri.2009.06.004.

98. Benhajali Y, Badhwar A, Spiers H, Urchs S, Armoza J, Ong T, et al.. A standardized protocol for efficient and reliable quality control of brain registration in functional MRI studies.

99. Urchs S, Armoza J, Benhajali Y, Bellec P. dashqc-fmri - an interactive web dashboard for manual quality control.

100. Urchs S, Armoza J, Benhajali Y, St-Aubin J, Orban P, Bellec P. MIST: A multi-resolution parcellation of functional brain networks. *MNI Open Res*. 2017; doi: 10.12688/mniopenres.12767.1.

101. Gammerman A, Vovk V. Hedging Predictions in Machine Learning: The Second Computer Journal Lecture. *Comput J*. Oxford Academic; 2007; doi: 10.1093/comjnl/bxl065.

102. Shafer G, Vovk V. A tutorial on conformal prediction. arXiv [cs.LG]. p. 371–421.

103. Chapelle O, Schölkopf B, Zien A. Semi-supervised learning MIT Press Cambridge. MIT Press Cambridge;

104. Easson AK, Fatima Z, McIntosh AR. Functional connectivity-based subtypes of individuals with and without autism spectrum disorder. *Network Neuroscience*. MIT Press; 2019; doi: 10.1162/netn_a_00067.

105. Yao D, Calhoun VD, Fu Z, Du Y, Sui J. An ensemble learning system for a 4-way classification of Alzheimer's disease and mild cognitive impairment. *J Neurosci Methods*. J Neurosci Methods; 2018; doi: 10.1016/j.jneumeth.2018.03.008.

106. Efron B. Estimating the Error Rate of a Prediction Rule: Improvement on Cross-Validation. *J Am Stat*

Assoc. Taylor & Francis; 1983; doi: 10.1080/01621459.1983.10477973.

107. Vovk V, Wang R. Combining p-values via averaging. arXiv [math.ST].

Figure 1

Click here to access/download;Figure;fig1_network_revision1.png

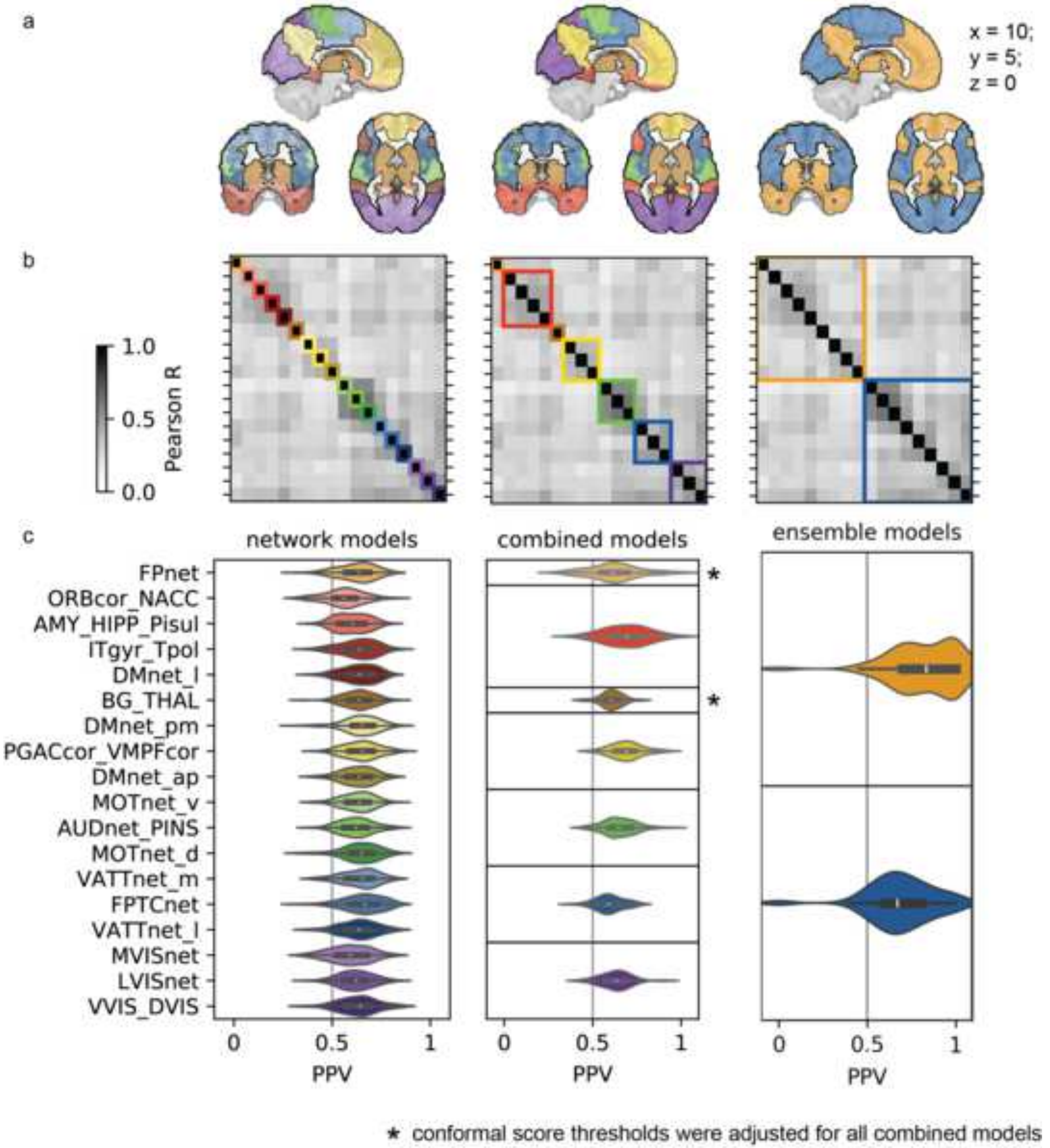

Figure 2

Click here to access/download;Figure;fig2_profile_revision1.png

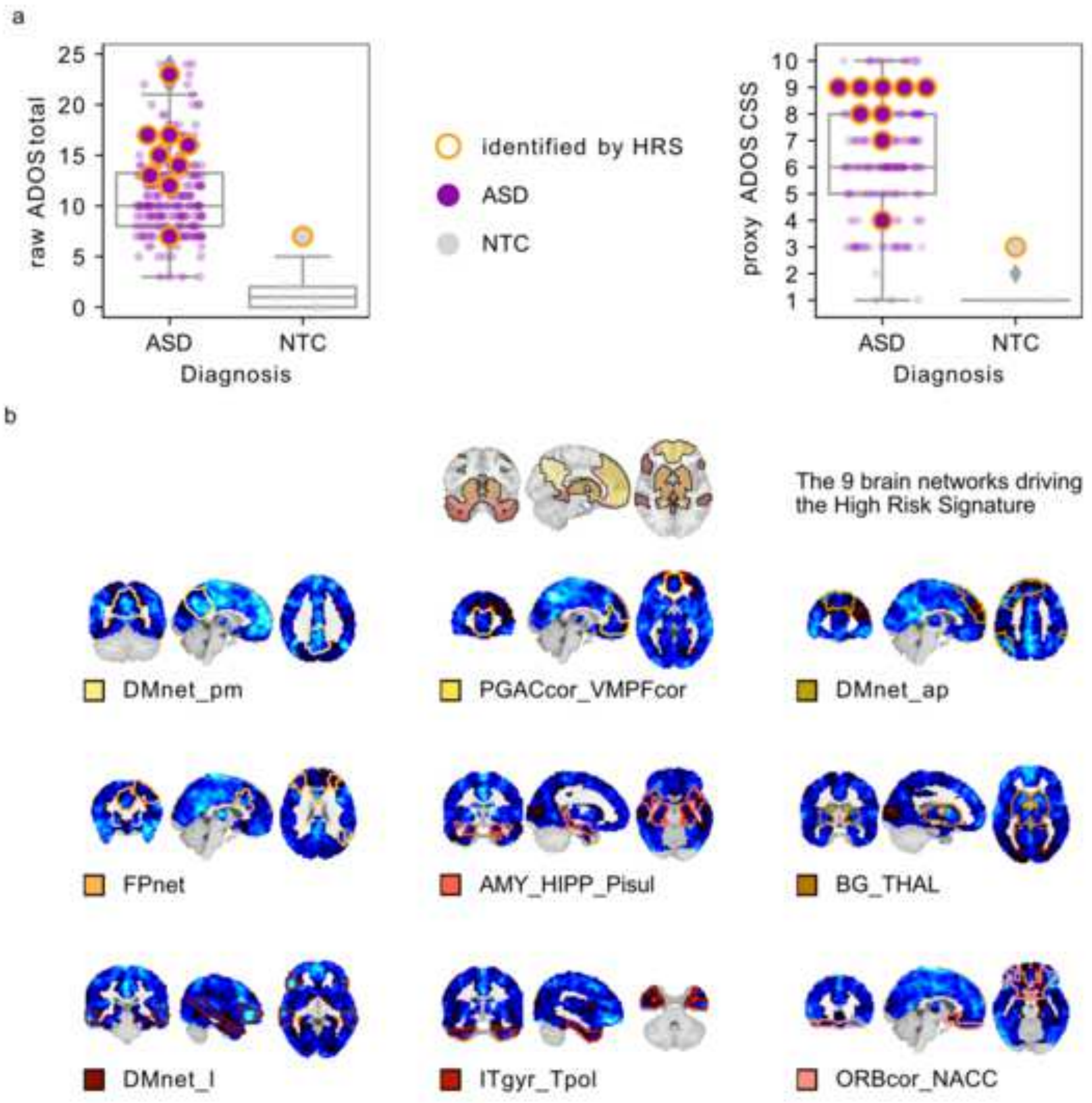

Figure

Click here to access/download;Figure;fig3_nuisance_revision1.png

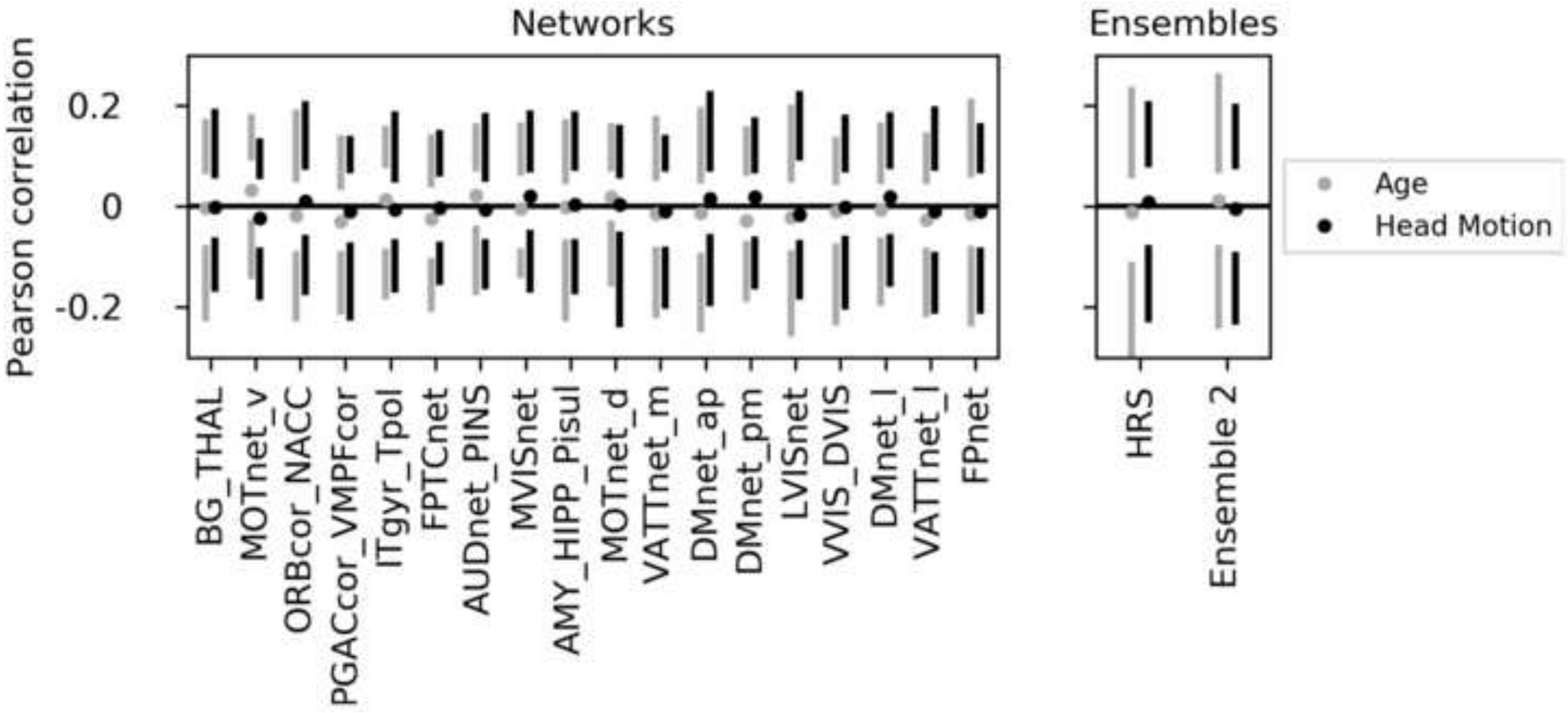

Figure 4

Click here to access/download;Figure;fig4_model_revision1.png

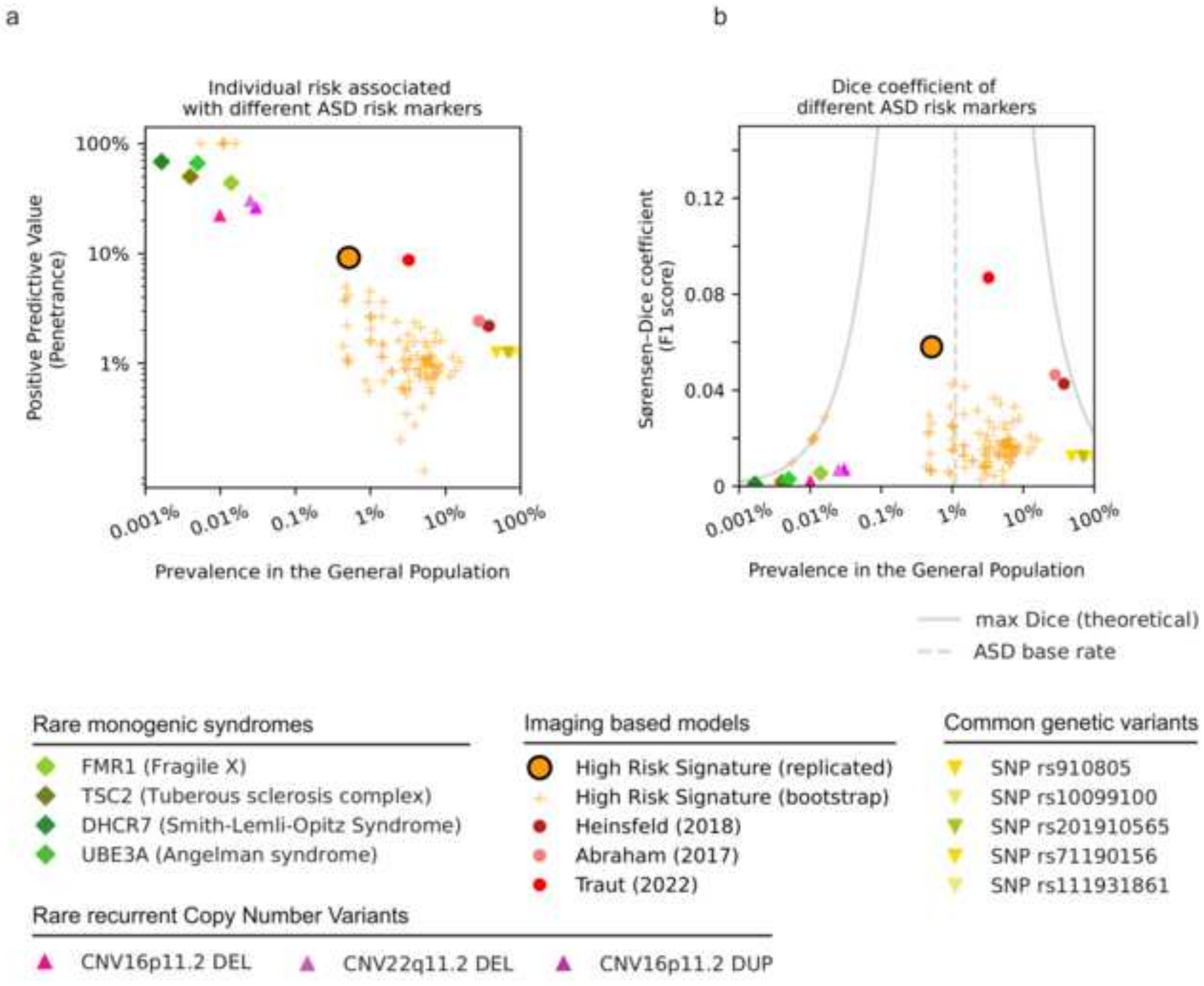

Figure 5

Click here to access/download;Figure;fig5_flowchart.png

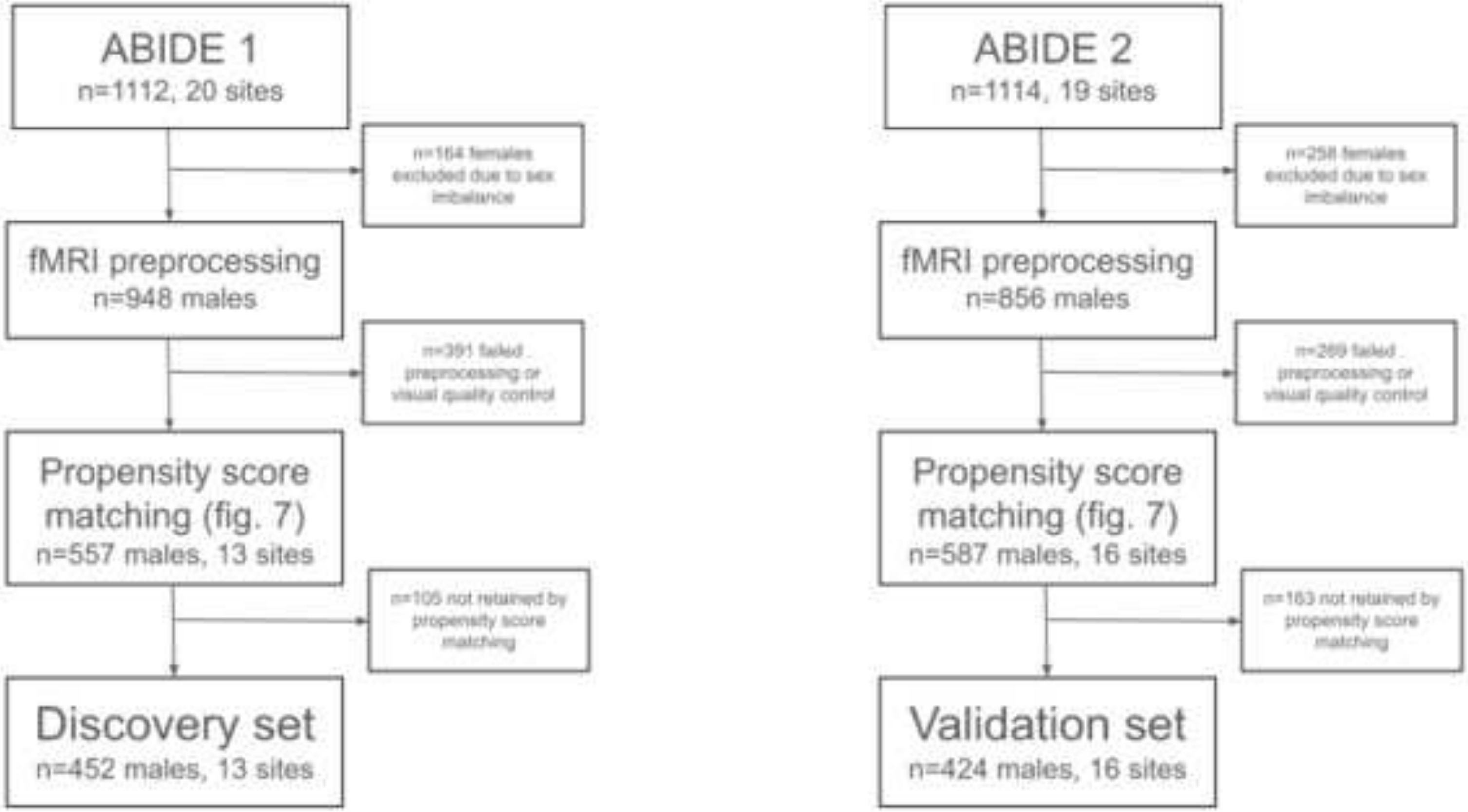

Figure 6

Click here to access/download;Figure;fig6_propensity.png

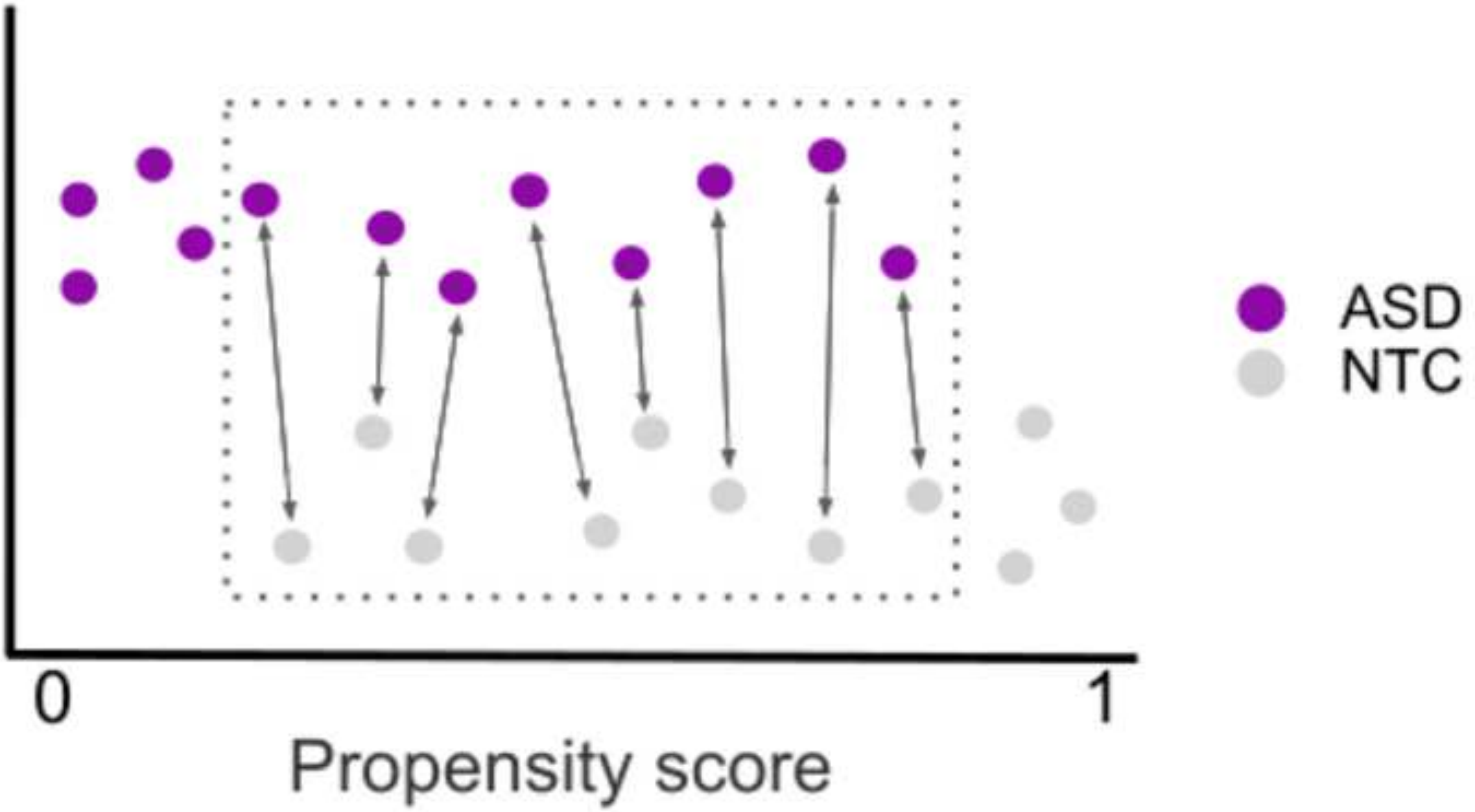

Figure 7

Click here to access/download;Figure;fig7_tcp_schematic.png

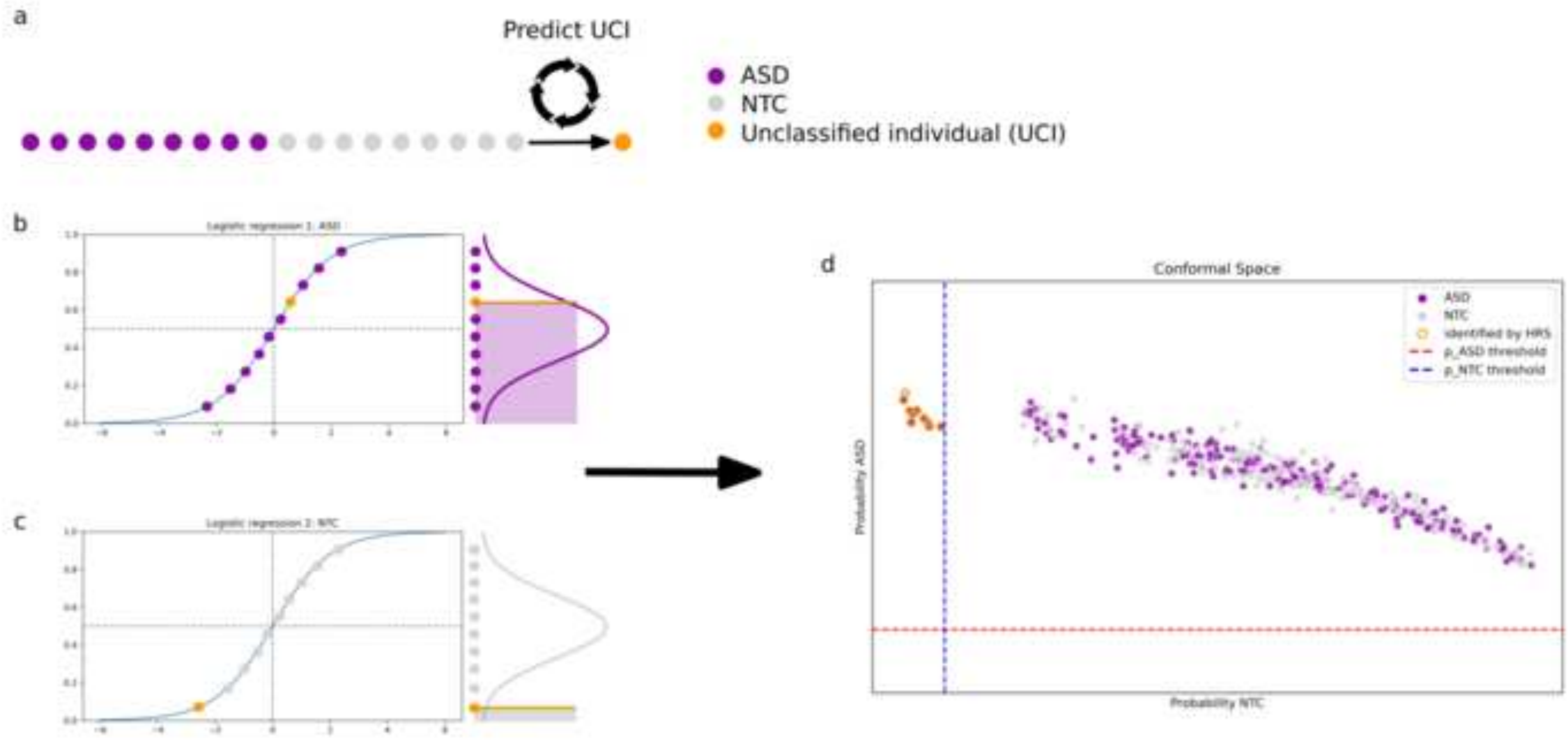

Figure 8

Click here to access/download;Figure;fig8_schematic_performance.png

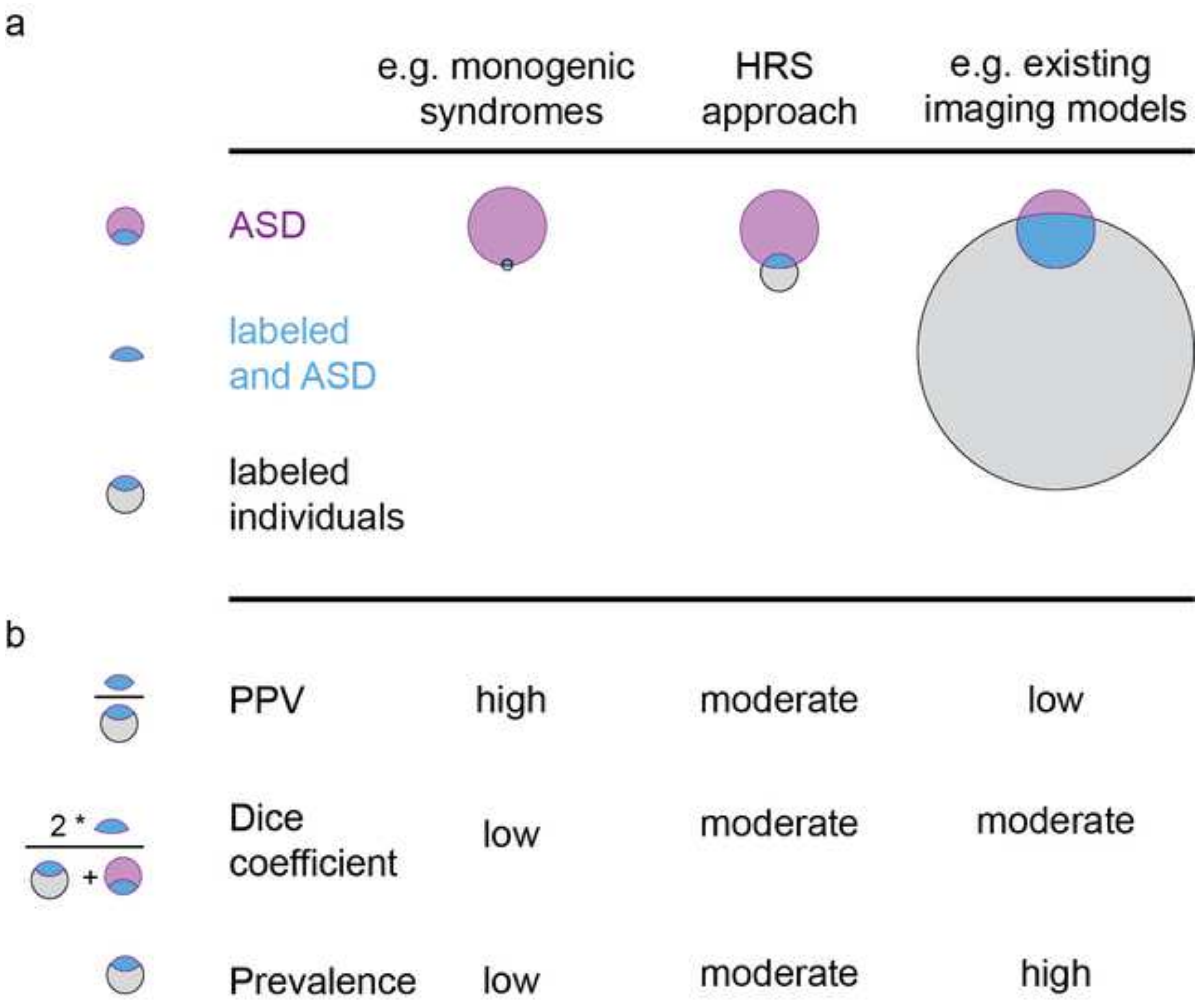

Click here to access/download;Figure;fig1_supp_baseline_revision1.png

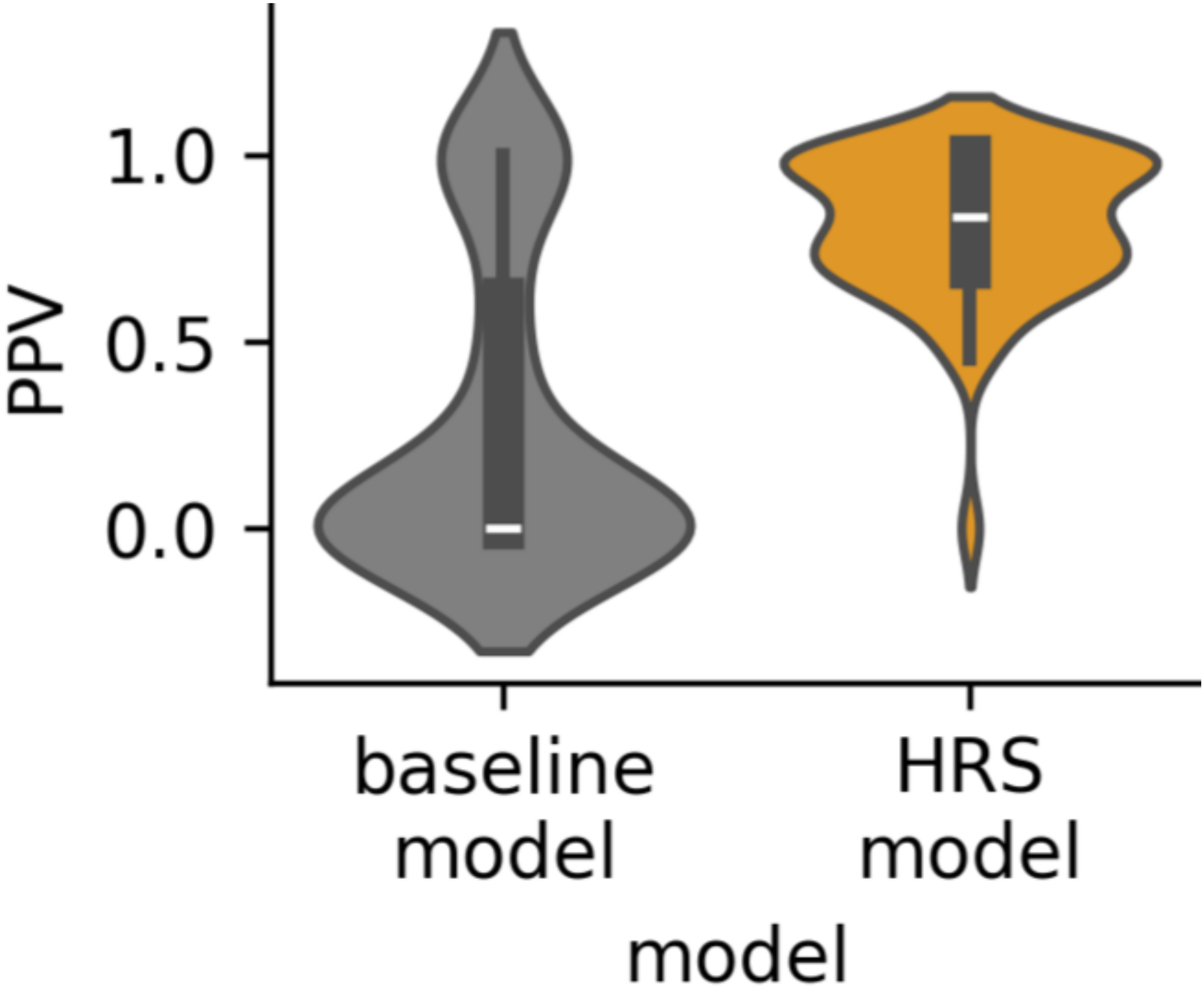

Click here to access/download;Figure;fig2_supp_performance_revision1.png

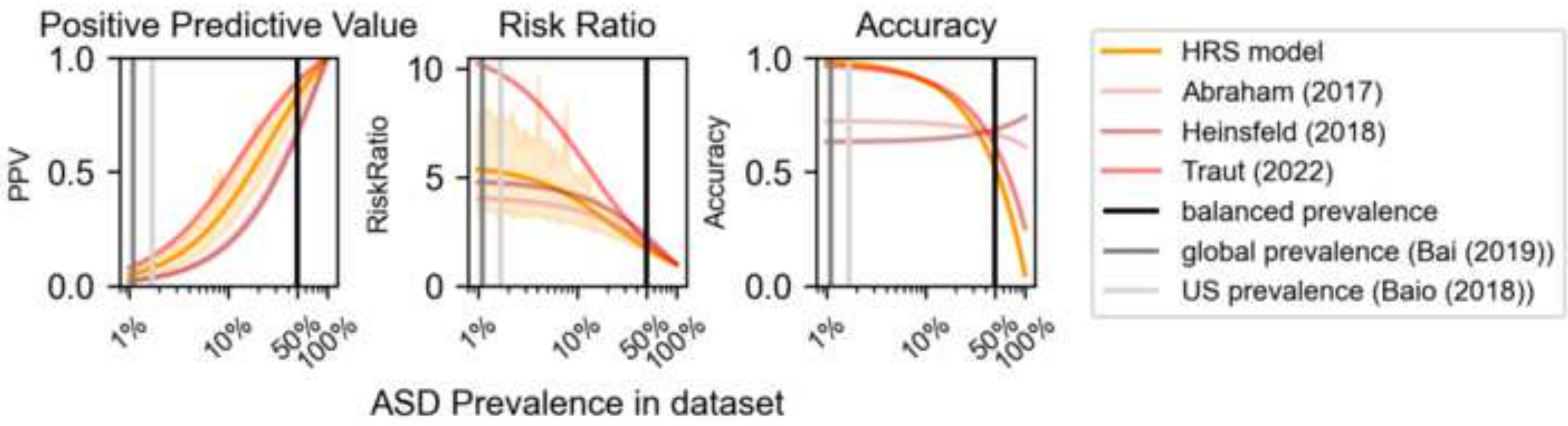

Click here to access/download
Supplementary Material
gigascience_High_Risk_ASD_signature_supplementary_
revision2.pdf
