# Supplementary material for: High-precision machine learning identifies a reproducible functional connectivity signature of autism spectrum diagnosis in a subset of individuals
Source: Gigascience. 2025 Sep 3;14:giaf091. doi: 10.1093/gigascience/giaf091 (PMC12406215; doi:10.1093/gigascience/giaf091)
Supplement: giaf091_Authors_Response_To_Reviewer_Comments_Original_Submission [file giaf091_authors_response_to_reviewer_comments_original_submission.pdf]

Author's Response To Reviewer Comments

Dear Dr Zauner,

We thank you and reviewers for your time reviewing our manuscript. We have addressed the comments and feel the manuscript is much improved. Please see our responses below, with text highlighted in the manuscript. Please note that in the process of making the results fully reproducible and re-running analyses, some values changed by a few percentage points. This does not affect the conclusions of the paper.

We look forward to hearing from you.

Best,

Dr Natasha Clarke,
on behalf of all authors

Editor
Computational workflows should be registered in workflowhub.eu and the [E1.2] DOIs cited in the relevant places in the manuscript. These will facilitate tracking, reproducibility and re-use of your tool.

We have registered the computational workflow on workflowhub.eu and added the DOI to the manuscript.
Reviewer #1
Comments
Here are some questions and comments to help the author improving the manuscripts:

(R1.1) The expression in line 105 "optimising the PPV of each individual prediction" is not accurate.

We have corrected this line so it now reads “To achieve this we reframe the traditional prediction problem from optimising the prediction accuracy across all individuals with ASD to instead optimising the PPV, by focusing on individuals…”

(R1.2) What is the reason to use the median PPV across 100 bootstrap samples, instead of mean PPV?

This decision was taken as the PPV values across the bootstrap samples are not normally distributed (please see Figure1C). We have added this explanation to the manuscript, line 199.

(R1.3) The method of how individual networks were clustered is not mentioned in the descriptions of Figure 1, please claim it.

We have added the method to the figure description.

(R1.4) The comparisons of the PPV between or within network groups in section " Functionally similar brain networks predict correlated conformal scores" and "Ensemble of transmodal networks forms high risk ASD signature" are now presented separately in the text, please organize it into a table.

We have created a table, please see table 1.

(R1.5) What is the relationship between ensemble 1 and HRS? The representation of it in the manuscript is not consistent, please change the "HRS" in Figure 3 into "Ensemble 1".

We applied the term high risk signature (HRS) to the ensemble 1 predictor, given its superiority in performance, that we believe confers a high risk of ASD. We therefore refer to it as ensemble 1 until this is determined in the section “Ensemble of transmodal networks forms high risk ASD signature”. We have amended the text to only use “HRS” in all following sections, and updated Figure 3.

(R1.6) The results of Figure 2a and Figure 2b are far apart, please rearrange them.

These have been rearranged.

(R1.7) Please add the p value for each correlation and claim which correlation analysis is used in "Clinical diagnosis and severity estimates", are these comparisons significant?

We have stipulated that this was Pearson’s correlation and conducted statistical testing to confirm the comparisons are significant.

(R1.8) The relationship between the Figure 7 (A) and its description is not clear. And it is not obvious to represent NTC as grey node.

We have clarified the figure description. We chose grey since many other colours are used in this figure already (e.g. blue, oranges and browns). Green is not appropriate. Therefore we appreciate this feedback but on this occasion have chosen to use grey, and will consider other options for future work.

(R1.9) Please claim the method used to regress the nuisance covariates.

We have stipulated that this was ordinary least squares regression.

(R1.10) Since these analyses only include male individuals, please add the discussion whether the results are similar with the previous researches that also only contain male participants.

We have added this to the discussion, line 289.
Reviewer #2
Comments
R2.1. The background section focuses too much on genetics, which is not closely related to the main research content (predicting high-risk ASD individuals using brain imaging). It is recommended to focus more on the role of brain imaging in ASD prediction and strengthen comparisons with previous brain imaging studies to highlight the improvements and advantages of the proposed model.

We have edited the section on genetics to ensure brevity, and added more of a focus on previous brain imaging studies. We do believe that the genetics component plays a central role in understanding heterogeneity of ASD, and is often neglected in the neuroimaging literature. This is particularly the case for understanding subtypes, which has been heavily informed by the field of genetics. We have edited the introduction and hope this link is now clearer.

R2.2. The abstract lacks clarity in describing the methodology and key findings. For example, the specific principles of the "transductive conformal prediction" method are not explained, and the specific brain networks or regions involved in functional connectivity weakening are not identified. Additionally, the "transmodal brain networks" are not specified in terms of which modalities are included. It is recommended to include these details to provide readers with a clearer understanding of the methodology and core findings of the study.

We have added detail and clarity to the abstract, stipulating the connectivity and TCP methods, and results, in more detail. To meet the word count for the abstract we edited the rest of the abstract also.

R2.3. The background section mentions the application of resting-state functional connectivity (FC) in ASD research, but the review of previous studies using MRI for ASD prediction is overly brief and lacks detailed discussion of existing methods. The authors should provide more information about different functional indices (such as fALFF, functional connectivity (FC), dynamic FC, causal functional connectivity, etc.) and machine learning methods (such as support vector machines, deep learning, multimodal fusion, etc.) applied in ASD prediction, as well as evaluate the effectiveness and limitations of these approaches.

We have added more detail in the background section (paragraph 4) and improved our evaluation of the

effectiveness and limitations of previous research (paragraphs 5 and 6).

R2.4. Additionally, the background should more comprehensively discuss the challenges faced by existing studies, such as model generalizability, population heterogeneity, and diagnostic subtypes of the disease. The authors merely summarize these issues with the term "heterogeneity," without adequately addressing the diversity and challenges of previous research. To enhance the theoretical depth of the paper, it is recommended to include a more detailed review and evaluation of these studies, in order to clearly demonstrate the novelty and improvements of the current research in these areas.

We have added more on these issues in paragraphs 5 and 6.

R2.5. The background mentions the use of "transductive conformal prediction" but does not explain why this method was chosen or its advantages. It is recommended that the authors briefly introduce the basic principles of this method, particularly how it addresses issues like model generalizability and population heterogeneity. Additionally, the background should discuss its application and effectiveness in other diseases to help readers understand its potential in ASD prediction. This would strengthen the theoretical foundation of the paper.

We have addressed this comment in line 138.

R2.6. The article refers to the "integration of different brain networks" as "different modalities," but in fact, all the brain network data come from the same modality—functional magnetic resonance imaging (fMRI). Therefore, it is incorrect to refer to the combination of multiple brain networks as "different modalities." It is recommended to change this to "combination of different brain networks" or "multi-network integration" to avoid conceptual confusion.

We are unable to find any instances of “different modalities” or any references to modalities in the text. Please could the reviewer clarify which section(s) this appears in?

R2.7. In the results section, although it is mentioned that individuals identified by the high-risk signature tend to have more severe symptoms, the sample size is small, and no further correlation analysis has been performed. It is recommended that the authors further analyze and discuss the relationship between symptom severity and functional connectivity patterns to enhance the interpretability of the results. Additionally, providing more details on how the correlation between symptoms and brain connectivity is quantified would help readers better understand the specific impact of these functional connectivity patterns on ASD symptoms.

We computed the correlation between symptom severity (ADOS proxy scores) and the ASD conformality score, added to the results section (line 284). We also updated the discussion to include interpretation of this result (line 373).

R2.8. The step of excluding irrelevant variables is more of a standard data preprocessing procedure and should be described in the Methods section. In the Results section, it can be briefly mentioned how this process impacts the results, without going into excessive detail, to maintain the clarity and focus of the results section.

We would appreciate it if the reviewer can clarify this comment. We regress nuisance covariates, as detailed in the Methods section "Regression of nuisance covariates". Then, in the results section, we report analysis confirming that these did not influence the model ("Conformal prediction not driven by nuisance covariates"). It is not clear which portion(s) of the text the reviewer is referring to.

R2.9. The discussion section compares the proposed method with traditional imaging models and ensemble models from the ASD prediction challenge, but the depth and breadth of the comparison are limited. It is recommended that the authors include comparisons with other existing brain imaging-based predictive models or different machine learning methods. Additionally, a more in-depth discussion of the trade-off between sensitivity and specificity is needed, explaining why increasing specificity at the cost of sensitivity is valuable. This would better highlight the innovation and practical potential of the proposed method.

We have added more on the specificity-sensitivity trade-off, lines 329-336. We have added more detail on existing literature under “Model performance” and in lines 342-348. However we would like to note that since previous studies generally do not use methods that optimise PPV, the scope of comparing performance is limited. We therefore focus on comparing the network results to previous literature in the section “Individuals identified by the signature tend to have severe symptoms, and underconnectivity” .

R2.10. In the conclusion section, the phrase "Decomposing the autism spectrum bit by bit" may feel vague or insufficiently specific to the reader. It is recommended to rephrase this part to more clearly convey how the study reveals the diversity of ASD through functional connectivity features and how it supports understanding the multiple etiological pathways of ASD.

We have rewritten this section to provide more context.

R2.11. In the abstract and conclusion sections, the phrase "decompose part of the heterogeneity of ASD" may feel vague or insufficiently specific to the reader.

We have reworded the abstract to make it clearer.

Editorial Manager®

Cookie Preference Center

We use cookies which are necessary to make our site work. We may also use additional cookies to analyze, improve and personalize our content and your digital experience. For more information, see our Cookie Policy.

You may choose not to allow some types of cookies. However, blocking some types may impact your experience of our site and the services we are able to offer. See the different category headings below to find out more or change your settings.

Allow all

Manage Consent Preferences

Strictly Necessary Cookies

Always active

These cookies are necessary for the website to function and cannot be switched off in our systems. They are usually only set in response to actions made by you which

amount to a request for services, such as setting your privacy preferences, logging in or filling in forms. You can set your browser to block or alert you about these cookies, but some parts of the site will not then work.

Functional Cookies

These cookies enable the website to provide enhanced functionality and personalisation. They may be set by us or by third party providers whose services we have added to our pages. If you do not allow these cookies then some or all of these services may not function properly.

Performance Cookies

These cookies allow us to count visits and traffic sources so we can measure and improve the performance of our site. They help us to know which pages are the most and least popular and see how visitors move around the site.

Targeting Cookies

These cookies may be set through our site by our advertising partners. They may be used by those companies to build a profile of your interests and show you relevant adverts on other sites. If you do not allow these cookies, you will experience less targeted advertising.

Cookie List

Clear

Apply Cancel

Consent Leg.Interest

Confirm my choices

Powered by onetrus
