# Supplementary material for: High-precision machine learning identifies a reproducible functional connectivity signature of autism spectrum diagnosis in a subset of individuals
Source: Gigascience. 2025 Sep 3;14:giaf091. doi: 10.1093/gigascience/giaf091 (PMC12406215; doi:10.1093/gigascience/giaf091)
Supplement: giaf091_Authors_Response_To_Reviewer_Comments_Revision_1 [file giaf091_authors_response_to_reviewer_comments_revision_1.pdf]

Author's Response To Reviewer Comments

Dear Dr Zauner,

We are grateful for you and the reviewers for reviewing our manuscript and accepting it for publication (in principle), thank you for your time. We have addressed the few outstanding comments from the reviewers in the paper, and below. Please note we have also updated the title to better describe the paper, and remove the mention of “risk”.

Best,
Dr Natasha Clarke
On behalf of all authors

Reviewer 1
R1.1. The authors have addressed most of the concerns quite well, but the existing literature review need to be improved, some of the following relavant articles on ASD subtypes classification with ensemble learning, and FC prediction were missed, which should be included in the manuscript.
1. S Qi, R Morris, et al. 2020. Common and unique multimodal covarying patterns in autism spectrum disorder subtypes. Molecular autism 11, 1-15
2. N Luo, J Sui, et al. 2020. Age-related structural and functional variations in 5,967 individuals across the adult lifespan. Human brain mapping 41 (7), 1725-1737
3. D Yao, VD Calhoun et al. 2018. An ensemble learning system for a 4-way classification of Alzheimer's disease and mild cognitive impairment. Journal of neuroscience methods 302, 75-81

We thank the reviewer for providing these references which we missed, and have added them to the relevant sections of the manuscript.

Reviewer 2
R2.6. I see that the term “different modalities” wasn’t used in the manuscript. But my original point was about how “transmodal” is used. In neuroscience,
“transmodal”
refers to high-level brain regions that integrate information across functions—not to data types or network relationships. Since the paper discusses between-network connectivity and calls them “transmodal networks,” it might confuse readers into thinking this is about multimodal data. A short explanation would help avoid that.

We appreciate the clarification and have added an explanation in the discussion, line 341-345.

R2.8 Just to clarify—I didn’t mean you didn’t do nuisance regression, or that it wasn’t described in the Methods. My point was that this step is standard preprocessing, and too much emphasis on it in the Results can distract from your main findings. A brief mention is fine, but long explanations in the Results aren’t really necessary.

We thank the reviewer for the clarification. We have amended the section “Conformal prediction not driven by nuisance covariates” so that it is shorter.

Cookie Preference Center

We use cookies which are necessary to make our site work. We may also use additional cookies to analyze, improve and personalize our content and your digital experience. For more information, see our Cookie Policy.

You may choose not to allow some types of cookies. However, blocking some types may impact your experience of our site and the services we are able to offer. See the different category headings below to find out more or change your settings.

Allow all

Manage Consent Preferences

Strictly Necessary Cookies

Always active

These cookies are necessary for the website to function and cannot be switched off in our systems. They are usually only set in response to actions made by you which amount to a request for services, such as setting your privacy preferences, logging in or filling in forms. You can set your browser to block or alert you about these cookies, but some parts of the site will not then work.

Functional Cookies

These cookies enable the website to provide enhanced functionality and personalisation. They may be set by us or by third party providers whose services we have added to our pages. If you do not allow these cookies then some or all of these services may not function properly.

Performance Cookies

These cookies allow us to count visits and traffic sources so we can measure and improve the performance of our site. They help us to know which pages are the most and least popular and see how visitors move around the site.

Targeting Cookies

These cookies may be set through our site by our advertising partners. They may be used by those companies to build a profile of your interests and show you relevant adverts on other sites. If you do not allow these cookies, you will experience less targeted advertising.

Cookie List

Clear

Apply Cancel
Consent Leg.Interest

Confirm my choices

Powered by onetrust
