# Supplementary material for: High-precision machine learning identifies a reproducible functional connectivity signature of autism spectrum diagnosis in a subset of individuals
Source: Gigascience. 2025 Sep 3;14:giaf091. doi: 10.1093/gigascience/giaf091 (PMC12406215; doi:10.1093/gigascience/giaf091)
Supplement: giaf091_Reviewer_1_Report_Original_Submission — Jing Sui -- 11/15/2024 [file giaf091_reviewer_1_report_original_submission.pdf]

Reviewer Report

Title: High-precision machine learning identifies a reproducible functional connectivity signature of autism spectrum diagnosis in a subset of individuals

Version: Original Submission **Date:** 11/15/2024

Reviewer name: Jing Sui

Reviewer Comments to Author:

The purpose of this manuscript is to find an imaging signature to identify ASD patients . 452 male individuals from the ABIDE I were used as the discovery dataset and 557 individuals from the ABIDE II were used as the independent validation dataset. A transductive conformal prediction (TCP) approach was used to identify a high risk signature (HRS) based on functional connectivity patterns across multiple brain networks. By limiting predictions to the most confidently identifiable subset individuals, the positive predictive value (PPV) was enhanced. Then validating the HRS in the validation dataset, including estimating its prevalence and PPV in the general population. Finally, the connectivity and symptom profiles of individuals identified by the HRS were discussed.
Here are some questions and comments to help the author improving the manuscripts:
(1) The expression in line 105 "optimising the PPV of each individual prediction" is not accurate.
(2) What is the reason to use the median PPV across 100 bootstrap samples, instead of mean PPV?
(3) The method of how individual networks were clustered is not mentioned in the descriptions of Figure 1, please claim it.
(4) The comparisons of the PPV between or within network groups in section " Functionally similar brain networks predict correlated conformal scores" and "Ensemble of transmodal networks forms high risk ASD signature" are now presented separately in the text, please organize it into a table.
(5) What is the relationship between ensemble 1 and HRS? The representation of it in the manuscript is not consistent, please change the "HRS" in Figure 3 into "Ensemble 1".
(6) The results of Figure 2a and Figure 2b are far apart, please rearrange them.
(7) Please add the p value for each correlation and claim which correlation analysis is used in "Clinical diagnosis and severity estimates", are these comparisons significant?
(8) The relationship between the Figure 7 (A) and its description is not clear. And it is not obvious to represent NTC as grey node.
(9) Please claim the method used to regress the nuisance covariates.
(10) Since these analyses only include male individuals, please add the discussion whether the results are similar with the previous researches that also only contain male participants.

Methods

Are the methods appropriate to the aims of the study, are they well described, and are necessary controls included? Choose an item.

Conclusions

Are the conclusions adequately supported by the data shown? Choose an item.

Reporting Standards

Does the manuscript adhere to the journal's guidelines on minimum standards of reporting? Choose an item.

Choose an item.

Statistics

Are you able to assess all statistics in the manuscript, including the appropriateness of statistical tests used? Choose an item.

Quality of Written English

Please indicate the quality of language in the manuscript: Choose an item.

Declaration of Competing Interests

Please complete a declaration of competing interests, considering the following questions:

- Have you in the past five years received reimbursements, fees, funding, or salary from an organisation that may in any way gain or lose financially from the publication of this manuscript, either now or in the future?
- Do you hold any stocks or shares in an organisation that may in any way gain or lose financially from the publication of this manuscript, either now or in the future?
- Do you hold or are you currently applying for any patents relating to the content of the manuscript?
- Have you received reimbursements, fees, funding, or salary from an organization that holds or has applied for patents relating to the content of the manuscript?
- Do you have any other financial competing interests?
- Do you have any non-financial competing interests in relation to this paper?

If you can answer no to all of the above, write 'I declare that I have no competing interests' below. If your reply is yes to any, please give details below.

I declare that I have no competing interests.

I agree to the open peer review policy of the journal. I understand that my name will be included on my report to the authors and, if the manuscript is accepted for publication, my named report including any attachments I upload will be posted on the website along with the authors' responses. I agree for my

report to be made available under an Open Access Creative Commons CC-BY license (http://creativecommons.org/licenses/by/4.0/). I understand that any comments which I do not wish to be included in my named report can be included as confidential comments to the editors, which will not be published.

Choose an item.

To further support our reviewers, we have joined with Publons, where you can gain additional credit to further highlight your hard work (see: https://publons.com/journal/530/gigascience). On publication of this paper, your review will be automatically added to Publons, you can then choose whether or not to claim your Publons credit. I understand this statement.

Yes Choose an item.
