# Supplementary material for: High-precision machine learning identifies a reproducible functional connectivity signature of autism spectrum diagnosis in a subset of individuals
Source: Gigascience. 2025 Sep 3;14:giaf091. doi: 10.1093/gigascience/giaf091 (PMC12406215; doi:10.1093/gigascience/giaf091)
Supplement: giaf091_Reviewer_2_Report_Original_Submission — Yuan Feng -- 12/14/2024 [file giaf091_reviewer_2_report_original_submission.pdf]

Reviewer Report

Title: High-precision machine learning identifies a reproducible functional connectivity signature of autism spectrum diagnosis in a subset of individuals

Version: Original Submission **Date:** 12/17/2024

Reviewer name: Yuan Feng

Reviewer Comments to Author:

1. The background section focuses too much on genetics, which is not closely related to the main research content (predicting high-risk ASD individuals using brain imaging). It is recommended to focus more on the role of brain imaging in ASD prediction and strengthen comparisons with previous brain imaging studies to highlight the improvements and advantages of the proposed model.
2. The abstract lacks clarity in describing the methodology and key findings. For example, the specific principles of the "transductive conformal prediction" method are not explained, and the specific brain networks or regions involved in functional connectivity weakening are not identified. Additionally, the "transmodal brain networks" are not specified in terms of which modalities are included. It is recommended to include these details to provide readers with a clearer understanding of the methodology and core findings of the study.
3. The background section mentions the application of resting-state functional connectivity (FC) in ASD research, but the review of previous studies using MRI for ASD prediction is overly brief and lacks detailed discussion of existing methods. The authors should provide more information about different functional indices (such as fALFF, functional connectivity (FC), dynamic FC, causal functional connectivity, etc.) and machine learning methods (such as support vector machines, deep learning, multimodal fusion, etc.) applied in ASD prediction, as well as evaluate the effectiveness and limitations of these approaches.
4. Additionally, the background should more comprehensively discuss the challenges faced by existing studies, such as model generalizability, population heterogeneity, and diagnostic subtypes of the disease. The authors merely summarize these issues with the term "heterogeneity," without adequately addressing the diversity and challenges of previous research. To enhance the theoretical depth of the paper, it is recommended to include a more detailed review and evaluation of these studies, in order to clearly demonstrate the novelty and improvements of the current research in these areas.
5. The background mentions the use of "transductive conformal prediction" but does not explain why this method was chosen or its advantages. It is recommended that the authors briefly introduce the basic principles of this method, particularly how it addresses issues like model generalizability and population heterogeneity. Additionally, the background should discuss its application and effectiveness in other diseases to help readers understand its potential in ASD prediction. This would strengthen the theoretical foundation of the paper.
6. The article refers to the "integration of different brain networks" as "different modalities," but in fact, all the brain network data come from the same modalityâ€”functional magnetic resonance imaging (fMRI). Therefore, it is incorrect to refer to the combination of multiple brain networks as "different

modalities." It is recommended to change this to "combination of different brain networks" or "multi-network integration" to avoid conceptual confusion.
7. In the results section, although it is mentioned that individuals identified by the high-risk signature tend to have more severe symptoms, the sample size is small, and no further correlation analysis has been performed. It is recommended that the authors further analyze and discuss the relationship between symptom severity and functional connectivity patterns to enhance the interpretability of the results. Additionally, providing more details on how the correlation between symptoms and brain connectivity is quantified would help readers better understand the specific impact of these functional connectivity patterns on ASD symptoms.
8. The step of excluding irrelevant variables is more of a standard data preprocessing procedure and should be described in the Methods section. In the Results section, it can be briefly mentioned how this process impacts the results, without going into excessive detail, to maintain the clarity and focus of the results section.
9. The discussion section compares the proposed method with traditional imaging models and ensemble models from the ASD prediction challenge, but the depth and breadth of the comparison are limited. It is recommended that the authors include comparisons with other existing brain imaging-based predictive models or different machine learning methods. Additionally, a more in-depth discussion of the trade-off between sensitivity and specificity is needed, explaining why increasing specificity at the cost of sensitivity is valuable. This would better highlight the innovation and practical potential of the proposed method.
10. In the conclusion section, the phrase "Decomposing the autism spectrum bit by bit" may feel vague or insufficiently specific to the reader. It is recommended to rephrase this part to more clearly convey how the study reveals the diversity of ASD through functional connectivity features and how it supports understanding the multiple etiological pathways of ASD.
11. In the abstract and conclusion sections, the phrase "decompose part of the heterogeneity of ASD" may feel vague or insufficiently specific to the reader.

Methods

Are the methods appropriate to the aims of the study, are they well described, and are necessary controls included? Choose an item.

Conclusions

Are the conclusions adequately supported by the data shown? Choose an item.

Reporting Standards

Does the manuscript adhere to the journal's guidelines on minimum standards of reporting? Choose an item.

Choose an item.

Statistics

Are you able to assess all statistics in the manuscript, including the appropriateness of statistical tests used? Choose an item.

Quality of Written English

Please indicate the quality of language in the manuscript: Choose an item.

Declaration of Competing Interests

Please complete a declaration of competing interests, considering the following questions:

- Have you in the past five years received reimbursements, fees, funding, or salary from an organisation that may in any way gain or lose financially from the publication of this manuscript, either now or in the future?
- Do you hold any stocks or shares in an organisation that may in any way gain or lose financially from the publication of this manuscript, either now or in the future?
- Do you hold or are you currently applying for any patents relating to the content of the manuscript?
- Have you received reimbursements, fees, funding, or salary from an organization that holds or has applied for patents relating to the content of the manuscript?
- Do you have any other financial competing interests?
- Do you have any non-financial competing interests in relation to this paper?

If you can answer no to all of the above, write 'I declare that I have no competing interests' below. If your reply is yes to any, please give details below.

I declare that I have no competing interests

I agree to the open peer review policy of the journal. I understand that my name will be included on my report to the authors and, if the manuscript is accepted for publication, my named report including any attachments I upload will be posted on the website along with the authors' responses. I agree for my report to be made available under an Open Access Creative Commons CC-BY license (http://creativecommons.org/licenses/by/4.0/). I understand that any comments which I do not wish to be included in my named report can be included as confidential comments to the editors, which will not be published.

Choose an item.

To further support our reviewers, we have joined with Publons, where you can gain additional credit to further highlight your hard work (see: https://publons.com/journal/530/gigascience). On publication of this paper, your review will be automatically added to Publons, you can then choose whether or not to claim your Publons credit. I understand this statement.

Yes Choose an item.
